# Scrambled eggs: Proteomic portraits and novel biomarkers of egg quality in zebrafish (*Danio rerio*)

**DOI:** 10.1371/journal.pone.0188084

**Published:** 2017-11-16

**Authors:** Ozlem Yilmaz, Amélie Patinote, Thao Vi Nguyen, Emmanuelle Com, Regis Lavigne, Charles Pineau, Craig V. Sullivan, Julien Bobe

**Affiliations:** 1 Laboratory of Fish Physiology and Genomics, INRA UR1037, Rennes Cedex, France; 2 Protim, Inserm U1085, Irset, Rennes Cedex, France; 3 Carolina AquaGyn, Raleigh, North Carolina, United States of America; Pacific Northwest National Laboratory, UNITED STATES

## Abstract

Egg quality is a complex biological trait and a major determinant of reproductive fitness in all animals. This study delivered the first proteomic portraits of egg quality in zebrafish, a leading biomedical model for early development. Egg batches of good and poor quality, evidenced by embryo survival for 24 h, were sampled immediately after spawning and used to create pooled or replicated sample sets whose protein extracts were subjected to different levels of fractionation before liquid chromatography and tandem mass spectrometry. Obtained spectra were searched against a zebrafish proteome database and detected proteins were annotated, categorized and quantified based on normalized spectral counts. Manually curated and automated enrichment analyses revealed poor quality eggs to be deficient of proteins involved in protein synthesis and energy and lipid metabolism, and of some vitellogenin products and lectins, and to have a surfeit of proteins involved in endo-lysosomal activities, autophagy, and apoptosis, and of some oncogene products, lectins and egg envelope proteins. Results of pathway and network analyses suggest that this aberrant proteomic profile results from failure of oocytes giving rise to poor quality eggs to properly transit through final maturation, and implicated Wnt signaling in the etiology of this defect. Quantitative comparisons of abundant proteins in good versus poor quality eggs revealed 17 candidate egg quality markers. Thus, the zebrafish egg proteome is clearly linked to embryo developmental potential, a phenomenon that begs further investigation to elucidate the root causes of poor egg quality, presently a serious and intractable problem in livestock and human reproductive medicine.

## Introduction

Poor gamete quality, a major concern in human reproductive medicine and livestock production, is also common in fishes and is an important limiting factor in global aquaculture [1]. Fish egg quality is defined by the ability to be fertilized and support development of normal embryos and larvae [2, 3]. This ‘developmental competence’ can be affected by intrinsic factors such as maternal age and genetics, as well as environmental factors arising from husbandry practices [4, 5, 6]. The mechanisms by which these various factors influence egg quality are generally not well understood. Diverse parameters such as egg diameter, morphology, buoyancy, yolk composition and fertilization rate, blastomere cell symmetry, activity of key enzymes in intermediary metabolism, and hatching rate have been used as fish egg quality criteria [6]. However, we know little about the causes and extent of variation in these parameters within and among females, between populations, and among different species spawning disparate types of eggs. Additionally, all of these parameters identify egg quality *a posteriori*; truly predictive biomarkers that may directly arise from and identify molecular processes determining egg quality remain to be identified.

Fishes exhibit a diverse array of reproductive modes and life histories, but oocytes of all species employ the same fundamental molecular processes to pass through a series of defined stages leading to production of fertilizable ova that can support offspring development [7, 8]. The vast majority of fishes are oviparous; their offspring develop *in ovo* independent of the mother and are totally dependent upon the contents of the yolk to sustain development. Recent investigations have focused on these molecular contents as potential determinants of egg quality that may be widely conserved among fishes and vertebrates in general [9, 3, 10]. Special attention has been paid to the earliest stages of development, during which times most losses are observed in cultured fishes.

Maternal mRNAs deposited in eggs direct vertebrate development until activation of zygotic transcription around mid-blastula stage [11, 12]. Certain maternal transcripts may exhibit differential abundance in fish eggs of varying quality [13]. Egg quality may also be predicted by subtle differences in the pattern of expression of large suites of maternal genes, constituting a ‘transcriptomic fingerprint’ of egg quality [14]. While the nature and abundance of maternal mRNAs appears to be important to fertility and embryo developmental competence, there has been little consistency between studies regarding the identity of relevant transcripts in different species [13]. Additionally, there seems to be little consistency between transcript abundance and product protein expression during development [15], which complicates interpretation of the transcriptomic findings. Furthermore, modification of proteins after their uptake into growing oocytes plays crucial roles in many aspects of oogenesis. These roles cannot be followed by genomic or transcriptomic technologies, necessitating application of proteomics for their elucidation. While proteomic profiling has been widely employed to study the cell biology of oocytes in many species, including humans, mice, pigs, fish and insects [16], it has rarely been applied to studies of fish egg quality. The spectrum of proteins present in eggs was partly evaluated and a few proteins associated with egg quality were identified in studies of rainbow trout (*Oncorhynchus mykiss*) [17], European sea bass (*Dicentrarchus labrax*) [18], Eurasian perch (*Perca fluviatilis*) [19], and hapuku (*Polyprion oxygeneios*) [20].

Zebrafish are an established biomedical model for research on developmental biology and an emerging model for fertility research [21]. They are a promising species for investigating proteomic determinants of egg quality because they are small, easily bred in the laboratory with short generation time, and lay large eggs of various quality every few days, with external fertilization of the transparent eggs in which embryonic development is easily observed [22]. A reference genome sequence is available, providing the needed proteome database for this line of research in zebrafish, which are the only teleost for which any detailed study of the egg proteome has been performed [23]. The proteome of zebrafish ovary has been characterized and compared to mRNA repertoires and transcript abundances [15], and studies have been conducted on proteomics of developing zebrafish embryos [24, 25, 26, 27]. However, no global proteomic analysis of eggs of different quality grades has been undertaken to identify physiological processes underpinning egg quality at the molecular level or to discover protein markers predictive of egg quality in this species. Therefore, the objectives of the present study were to compare the proteome profiles of good versus poor quality eggs, to identify potential egg quality marker proteins, and to shed light on the molecular processes by which these profiles and proteins may influence egg quality in zebrafish.

## Results

Zebrafish whose freshly spawned eggs were subjected to proteomic analysis in this study exhibited considerable variance in fecundity and in the proportion of eggs producing embryos surviving to 24 hours post spawning (hps), with no apparent relationship between fecundity and embryo survival (Table 1). For certain spawns, cumulative percent embryo survival decreased to low levels up to 24 hps, but no changes in survival were observed thereafter for up to 72 hps (Table 1). Therefore, survival of normal embryos to 24 hps was utilized as the measure of egg quality in this study. Spawns with a survival rate of normal embryos to 24 hps of >90% were considered to contain good quality eggs and spawns with a survival rate of normal embryos to 24 hps of <30% were considered to contain poor quality eggs. The actual 24 hps survival rates observed were >98% for good quality eggs and <17% for poor quality eggs (Table 1). It was observed that both good and poor quality eggs rapidly underwent cortical reaction and completed chorion hardening by the time they were sampled for proteomics analysis immediately after spawning. However, at 2–3 hps poor quality eggs invariably had a high incidence of abnormal embryos with asymmetric cell cleavage and/or developmental arrest at early cleavage stages; most embryos in poor quality eggs that survived to 8 hps were of this type (Table 1) and none of these survived to 24 hps. At 24 hps and later time points, mean survival of poor quality eggs in the Pooled Samples Experiment (7.2 ± 3.3%) and the Multiple Samples Experiment (8.7 ± 3.5%) was significantly less that of good quality eggs (99.6 ± 0.4% and 100 ± 0%, respectively) (Tukey-Duckworth test, *P*<0.05).

**Table 1:** Spawning performance of females whose eggs were subjected to proteomic analyses.

Experiment	Egg quality type	Female number	Eggs total(N)	Eggs incubated	% Intact eggs 2–3 hps	% Survival 8 hps	% Survival 24 hps	% Survival48 hps	% Survival 72 hps
**Pooled Samples**	*Good*	1	115	60	100	100 (1.7)	**98.3 (0)**	98.3 (0)	98.3 (0)
		2	216	60	100	100 (0)	**100 (0)**	100 (0)	100 (0)
		3	355	60	100	100 (0)	**100 (0)**	100 (0)	100 (0)
		4	401	60	100	100 (0)	**100 (0)**	100 (0)	100 (0)
	*Poor*	5	129	60	96.7*	91.7 (96.4)	**3.3 (0)**	3.3 (0)	3.3 (0)
		6	224	60	100*	0	**0**	0	0
		7	165	53	100*	79.2 (76.2)	**11.3 (0)**	11.3 (0)	11.3 (0)
		8	266	63	100*	17.5 (0)	**14.3 (0)**	14.3 (0)	14.3 (0)
**Multiple Samples**	*Good*	9	401	60	100	100 (0)	**100 (0)**	100 (0)	100 (0)
		10	355	60	100	100 (0)	**100 (0)**	100 (0)	100 (0)
		11	216	60	100	100 (0)	**100 (0)**	100 (0)	100 (0)
		12	124	60	100	100 (0)	**100 (0)**	100 (0)	100 (0)
	*Poor*	13	251	60	100*	0	**0**	0	0
		14	187	60	100*	13.3 (0)	**11.7 (0)**	11.7 (0)	11.7 (0)
		15	481	60	100*	6.7 (0)	**6.7 (0)**	6.7 (0)	6.7 (0)
		16	404	61	96.7*	85.2 (67.3)	**16.4 (0)**	16.4 (0)	16.4 (0)

Shown are the experiment, egg quality type, female number, total number of eggs spawned, number of eggs incubated, percentage (%) of eggs intact at 2–3 hours post spawning (hps), and percentage of eggs yielding embryos surviving to 8, 24, 48 and 72 hps with the percentage of these that were abnormal shown in parentheses. For intact eggs at 2–3 hps, asterisks indicate that a high proportion of abnormal embryos showing asymmetric cell cleavage and/or early developmental arrest were observed but not quantified. The percentage of eggs yielding normal embryos surviving to 24 hps (bold text on gray background) was used to assign females to egg quality groups.

**Fig 1:**
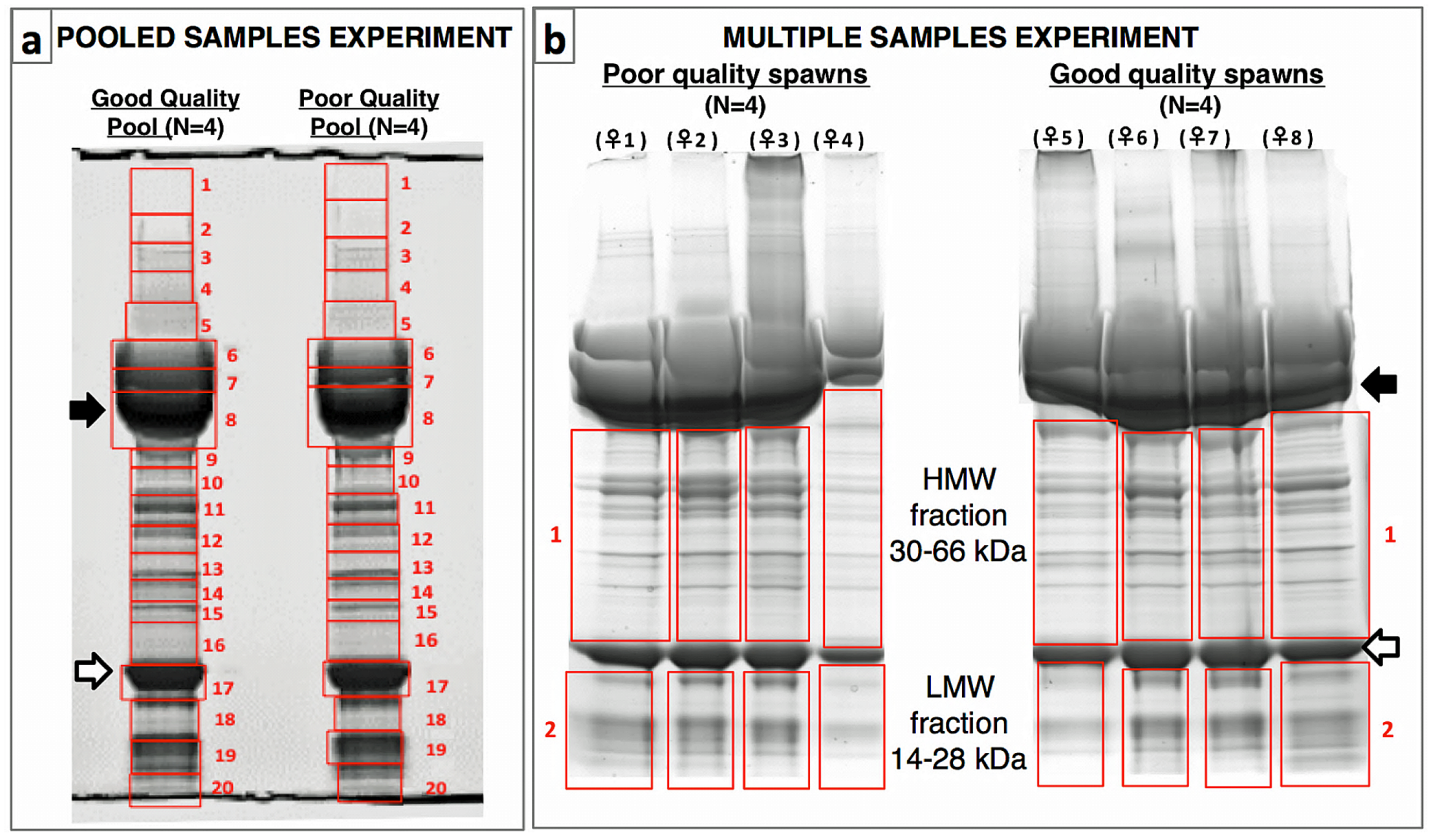
Sample fractionation prior to LC-MS/MS in the egg quality experiments. **Panel a**. Sample fractionation after SDS-PAGE in the Pooled Samples Experiment (N = 4 spawns per egg quality pool). **Panel b**. Sample fractionation after SDS-PAGE in the Multiple Samples Experiment (N = 4 replicate spawns per egg quality type). For each experiment, numbered frames in red indicate fractions excised from the gel and processed separately before submission to LC-MS/MS. Intact lipovitellin (Lv) heavy chain (closed arrows) and light chain (open arrows) were excluded in the Multiple Samples Experiment to better resolve other proteins. In total, 56 egg protein fractions were individually subjected to LC-MS/MS and downstream proteomic analyses. HMW; High molecular weight, LMW; Low molecular weight.

**Fig 2:**
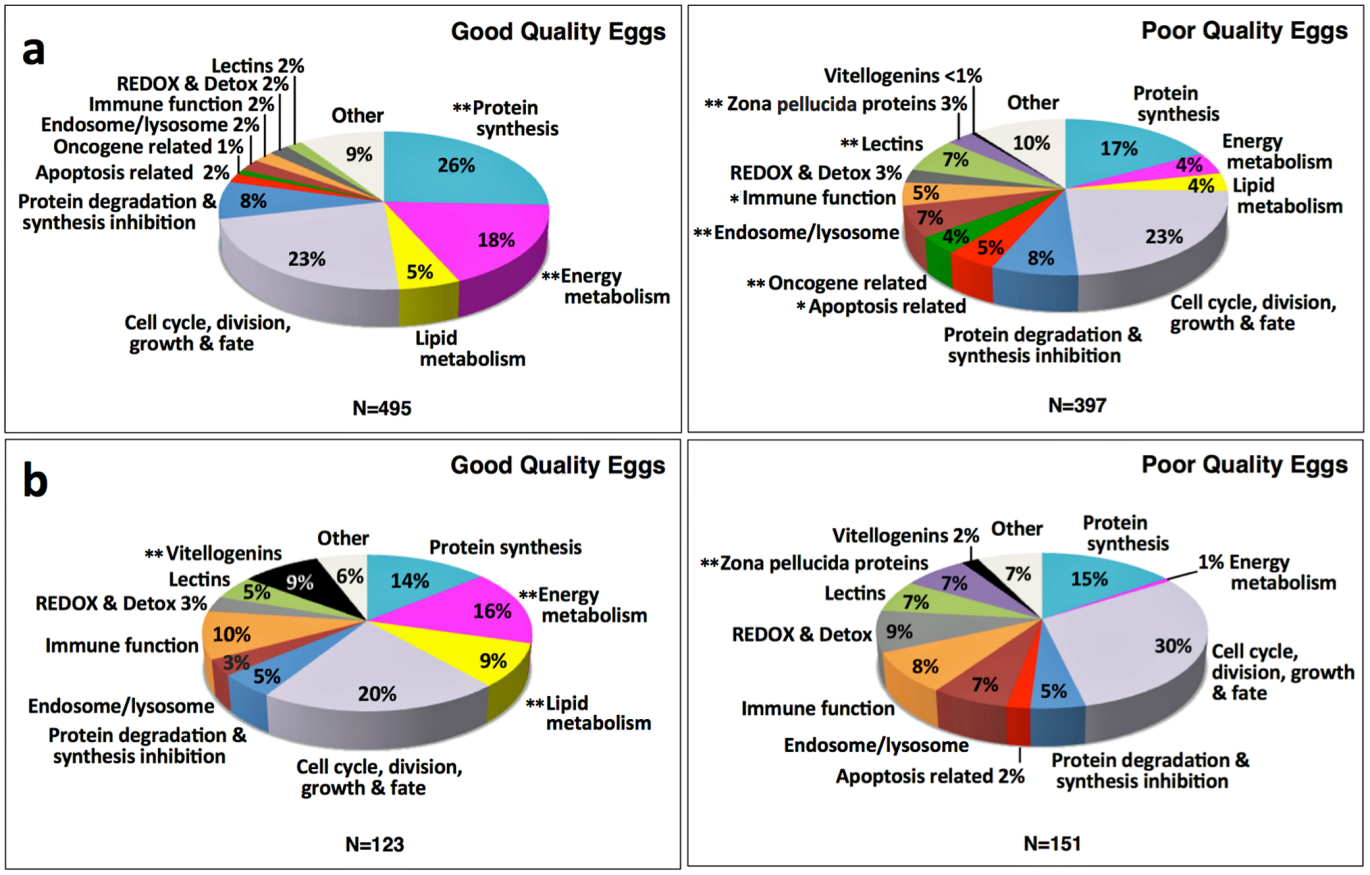
Distribution of proteins up-regulated in good and poor quality zebrafish eggs among functional categories. **Panel a**. Pooled Samples Experiment. **Panel b**. Multiple Samples Experiment. Only proteins common to both egg quality types with a ≥2-fold difference in N-SC between egg quality types, or proteins unique to an egg quality type, were considered to be up-regulated for these analyses. In both experiments, the overall distribution of up-regulated proteins among functional categories significantly differed between egg quality types (χ^2^, *p*<0.0001). Double asterisks indicate protein categories whose proportional representation differed significantly between egg quality types (χ^2^, *p*≤0.05) after Benjamini Hochberg correction for multiple post-hoc tests. Single asterisks indicate other protein categories with *p*-values from the post-hoc χ^2^ analyses ≤0.05 before Benjamini Hochberg correction. Ensembl Protein IDs and associated gene, transcript and protein names, functional categories, type of regulation (unique or up-regulated), and fold-difference between egg quality types in common protein N-SC values are given in S1 Table (Pooled Samples Experiment) and S2 Table (Multiple Samples Experiment).

In the Pooled Samples Experiment, proteins extracted from eggs from 4 good quality spawns or from 4 poor quality spawns were pooled before extensive fractionation by SDS-PAGE (20 fractions) prior to LC-MS/MS (Fig 1). Of the 2535 proteins that were identified, 892 (35.2%) showed a ≥2-fold difference in normalized spectral counts (N-SC) between egg quality types (n = 545), or were unique to an egg quality type (good quality n = 136, poor quality n = 211), and for the purposes of this study were considered to be ‘differentially regulated’. The distribution of these differentially regulated proteins among 13 categories of physiological function chosen to represent most (≥90%) of these proteins significantly differed (χ^2^, *p*<0.0001) between egg quality types (Fig 2a). Frequencies of up-regulated proteins related to energy metabolism and protein synthesis were significantly higher in good quality eggs, whereas frequencies of up-regulated zona pellucida proteins (ZPs) and lectins, as well as proteins related to endosome/lysosome function and oncogenes, were significantly higher in poor quality eggs.

**Fig 3:**
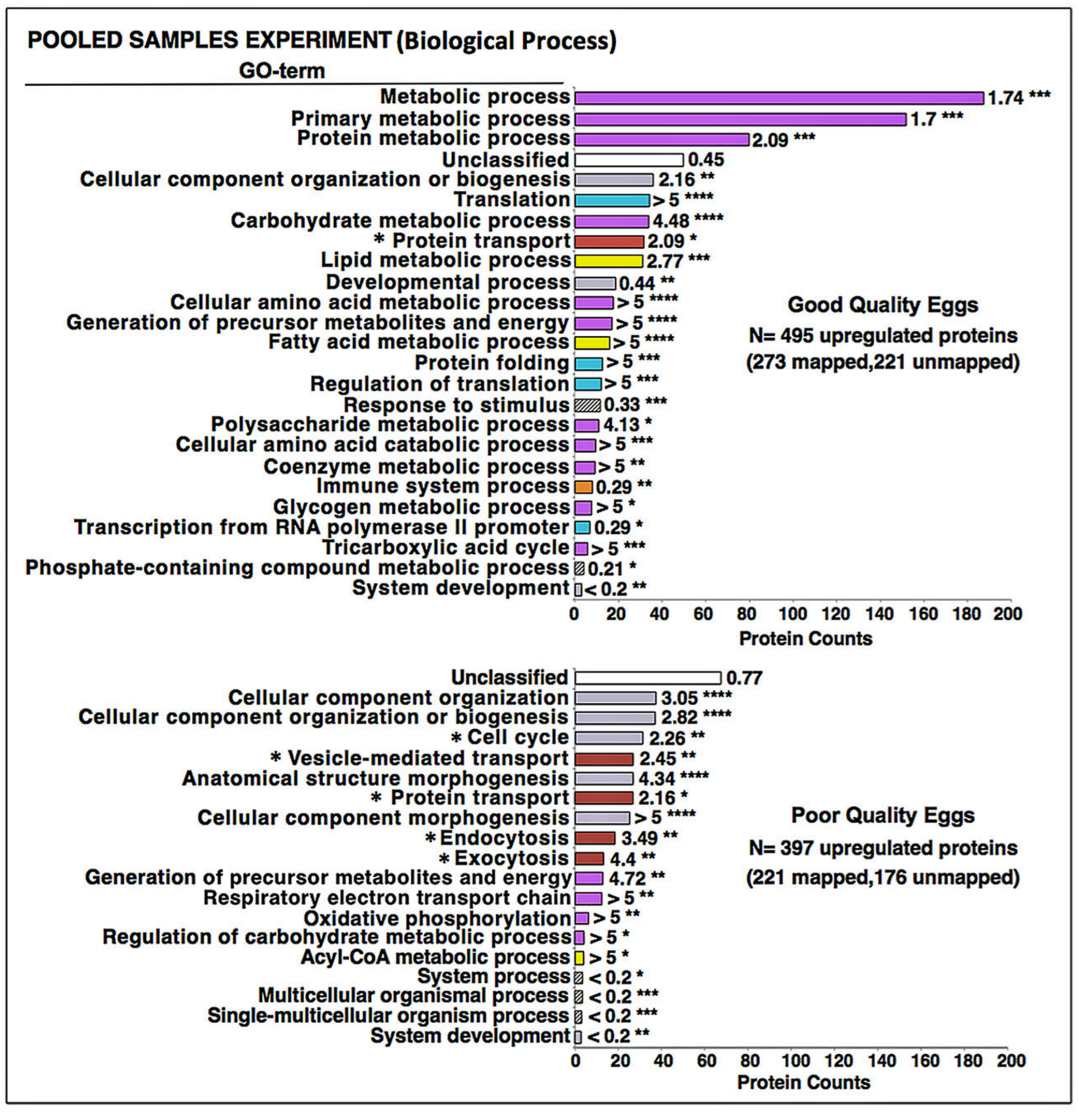
Enrichment of biological process gene ontology (GO) terms with proteins up-regulated in the Pooled Samples Experiment. Shown are the results of *Protein ANalysis THrough Evolutionary Relationships* (PANTHER) over-representation binomial tests [28] for enrichment of Biological Process GO terms with proteins up-regulated in good and poor quality zebrafish eggs in the Pooled Samples Experiment. **Top Panel**. Good quality eggs. **Bottom Panel**. Poor quality eggs. Horizontal bars indicate the number of proteins attributed to each GO term for which statistically significant results (*p*≤0.05 after Bonferroni correction for multiple tests) were observed. Numbers next to the bars indicate the fold-enrichment with proteins attributed to each term and the number of asterisks indicates the significance level of the enrichment, as follows *p*≤0.05 (*), *p*≤0.01 (**), *p*≤0.001 (***), and *p*≤0.0001 (****). Where possible, horizontal bars are colored to indicate corresponding protein functional categories shown in Fig 2; energy metabolism (magenta), cell cycle, division, growth and fate (lavender), protein synthesis (light blue), endosome/lysosome-related activities (brown), lipid metabolism (yellow), and immune system-related activities (orange). For poor quality eggs, GO terms preceded by an asterisk involve cytoskeletal activities. See text for details.

An automated *Protein ANalysis THrough Evolutionary Relationships* (PANTHER) over-representation test of the 892 differentially regulated proteins revealed that mostly gene ontology (GO) Biological Process terms related to energy metabolism, including lipid metabolism, and to protein synthesis were preferentially enriched with proteins from good quality eggs (Fig 3). The corresponding GO Molecular Function, Protein Class, and Cellular Component terms enriched by these proteins were also consistent with a proteomic emphasis on energy metabolism and protein synthesis, as well as cell cycle-related activities (S1–S3 Figs). The GO Biological Process terms Cellular component organization, Cellular component organization or biogenesis, Cell cycle, Vesicle-mediated transport, Anatomical structure morphogenesis, Protein transport, Cellular component morphogenesis, Endocytosis, and Exocytosis were enriched with proteins up-regulated in poor quality eggs (Fig 3). The GO Molecular Function, Protein Class, and Cellular Component terms enriched with these proteins also indicated a proteome tailored to cytoskeletal activities such as vesicle-mediated transport, endocytosis and exocytosis, as well as activities relevant to the cell cycle (e.g. karyokinesis, cytokinesis) (S1–S3 Figs).

**Fig 4:**
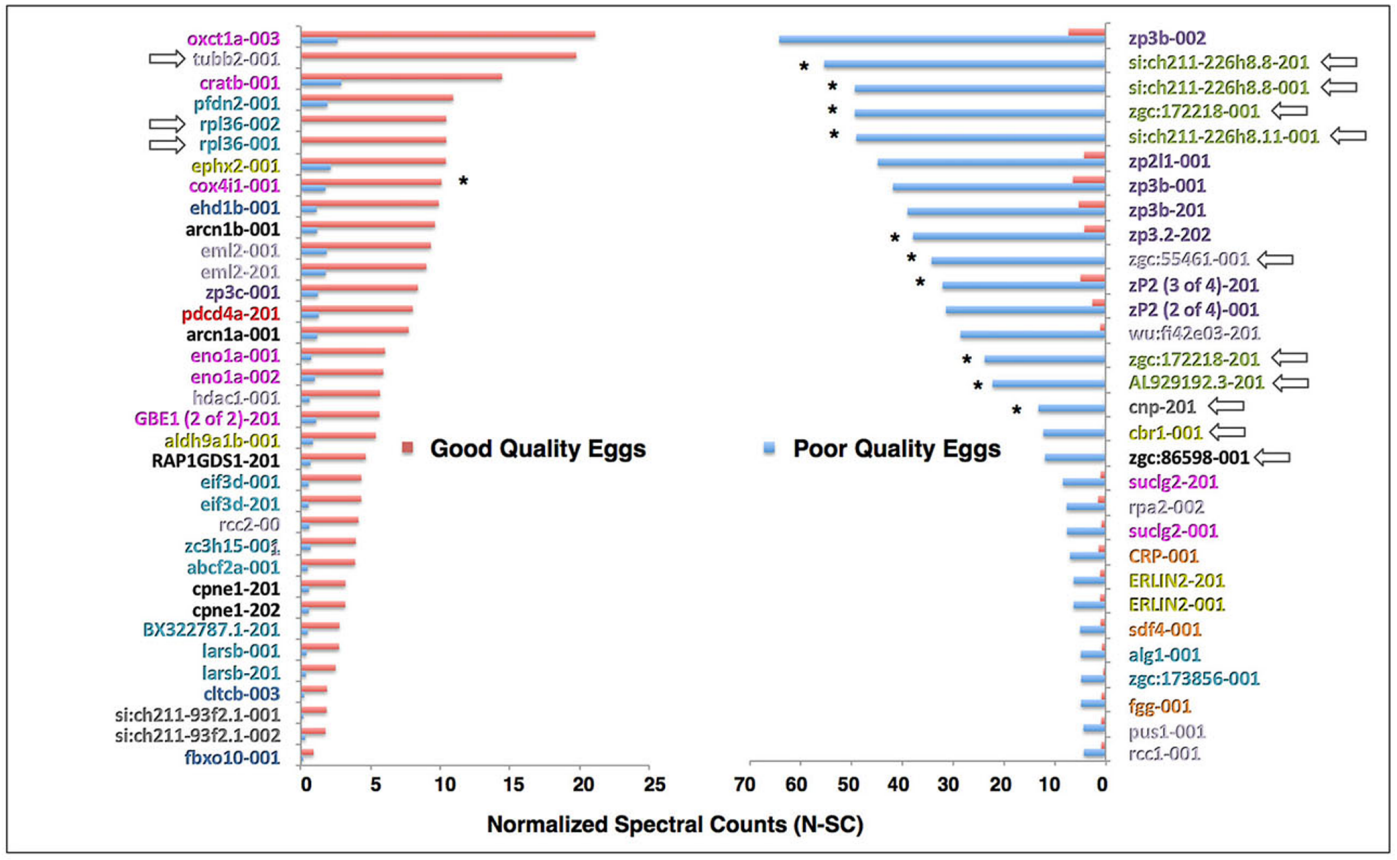
Proteins highly up-regulated in good or poor quality eggs in the Pooled Samples Experiment. The proteins are named for the transcript(s) to which spectra were mapped; for full protein names see S1 Table. These proteins were either unique to an egg quality type or they showed a ≥5-fold difference in N-SC between egg quality types, placing them in the top 2.3% of proteins showing such differences. **Left Panel**. Proteins highly up-regulated in good quality eggs. **Right Panel**. Proteins highly up-regulated in poor quality eggs. Where possible, protein (transcript) labels are color-coded to indicate functional categories to which the proteins were attributed (Fig 2). Arrows indicate proteins unique to an egg quality type. Asterisks indicate proteins detected and regulated in the same direction in the Multiple Samples Experiment.

Among proteins with a ≥5-fold change in N-SC between egg quality types, or unique to an egg quality type (Fig 4), which we considered to be “highly up-regulated”, those highly up-regulated in good quality eggs (n = 35) were mainly related to protein synthesis (28.6%), energy metabolism (17.1%) and lipid metabolism (5.7%), and cell cycle, growth and fate regulation (20%), with the remaining categorized proteins being related to protein degradation and synthesis inhibition (8.6%) and apoptosis (2.9%). In this group, only two ribosomal proteins (rpl36-001, rpl36-002) and one tubulin (tubb2-001) were unique to good quality eggs. Corresponding proteins highly up-regulated in poor quality eggs (n = 30) were mainly ZPs (23.3%) and lectin family members (20.0%), as well as proteins related to cell cycle, growth and fate regulation (16.7%), with the remaining categorized proteins being related to immune function (10.0%), energy metabolism (6.7%), lipid metabolism (6.7%) and protein synthesis (6.7%). Aside from the lectins, only 4 of these proteins were unique to poor quality eggs including a tubulin (zgc:55461–001), carbonyl reductase (cbr1-001), casein kinase (zgc:86598–001) and 2’, 3’-cyclic nucleotide 3’ phosphodiesterase (cnp-201).

In the Multiple Samples Experiment, where protein extracts of eggs from 4 good quality spawns or from 4 poor quality spawns were individually subjected to limited fractionation by SDS-PAGE (2 fractions excluding the major yolk proteins) prior to LC-MS/MS (Fig 1), the 369 proteins identified in the High Molecular Weight (HMW) fraction and 438 proteins identified in the Low Molecular Weight (LMW) fraction were combined to analyze their distribution among functional categories and for enrichment analyses; however, they were separately submitted to statistical analyses performed to detect significant differences in abundance of individual proteins between egg quality groups.

Considering only proteins with a ≥2-fold difference in average N-SC between egg quality groups, or proteins unique to an egg quality group, 123 proteins were up-regulated in good quality eggs and 151 proteins were up-regulated in poor quality eggs in the Multiple Samples Experiment. The distribution of these differentially expressed proteins among functional categories significantly differed between egg quality groups (χ^2^, *p*<0.0001), with frequencies of up-regulated proteins related to energy metabolism and lipid metabolism, and the incidence of vitellogenin (Vtg) products, being significantly higher in good quality eggs, and the frequency of up-regulated ZPs being significantly higher in poor quality eggs (Fig 2). Mapping of the Vtg-products up-regulated in good quality eggs to their parent yolk protein domains in zebrafish Vtgs revealed almost all to be derivatives of the major yolk protein, lipovitellin (arrows in Fig 1).

**Fig 5:**
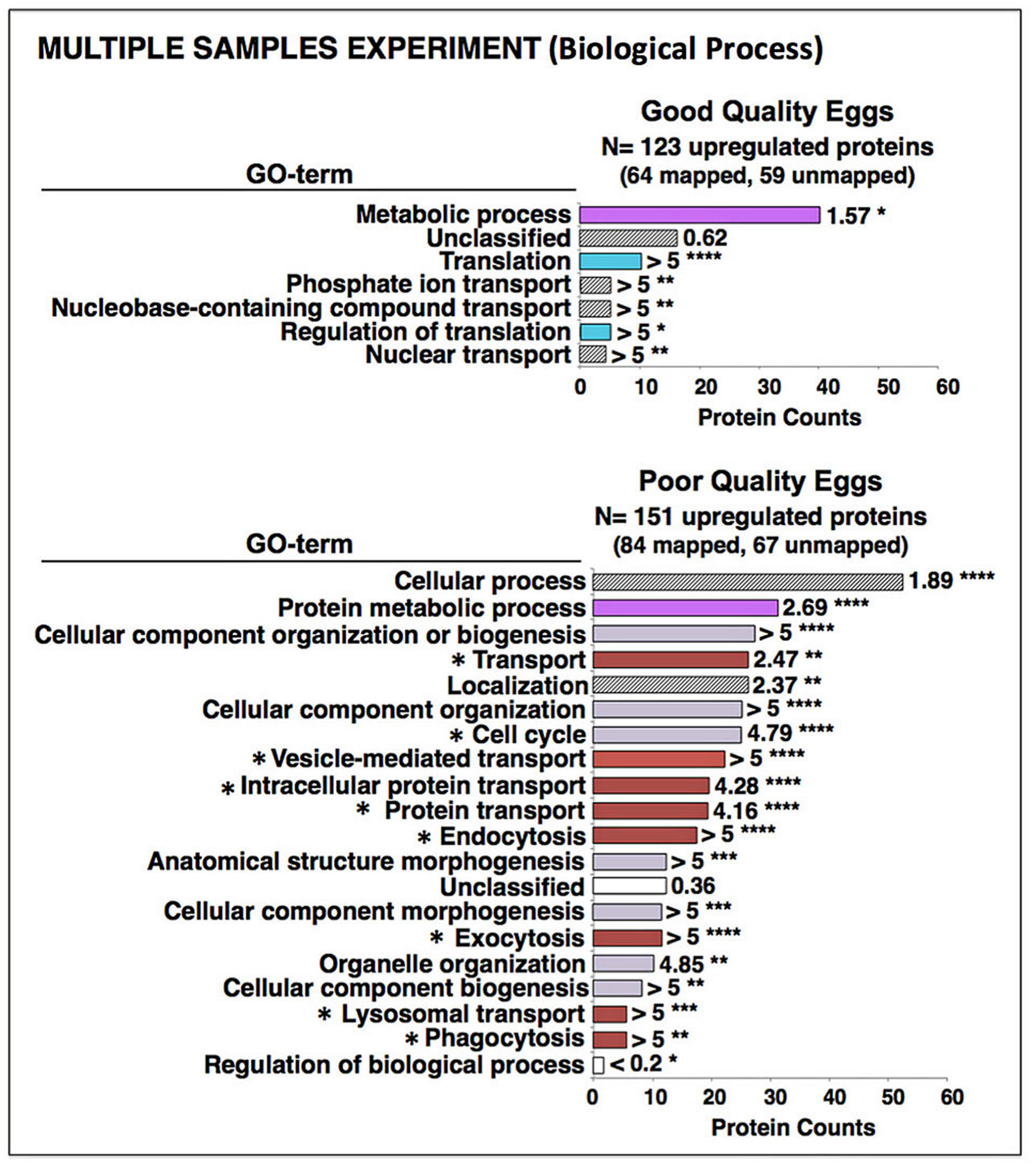
Enrichment of biological process gene ontology (GO) terms with proteins up-regulated in the Multiple Samples Experiment. Shown are the results of PANTHER over-representation tests for enrichment of Biological Process GO terms with proteins up-regulated in good and poor quality zebrafish eggs in the Multiple Samples Experiment. **Top Panel**. Good quality eggs. **Bottom Panel**. Poor quality eggs. Horizontal bars indicate the number of proteins attributed to each GO term for which statistically significant results (χ^2^, *p*<0.05) were observed. Numbers next to the bars indicate the fold-enrichment with proteins attributed to each term and the number of asterisks indicates the significance level of the enrichment, as follows *p*≤0.05 (*), *p*≤0.01 (**), *p*≤0.001 (***), and *p*≤0.0001 (****). Where possible, horizontal bars are colored to indicate corresponding protein functional categories shown in Fig 2; energy metabolism (magenta), protein synthesis (light blue), cell cycle, division, growth and fate (lavender), endosome/lysosome-related activities (brown). For poor quality eggs, GO terms preceded by an asterisk involve cytoskeletal activities. See text for details.

The PANTHER enrichment analyses revealed that GO Biological Process and Molecular Function terms relevant to metabolism and protein synthesis were enriched with proteins up-regulated in good quality eggs (Fig 5 and S1 Fig). The GO Protein Class and Cellular Component terms enriched with these proteins were also consistent with a proteomic emphasis on energy metabolism and protein synthesis (S2 and S3 Figs). Proteins up-regulated in poor quality eggs mainly enriched the GO Biological Process terms—Cellular process, Protein metabolic process, Cellular component organization or biogenesis, Transport, Localization, Cell cycle, Vesicle mediated transport, Protein- and Intracellular protein-transport, and Endocytosis (Fig 5). The GO Molecular Function, Protein Class and Cellular Component terms enriched by these proteins (S1–S3 Figs) were also in agreement with a proteome tailored to cytoskeletal activities such as vesicle-mediated transport, phagocytosis, endocytosis and exocytosis, and related activities relevant to the cell cycle.

**Fig 6:**
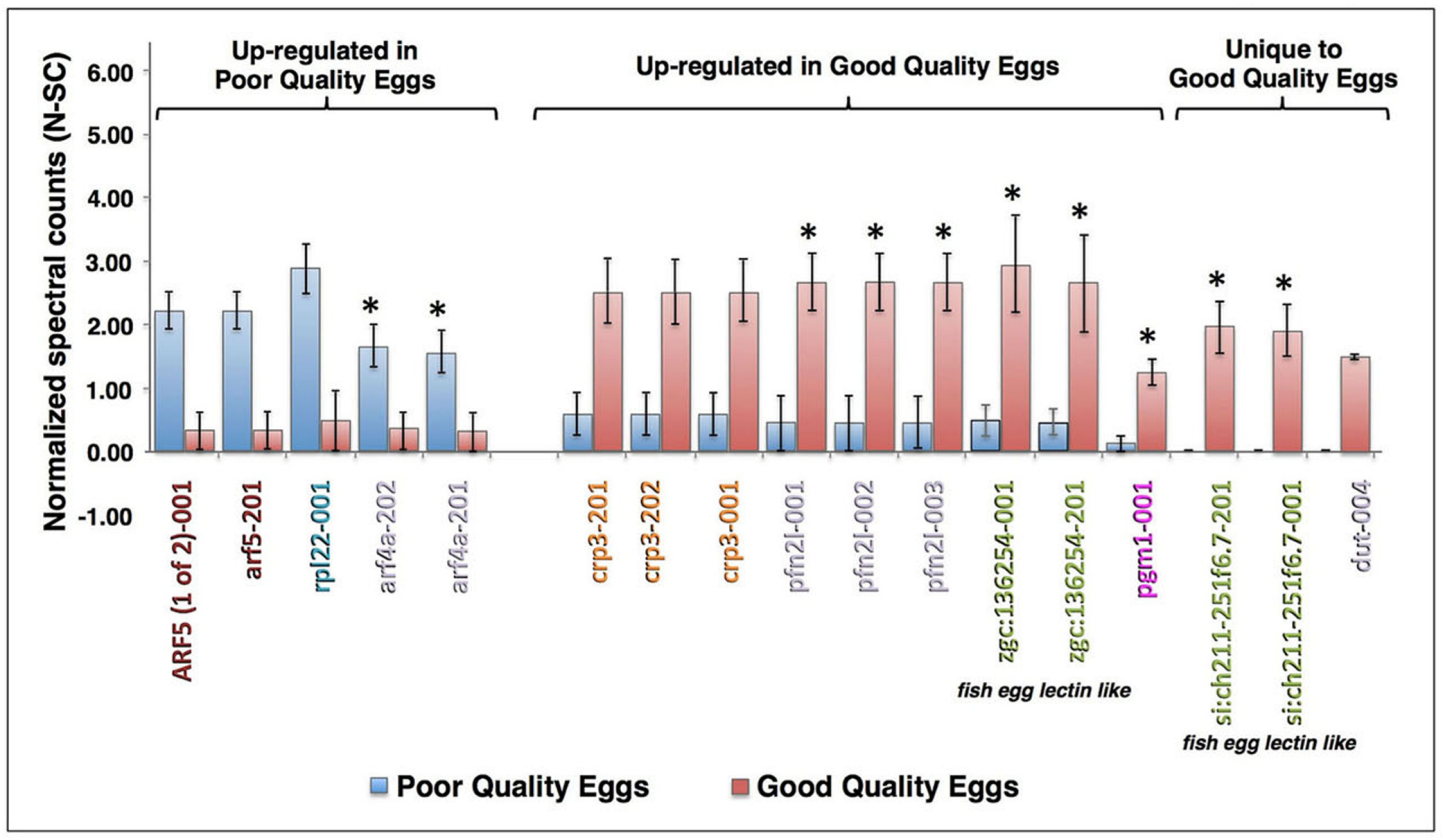
Proteins significantly differing in abundance between egg quality types in the Multiple Samples Experiment. The proteins are named for the transcript(s) to which spectra were mapped; for full protein names, see S2 Table. Only proteins common to both egg quality groups with ≥4-fold difference in N-SC between groups, placing them in the top 2.3% of proteins showing such differences, or proteins unique to an egg quality group with mean N-SC values ≥0.5 and a statistically significant difference in N-SC between egg quality groups, are shown (independent samples t-test, *p*≤0.05 after Benjamini Hochberg correction for multiple tests). Vertical bars indicate mean N-SC values (n = 4 per egg quality type) and vertical brackets indicate SEM. Protein (transcript) labels are color-coded to indicate functional categories to which the proteins were attributed (Fig 2). Asterisks indicate proteins that were also detected and regulated in the same direction in the Pooled Samples Experiment.

In the Multiple Samples Experiment, 17 proteins displayed a statistically significant difference in N-SC values between egg quality groups (Fig 6). Four forms of ADP-rybosylation factor (Arf) and one form of ribosomal protein L22 (Rpl22) were significantly up-regulated in poor quality eggs. Significantly up-regulated in good quality eggs were three isoforms of c-reactive protein 3 (Crp3), three isoforms of profilin-family member 2-like (Pfn2l), four fish egg lectin-like proteins, one form of phosphoglucomutase 1 (Pgm1), and a variant of deoxyuridine 5'-triphosphate nucleotide-hydrolase (Dut-004). Two of the lectin-like proteins and Dut were considered to be unique to good quality eggs because their mean N-SC values in poor quality eggs did not significantly differ from zero (Fig 6). Discounting isoforms, five proteins significantly up-regulated in good or poor quality eggs in the Replicated Samples Experiment were also up-regulated in the same direction in the Pooled Samples Experiment (i.e. Arf4a, Pfn2l, Zgc:136254, Pgm1, and Si:ch211-251f6.7). Spawns from the present experiment, which represented extremes of egg quality, could be sorted into egg quality groups with 100% accuracy based upon the sum of N-SC values for the four proteins that were up-regulated in good quality eggs both experiments (Σ Pfn2l N-SC + Σ Zgc:136254 N-SC + Pgm1 N-SC + Σ Si:ch211-251f6.7 N-SC); the mean of these summed N-SC values differed between egg quality groups by nearly an order of magnitude (poor quality 2.33±1.59, good quality 18.67±2.63) with no overlap between groups in range (poor quality 0–6.95, good quality 14.72–26.39) or 95% confidence interval (poor quality -2.73–7.39, good quality 10.32–27.03).

When proteins up-regulated in good or poor quality eggs in either experiment were submitted to PANTHER Pathways enrichment analyses, they were found to be significantly overrepresented in 14 different biological pathways (S3 Table). Proteins up-regulated in good quality eggs were overrepresented in three pathways: Pyruvate metabolism and *De novo* purine biosynthesis (Pooled Samples Experiment), and 5-hydroxytryptamine degradation (Multiple Samples Experiment). Proteins up-regulated in poor quality eggs were overrepresented in the remaining 11 pathways, 9 of which were significantly enriched in both experiments (S3 Table). Remarkably, when submitted proteins were individually mapped to all 14 pathways (S4 Fig), it was discovered that only 74 non-redundant proteins could account for all pathway enrichment.

**Fig 7:**
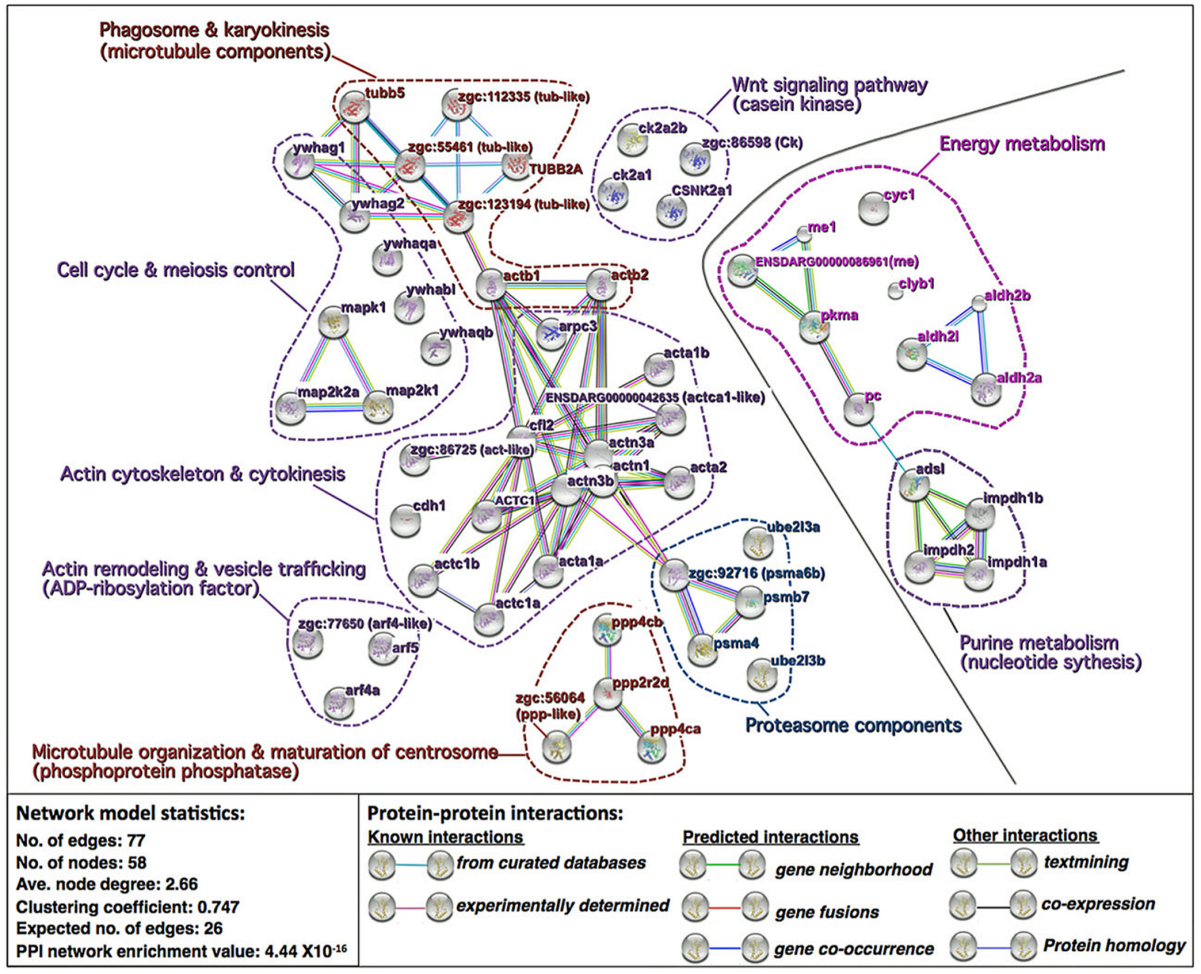
Network of differentially expressed proteins overexpressed in specific biological pathways. Shown are the results of *Search Tool for the Retrieval of Interacting Genes/Proteins* (STRING) network analysis of the 74 non-redundant proteins that were differentially regulated in good versus poor quality eggs and were over-represented in specific biological pathways in the PANTHER Pathways enrichment analyses (S3 Table). Proteins up-regulated in good quality eggs are shown to the right of the solid grey line and proteins up-regulated in poor quality eggs are shown to the left of the line. Each network node (sphere) represents all proteins produced by a single, protein-coding gene locus (splice isoforms and post-translational modifications collapsed). Only nodes representing query proteins are shown. Nodes are named for the transcript(s) to which spectra were mapped, with the text colored according to the processes shown in Fig 2; for full protein names, see S1 and S2 Tables. Small nodes represent proteins of unknown 3D structure. Large nodes represent proteins for which some 3D structure is known or predicted. Edges (lines) represent protein-protein associations meant to be specific and meaningful, i.e. proteins jointly contribute to a shared function but do not necessarily physically interact. Model statistics are presented at the lower left. Explanation of edge colors is given on the lower right. Dashed lines encircle groups of transcripts involved in the named biochemical pathways or physiological processes. See text for details.

Network analysis using the *Search Tool for the Retrieval of Interacting Genes/Proteins* (STRING) and the zebrafish protein database resolved the 74 ‘pathway’ proteins into a network with a significantly greater number of known and predicted interactions between proteins than would be expected of a list of 74 proteins randomly chosen from the zebrafish database (PPI network enrichment value P≤.44 x10^-16^) (Fig 7). Proteins up-regulated in good quality eggs formed a subnetwork made up of two protein clusters, one involved in energy metabolism and containing two forms of malic enzyme (Me), pyruvate kinase (Pkma), pyruvate carboxylase (Pc), cytochrome c-1 (Cyc1), citrate lyase (Cly), and three forms of aldehyde dehydrogenase (Aldh), and the other involved in purine (nucleotide) biosynthesis and containing adenylosuccinate lyase (Adsl) and three forms of inosine monophosphate dehydrogenase (Impdh) (Fig 7, right side). Proteins up-regulated in poor quality eggs formed a subnetwork made up of 7 clusters, two of which are made up of proteasome components, including three 20S proteasome subunits (Psm) and 2 forms of ubiquitin-conjugating enzyme E2 (Ube2), or Wnt signaling pathway components, including 4 forms of casein kinase (Csk, Csnk) (Fig 7, left side). The remaining 5 clusters are directly or indirectly involved in cytoskeletal functioning including cell cycle activities and regulation of mitosis and meiosis, vesicle trafficking, and phagocytosis. These include clusters made up of 4 forms of phophoprotein phosphatase (Ppp), or 3 forms of ADP-ribosylation factor (Arf), a cluster of cell cycle- and meiosis-regulating proteins including mitogen activated protein kinase (Mapk), two forms of Mapk kinase (Map2K), and 5 forms of 14-3-3 (Ywha) protein (i.e. 3-monooxygenase/ tryptophan 5-monooxygenase activation protein), a cluster of microtubule-related components including 5 forms of tubulin (Tub) and 2 forms of beta actin (Actb), and a large cluster consisting of actin cytoskeletal components including 8 forms of actin or actin-like protein, 3 forms of actinin (Actn), actin-related protein 2/3 complex subunit 2 (Arpc2), cadherin 1 (Cdh1) and cofilin 2 (Cfl2).

A STRING enrichment analysis of these 74 ‘pathway proteins’ included Kyoto Encyclopedia of Genes and Genomes (KEGG) Pathways, Protein Families Database (PFAM) Protein Domains, and Interpro Protein Families Database (INTERPRO) Protein Domains and Features (Table 2). Proteins in the good quality egg subnetwork significantly enriched pathways for Pyruvate metabolism, Purine metabolism and Metabolic pathways (KEGG), and also enriched Aldehyde dehydrogenase family domains, IMP dehydrogenase/GMP reductase domain, and Malic enzyme N-terminal and NAD-binding domains (PFAM). Proteins in the poor quality egg subnetwork significantly enriched the pathways Regulation of actin cytoskeleton, Adherins junction, Tight junction, Gap junction, Focal adhesion, Phagosome, and Cell cycle and Oocyte meiosis, among others (KEGG), and also enriched protein domains and features including Actin, Actin and Actin-like conserved site, Actin family, Spectrin repeat, Tubulin GTPase domain and C-terminal domain, 14-3-3 (ywha) protein, conserved site, and domain, Serine/threonine dual specificity protein kinase (e.g. Map2K) catalytic domain, and Adp-ribosylation factor family, among others (PFAM and INTERPRO) (Table 2).

**Table 2:** Enrichment analysis of differentially expressed proteins overexpressed in specific biological pathways.

**Kegg Pathways Enriched in Network**			
**Pathway ID**	**Pathway description**	**Protein count**	**FDR**
**4520**	**Adherins junction**	**10**	**1.80E-12**
**4530**	**Tight junction**	**10**	**2.06E-10**
**4810**	**Regulation of actin cytoskeleton**	**10**	**1.13E-08**
**4114**	**Oocyte meiosis**	**8**	**1.38E-08**
**4540**	**Gap junction**	**7**	**2.28E-07**
**4145**	**Phagosome**	**6**	**1.84E-05**
**4510**	**Focal adhesion**	**7**	**2.23E-05**
***620***	***Pyruvate metabolism***	***4***	***4*.*66E-05***
**4110**	**Cell cycle**	**5**	**0.000252**
***230***	***Purine metabolism***	***5***	***0*.*000714***
**5132**	**Salmonella infection**	**4**	**0.000714**
**4270**	**Vascular smooth muscle contraction**	**4**	**0.00241**
**1120**	**Microbial metabolism in diverse environments**	**4**	**0.00492**
**4261**	**Adrenergic signaling in cardiomyocytes**	**4**	**0.00598**
**1100**	**Metabolic pathways**	**9**	**0.00901**
**PFAM Protein Domains Enriched in Network**			
**Pathway ID**	**Domain description**	**Protein count**	**FDR**
**PF00022**	**Actin**	**10**	**6.07E-18**
**PF00244**	**14-3-3 protein**	**5**	**2.41E-08**
**PF00091**	**Tubulin/FtsZ family, GTPase domain**	**4**	**0.000166**
**PF03953**	**Tubulin C-terminal domain**	**4**	**0.000166**
**PF08726**	**Ca2+ insensitive EF hand**	**3**	**0.000177**
**PF00171**	***Aldehyde dehydrogenase family***	**3**	**0.007**
**PF00227**	**Proteasome subunit**	**3**	**0.0124**
**PF00478**	***IMP dehydrogenase / GMP reductase domain***	**3**	**0.0155**
**PF00390**	***Malic enzyme*, *N-terminal domain***	**2**	**0.0188**
**PF00435**	**Spectrin repeat**	**3**	**0.0188**
**PF03949**	***Malic enzyme*, *NAD binding domain***	**2**	**0.0188**
**PF00025**	**ADP-ribosylation factor family**	**3**	**0.04**
**INTERPRO Protein Domains and Features Enriched in Network**			
**Pathway ID**	**Domain or feature description**	**Protein count**	**FDR**
**IPR004001**	**Actin, conserved site**	**10**	**1.18E-20**
**IPR020902**	**Actin/actin-like conserved site**	**10**	**4.69E-20**
**IPR004000**	**Actin family**	**10**	**4.41E-18**
**IPR000308**	**14-3-3 protein**	**5**	**1.75E-08**
**IPR023409**	**14-3-3 protein, conserved site**	**5**	**1.75E-08**
**IPR023410**	**14-3-3 domain**	**5**	**1.75E-08**
**IPR002290**	**Ser/thr/dual spec. protein kinase, cat. domain**	**7**	**0.00219**

Results of a STRING enrichment analysis of the protein network shown in Fig 7 for proteins that were differentially regulated in good versus poor quality eggs and were over-represented in specific biological pathways in the PANTHER Pathways enrichment analyses (S3 Table). Shown are KEGG pathways and INTERPRO protein domains represented by ≥4 or ≥5 genes, respectively, with a false discovery rate (FDR) <0.01, and PFAM Protein Domains represented by ≥2 genes, with a FDR <0.05. Entries for proteins only found in good quality eggs are set in italics type.

## Discussion

The present study revealed the disparate proteomic profiles of good and poor quality zebrafish eggs in two separate experiments with different levels of sample replication and fractionation before mass spectrometry. In spite of the methodological differences, both experiments demonstrated that good and poor quality zebrafish eggs have distinct proteomic profiles. The percentage of up-regulated proteins related to energy metabolism was significantly greater in good quality eggs in both experiments (Fig 2). In the higher resolution Pooled Samples Experiment, the percentage of up-regulated proteins related to protein synthesis was also significantly higher in good quality eggs (Fig 2). For both experiments, the GO-Biological Process enrichment analysis attributed a far greater proportion of up-regulated proteins to terms relevant to energy metabolism (e.g. Metabolic process, Primary metabolic process, Protein metabolic process, Carbohydrate metabolic process, others) and protein synthesis (e.g. Translation, Regulation of translation, Protein folding, others) for good quality eggs than for poor quality eggs (Figs 3 and 5), as did the GO-Molecular Function enrichment analysis (S1 Fig), and the GO-Protein Class enrichment analysis (S2 Fig). Also in both experiments, the percentage of up-regulated proteins related to lipid metabolism was higher in good quality eggs, significantly so in the Multiple Samples Experiment where such proteins were not detected in poor quality eggs (Fig 2). Collectively, these findings suggest some failure of poor quality eggs to maintain regular cellular activities involving energy metabolism, lipid metabolism and protein synthesis relative to normally functioning eggs with high embryo developmental potential.

In the Multiple Samples Experiment, polypeptide derivatives of the Vtg-derived major yolk protein, lipovitellin (Lv), were strongly up-regulated in good quality eggs relative to poor quality eggs (Fig 2). These derivatives were mainly proteolytic products of the intact Lv heavy chain (LvHc) and Lv light chain (LvLc), which we deliberately excluded from the HMW and LMW fractions to better resolve smaller and less abundant proteins (see Fig 1). In the Pooled Samples Experiment, contribution of these small Lv derivatives to overall Vtg N-SC values would have been masked by the dominant contribution of proteins in the major Lv bands. Evidence for a dearth of Lv proteolysis products in poor quality eggs was also obtained when proteomic profiles of eggs from Eurasian perch (*Perca fluviatilis*) of varying reproductive performance were revealed by 2D-DIGE followed by MS/MS by Castets et al. [19]. These authors found ~42 kDa Vtg products mapping to *Perca flavescens* Vtg C, which contains only Lv domains, in three spots characterized by low expression in poor quality eggs. Proteolysis of Lvs normally occurs during final oocyte maturation in zebrafish and possibly all teleosts [29] as a mechanism for generation of free amino acids (FAA), which act as osmolytes to promote oocyte hydration and which are a critical energy source for early embryos. Most energy metabolism in early embryos is based on utilization of these FAA whose carbon skeletons are fed into the tricarboxylic acid cycle to fuel intermediary metabolism [30]. This cycle may be more active in high quality ova as the pyruvate metabolism pathway was overrepresented by proteins up-regulated in good quality eggs (S3 Table, see also S4 Fig **Panel l**). FAA are also utilized to support gluconeogenesis; glucose is an important substrate for synthesis of nucleic acids and polysaccharides in embryonic development [6]. Thus, the apparent deficiency of proteolytic products of Vtgs in poor quality zebrafish eggs may indicate a failure of the oocytes to properly transition from oocyte growth into final maturation antecedent to spawning, which would compromise water balance, energy metabolism and anabolic activities in any developing embryos.

Impairment of a proper transition of oocytes into final maturation is also suggested by the greater representation of up-regulated proteins related to endosome/lysosome activity in poor quality eggs in both experiments, which was significantly greater than seen in good quality eggs in the Pooled Samples Experiment (Fig 2). In both experiments, the GO-Biological Process enrichment analysis attributed a high proportion of up-regulated proteins to terms relevant to endosome/lysosome activity (e.g. Vesicle-mediated transport, Endocytosis, Exocytosis, Phagocytosis, Lysosomal transport) for poor quality eggs but not good quality eggs (Figs 3 and 5), as did the GO-Molecular Function, Protein Class, and Cellular Component enrichment analyses (S1–S3 Figs). The network comprised of proteins up-regulated in poor quality eggs that were overrepresented among several biological pathways (S3 Table) is also dominated by cytoskeletal components and clusters of other proteins that regulate these components (Fig 7, see also Table 2). The predominant activities occurring in growing teleost oocytes are the massive uptake of Vtgs from the circulation via receptor-mediated endocytosis, fusion of the endosomes containing Vtgs with lysosomes containing cathepsin D (CtsD) to form multivesicular bodies, and cleavage of Vtgs by CtsD into their constituent yolk proteins, which are stored in yolk granules or globules until they undergo the maturational proteolysis associated with oocyte hydration [29, 30]. These bulk endocytotic activities are terminated at initiation of oocyte maturation with cessation of Vtg uptake [31] and deactivation of much of the extensive cellular endo-lysosomal machinery [32], events that may be disrupted in poor quality zebrafish oocytes/eggs. Indirect evidence supporting this concept comes from results of the PANTHER pathways enrichment analysis showing that the 5-hydroxytryptamine (serotonin) degradation pathway was significantly enriched in good quality eggs versus poor quality eggs (S3 Table), due to overrepresentation with several aldehyde dehydrogenases (S4 Fig **Panel n**). Serotonin is a potent reversible inhibitor of steroid-mediated resumption of meiosis and final oocyte maturation [33] and its degradation in good quality eggs would promote the transition to final maturation; with the process being inhibited or dysregulated in poor quality eggs with deficient serotonin degradation. Enrichment of the epidermal growth factor (EGF) receptor signaling pathway with several key proteins in poor quality eggs (S3 Table, see also S4 Fig **Panel g**) provides further opportunity for dysregulation of oocyte maturation, which is mediated by luteinizing hormone (LH). EGF is expressed in the zebrafish oocyte and its receptor (EGFR) is expressed in the follicle cell layer; paracrine actions of EGF in zebrafish include down-regulation of the LH receptor and of follicle responsiveness to LH, and upregulation of follicle stimulating hormone (FSH) receptor [34]. In the present study, the exact same suite of proteins enriched the fibroblast growth factor (FGF) signaling pathway; however, little is known about ovarian actions of FGF in zebrafish or other teleosts.

An alternative, but not mutually exclusive, explanation for the enrichment of poor quality eggs with proteins relevant to endo-lysosomal processes could be a higher level of autophagocytosis (autophagy), a normal process in oocytes/eggs and embryos of fishes, including zebrafish [35]. The most common trigger of autophagy is nutrient restriction, which could result from limitation of FAA available to early embryos. Autophagy is normally a cytoprotective process that facilitates cell survival by ensuring adequate energy levels via recycling of damaged macromolecules and cellular components. However, dysregulation of autophagy can be cytotoxic, resulting in the accumulation of abnormal proteins and/or damaged organelles that is commonly observed in human neurodegenerative diseases, such as Alzheimer’s, Huntington’s, and Parkinson’s diseases [36]. Notably, pathways for all three of these diseases were enriched with proteins up-regulated in poor quality eggs in the present study (S3 Table). While we did not detect any definitive protein markers of autophagy signaling in poor quality eggs, the process shares much of the same cellular protein machinery involved in other endo-lysosomal and vesicle trafficking activities that appear to be up-regulated in poor quality eggs, including the majority of proteins in the network shown in Fig 7. In zebrafish as in other vertebrates, autophagy opposes and is intimately linked to apoptosis via proteins playing a regulatory role in both events [37], and poor quality eggs exhibited a greater proportion of up-regulated proteins related to apoptosis in both experiments, significantly so in the Pooled Samples Experiment (Fig 2). Apoptosis is programmed cell death promoted by processes leading to mitochondrial dysfunction (loss of membrane potential) and downstream activation of the caspase pathway. Some examples of apoptosis-related proteins upregulated in or unique to poor quality eggs in this study include several variants of caspase 3a (Casp3a) and mitogen-activated protein kinase 1 (MapK1) (S1 Table), and also death-associated protein 1b (Dap1b) (S2 Table). The increased representation of up-regulated oncogene-related proteins in poor quality eggs may also be related to a struggle to offset apoptosis. This increase was almost entirely due to up regulation of multiple variants of tumor protein D52-like (S1 Table); D52 is known to inhibit apoptosis and promote cell proliferation in human cancer cells [38]. Thus, the up-regulation of endo-lysosome-, autophagy-, apoptosis- and oncogene-related proteins in poor quality eggs could be functionally (or dysfunctionally) interrelated.

Wnt signaling, which plays critical roles in cell proliferation and fate determination, axis patterning and morphogenetic movements [39], probably also influences egg quality in zebrafish. Canonical Wnt signaling is mediated by intracellular free β-catenin, normally kept at low levels via its tethering by cadherin to the inner surface of the plasma membrane, or phosphorylation on its N-terminus by Ck1 and glycogen synthase kinase-3β, targeting it for ubiquitination and proteasome-mediated degradation [40]. Casein kinase 2 (Ck2) acts at several levels in these pathways to promote Wnt signaling and rescue β-catenin from destruction. Non-canonical Wnt signaling, which is independent of β-catenin, affects release of intracellular Ca^2+^, activates JNK signaling and the Rho family of small GTPases, and regulates cadherin recycling, signals ultimately affecting cytoskeletal architecture, the establishment and modulation of cell polarity, and cell movements [41]. In the present study, the presence of Ck2 and proteasome component (Psm and Ube) clusters among the network of ‘pathway proteins’ up-regulated in poor quality zebrafish eggs (Fig 7), coupled with enrichment of Wnt signaling, Cadherin signaling, and Cytoskeletal regulation by Rho GTPase pathways by these proteins (S3 Table), implicates dysregulation of Wnt signaling in the etiology of poor egg quality in zebrafish.

Lectins were elevated in poor quality zebrafish eggs in both experiments, substantially so in the Pooled Samples Experiment (Fig 2). These were mainly sea urchin egg lectin (SUEL)-type and L-rhamnose-binding lectins (L-RBLs) including some fish egg lectin (FEL)-like proteins (70–75%), and also Ca2+-dependent (C-type) lectins (25–30%). C-type lectins have been localized to the cortical granules of fish eggs from whence they are discharged at fertilization into the perivitelline space, where they function in chorion hardening of the egg and establishing the block to polyspermy [42]. The other lectin types are known to play roles in innate immunity by binding to carbohydrate molecules on the surface of pathogens and enhancing their clearance via opsonization and phagocytosis [43]. In the Pooled Samples Experiment, 3 SUEL-type and one L-RBL were amongst the most highly up-regulated proteins in poor quality eggs (Fig 4). Conversely, 4 novel FEL-like proteins with predicted rhamnose-binding properties were significantly up-regulated in good quality eggs in the Multiple Samples Experiment (Fig 6). Although the exact relation of the different types of lectin to egg quality remains to be verified, disparate expression of these proteins in zebrafish eggs of different quality grades suggests that they may impact embryo development. Carp FEL shows broad binding specificity for Gram-positive and Gram-negative bacteria, and injection of purified native FEL into zebrafish embryos markedly promoted embryo resistance to pathogenic *Aeromonas hydrophila* [43], indicting that the FELs are immunocompetent to defend developing embryos/larvae from pathogenic attack.

Zona pellucida proteins with ≥2-fold differential expression between good and poor quality eggs and nominal intact molecular weights ranging from 12 to 93.5 kDa, and from 16.5 to 104.8 kDa were detected in fractions covering the 30–60 kDa range in SDS-PAGE in the Multiple Samples Experiment and the 25–55 kDa range in the Pooled Samples Experiment (Fig 1), respectively. The high abundance of ZPs among proteins up-regulated in poor quality eggs, and the detection of some HMW ZP (i.e. 93.5 and 104.8 kDa) products in LMW fractions, may indicate premature disintegration of the egg envelope. Other possibilities are that the crosslinking of ZPs by transglutaminase to form a robust chorion following fertilization [44] is somehow impaired, or that the synthesis and deposition in the egg envelope of high molecular weight ZPs is disturbed in poor quality oocytes/eggs. As the disparate types of ZPs have considerable promise as biomarkers of egg quality, these possibilities should be evaluated in future research.

In spite of limited replication, substantial variation in N-SC values between fish, and the need to correct false discovery rate for large numbers of tests, 17 differentially expressed proteins exhibited statistically significant differences in N-SC between egg quality groups in our Multiple Samples Experiment and are, therefore, potential biomarkers of egg quality (Fig 6). Up-regulated in poor quality eggs were four variants of Arf4a or 5; the two Arf4a proteins were also up-regulated in the Pooled Samples Experiment. These Arfs are small GTPases that regulate vesicle trafficking and actin skeletal dynamics [45], including modulation of cytoskeletal regulation by Rho GTPase, a pathway enriched by proteins up-regulated in poor quality eggs in both experiments (S3 Table, see also S4 Fig **Panel a**). Three of these Arfs also contribute to the molecular phenotype of poor quality eggs partially illustrated in Fig 7. The other protein significantly up-regulated in poor quality eggs was ribosomal protein L22 (Rpl22), which to date to has not been implicated in any specific developmental pathway, mechanism, or disease.

Significantly elevated in good quality eggs were 3 variants of Crp3, which is classically involved in recognition (opsinization) of pathogens and cell damage during the acute phase response of innate immunity. C-reactive protein also engages in complex, mutually-stimulating interactions with autophagy and it promotes proliferation and inhibits apoptosis of certain tumor cells [46]. We speculate that maternal Crp3 is also engaged in these latter functions in early zebrafish embryos. Three variants of Pfn2l were significantly up-regulated in good quality eggs. Profilins regulate cytoskeletal dynamics, including actin polymerization and its coordination with microtubule dynamics [47], and they are also involved in regulation of small GTPase signaling and vesicle trafficking [48]. The glucose metabolizing enzyme, Pgm1 was also significantly elevated in good quality eggs (Fig 6). The earliest stages of embryogenesis are dependent upon cytosolic glycogen as an energy source, later switching to free amino acids [30], and maternally supplied Pgm1 may assist in glycogen utilization during this time. There are multiple beneficial and long-lasting effects of hepatic Pgm1 expression in later stage rainbow trout embryos and larvae on their subsequent development, growth and maturation that probably arise from increased flux through glycolysis [49]. Thus, Pgm1 may be an especially promising egg quality marker.

In the present study, a spectral counting procedure was employed to assess the relative abundance of proteins by virtue of the applicability of this ‘label-free’ technique to multiple highly complex biological samples, and in consideration of the higher degree of quantitative proteome coverage and linear dynamic range expected using this approach versus common stable isotope labeling techniques, features that are advantageous when large and global protein changes are observed [50]. The high consistency of results between our Pooled and Multiple Samples experiments with respect to differences between egg quality groups in representation of proteins among different functional (and GO) categories and pathways, and our detection of statistically significant differences in abundance of numerous proteins (N = 17 including isoforms) between egg quality groups in the latter experiment, as well as the ability of selected protein abundances to accurately predict egg quality in the same fish, confirm the existence of proteomic profiles related to egg quality. Future investigations of the proteomics of egg quality may benefit from the additional employment of some form of absolute protein quantification using internal standards in order to confirm protein abundance assessments with increased accuracy [20, 50], albeit at the potential expense of some of the advantageous features of spectral counting procedures noted above.

A dearth of developing embryos at 2–3 hps, potentially resulting from low fertility, was a common but not universal manifestation of poor egg quality in both of our experiments (Table 1). Although actual insemination of ova was not empirically assessed, based on the enormous disparity in size between the male and female gametes, proteomic differences between good and poor quality eggs collected immediately after spawning were considered to be unrelated to paternal contribution. Our ability to accurately sort spawns representing extremes of egg quality into the ‘correct’ egg quality groups based on the relative abundances of a few proteins up-regulated in good quality eggs in both experiments also indicates that, under the present experimental conditions, variation in the fertility or reproductive performance of male zebrafish did not influence our egg quality assessments. Further investigations will be required to confirm whether eggs that fail to give rise to developing embryos are fertilized, perhaps by employing males of a transgenic line expressing a fusion of a histone variant to green fluorescent protein [51], or some other marker protein amenable to live imaging. Failure of eggs to be fertilized, and/or failure of fertilized eggs to undergo early cell cleavage, may also be hallmarks of poor egg quality, in which case it may be necessary to partition out male effects on fertility using classical ‘nested’ mating designs.

## Conclusions

Female zebrafish bred at weekly intervals are an opportune model to investigate molecular determinants of egg quality because they exhibit considerable variation in egg quality associated with clear changes in proteomic profiles. The poor quality zebrafish eggs are characterized by a proportional deficiency of proteins involved in protein synthesis, energy metabolism and lipid metabolism, and a dearth of proteolytic products of the major Lv yolk proteins, with a corresponding surfeit of proteins involved in endo-lysosomal activities, including autophagy, apoptosis and oncogenes. We propose that these differences are interrelated and arise from failure of oocytes giving rise to poor quality eggs to properly transit from growth through final maturation, limiting the liberation of critical energy substrates (FAA) from Lvs and, thus, necessitating autophagy and impairing the normal maturational attenuation of mass endocytotic processes, developments that collectively disrupt the normal composition and functions of the cytoskeleton. Results of pathway enrichment analyses and network modeling implicate dysregulation of the Wnt signaling pathway as a contributing factor in poor egg quality. Several novel egg quality marker proteins were identified for careful validation in future studies; these include lectins, egg envelope proteins (ZPs), and 9 additional proteins (excluding isoforms) exhibiting significant differences in relative abundance between egg quality types. Collectively, these observations indicate that the proteomic profiles of zebrafish eggs are strongly linked to, and possibly determine, egg quality.

## Methods

### Animal care, spawning and egg quality assessment

Zebrafish (*Danio rerio*) of the AB strain originally emanating from Tübingen (Germany) were obtained from our zebrafish facility (INRA UR1037 LPGP, Rennes, France) where they had been bred for 7–8 generations. The fish were ~1 year of age at the start of experiments and of average length ~5.0 cm and average weight ~1.4g. The zebrafish were housed under standard conditions of photoperiod (14 hours light and 10 hours dark) and temperature (28°C) in 10 L aquaria, and were fed three times a day *ad libidum* with a commercial diet (GEMMA, Skretting, Wincham, Northwich, UK). Females were bred at weekly intervals. The night before spawning, paired males and females bred from different parents were separated by an opaque divider in individual aquaria equipped with marbles at the bottom as the spawning substrate. The divider was removed in the morning, with the fish left undisturbed to spawn. Approximately sixty eggs per female were collected at the 1-cell stage immediately after spawning and incubated in 100 mm Petri dishes filled with embryo medium (17.1 mM NaCl, 0.4 mM KCl, 0.65 mM MgSO_4_, 0.27 mM CaCl_2_, 0.01 mg/L methylene blue) to assess egg quality based on embryo development. Prior to assessing developmental competence, groups of 40 eggs per spawn were removed, frozen in liquid nitrogen, and stored at -80°C until being used for proteomics analyses.

Incubated eggs/embryos were serially sampled for observation at the early blatstula (~256 cell) stage (~2–3 hps), at the shield to 75% epiboly stages (~8 hps), at the early pharyngula stage (~24 hps), and during the hatching period at 48 and 72 hps (long-pec to protruding-mouth stages) following standard developmental staging [52]. At the time of sampling, the number of surviving eggs/embryos was recorded, those not surviving were removed and, for samples taken at 8 hps and beyond, the number of abnormal embryos was recorded. For samples taken at ~2–3 hps, any incidence of a high proportion (>50%) of abnormal embryos showing asymmetric cell cleavage and or early developmental arrest was also noted.

Egg samples utilized in the present experiments were selected from among N = 136 total spawns for which the mean ± SEM percentage of eggs yielding well-formed embryos surviving to 24 hpf was 74.35 ± 2.35%. As noted in Results, spawns for which survival of normal embryos to 24 hps was >90% were considered to contain good quality eggs, with those having a survival rate of <30% considered to contain poor quality eggs. By these criteria, 55 of the 136 spawns (40.4%) contained good quality eggs and 15 spawns (11.0%) contained poor quality eggs. From this sample set, good and poor quality spawns were selected for our experiments at random, with the exception that contribution of individual male and female breeders was restricted to a single spawn in this study.

All experiments complied with French & European regulations ensuring ‘animal welfare' and that 'Animals will be held in the INRA UR1037 LPGP fish facility (DDCSPP approval # B35-238-6).’ Experimental protocols involving animals were approved by the Comité Rennais d'éthique pour l'expérimentation animale (CREEA).

### Experimental design

Two separate experiments with different levels of sample replication and fractionation of samples before LC-MS/MS were conducted to characterize the proteome of good versus poor quality zebrafish eggs and to discover potential markers of egg quality. In the Pooled Samples Experiment, intended to maximize resolution of both rare and abundant proteins, egg protein extracts from 4 spawns (40 eggs/spawn) of good quality or of poor quality were pooled separately and were subjected to extensive fractionation by SDS-PAGE (n = 20 fractions) prior to LC-MS/MS (Fig 1). The Multiple Samples Experiment, intended to detect differential expression of the more abundant proteins, involved collection of a HMW fraction (30-66kDa) and a LMW fraction (14–28 kDa) of proteins (exclusive of intact Lv subunits) after SDS-PAGE prior to submitting the excised fractions LC-MS/MS, the entire procedure being repeated for 8 samples arising from 4 spawns of each egg quality type (Fig 1).

### Protein extraction and SDS-PAGE

Samples were subjected to sonication in 20 mM, pH 7.4, HEPES Buffer containing 200 mM EDTA, 100 mM DTT, 200 mM 4-(2-aminoethyl)-benzenesulfonyl fluoride, and 2 mM trans epoxysuccinyl-L-leucylamido-(4-guanidino) butane, on ice. Soluble protein extracts were recovered after centrifugation (15 000 x g) at ~4°C for 30 minutes. The remaining pellet was treated with 30 mM Tris/8M Urea/ 4% CHAPS buffer, re-sonicated on ice, pooled with soluble protein extracts from the same sample and ultracentrifuged (105,000 X g) for 1h at ~4°C followed by supernatant recovery and determination of the protein concentration by Bradford Assay [53] (Bio-Rad, Marnes-la-Coquette, France). Samples of extracts were then mixed with sample buffer (NuPAGE^®^ LDS Sample Buffer 4x) and DTT (NuPAGE^®^ Sample Reducing Agent) and incubated at 70°C for 10 min before being subjected to SDS-PAGE (80 μg protein/sample lane) on a 4–12% Bis-Tris precast gel (NuPAGE^™^ Novex^™^ 4–12% Bis-Tris Protein Gels) run in MOPS buffer (NuPAGE^®^ MOPS SDS Running Buffer) mixed with antioxidant (NuPAGE^®^ Antioxidant) at 200V-400mA (~23W) for 1h. After electropohoresis, gels were briefly rinsed in MilliQ ultrapure water (Millipore S.A.S., Alsace, France) and incubated in fixation solution containing 30% EtOH / 10% acetic acid / 60% MilliQ water for 15 min in order to fix proteins on the gel, and were then washed in MilliQ water three times for 5 min each. Gels were then incubated in EZBlue^™^ Gel Staining Reagent (Sigma-Aldrich, Saint-Quentin Fallavier, France) at room temperature with slight agitation for 2h, and de-stained in MilliQ water at room temperature overnight. For the Pooled Samples Experiment, each gel lane was fractionated into 20 pieces, which were excised from the gel and processed separately thereafter (Fig 1). In the Multiple Samples Experiment, the HMW and LMW fractions were excised from the gel and processed separately thereafter (Fig 1).

### In-gel tryptic digestion and LC-MS/MS

Gel pieces (fractions) were repeatedly washed in MilliQ water followed by incubation in ammonium bicarbonate (Ambic) 100mM: acetonitrile (ACN) 100% (1:1, v:v) until complete de-coloration and were then dried at 37°C for 20 min before reduction and alkylation. The dried fractions were incubated in 65 mM DTT at 37°C for 15 min followed by incubation at room temperature in the dark for 15 min after addition of 135 mM iodoacetamide, after which they were subjected to several washes in 100 mM Ambic: 100% ACN (1:1, v:v) and dried at 37°C for 20 min. Dried gel pieces were rehydrated in 50 mM Ambic containing sequencing grade modified trypsin (Promega, Charbonnières-les-Bains, France) at a final concentration of 12.5 ng/μl and incubated overnight at 37°C. Liquid containing protein digests was recovered by pipette and the remaining gel pieces were treated with 70% ACN: 0.1% formic acid with agitation at room temperature for 20 min. Liquid containing protein digests was collected and pooled with the previously collected digest and this elution step was repeated once again before the pooled digests were evaporated to dryness in a vacuum centrifuge. Pellets containing digested peptides were then resolubilized in 20 μl of 95% H_2_O: 5% formic acid by vortex mixing for 10 min before being subjected to LC-MS/MS.

Peptide mixtures were analyzed with a nanoflow high-performance liquid chromatography (HPLC) system (LC Packings Ultimate 3000, Thermo Fisher Scientific, Courtaboeuf, France) connected to a hybrid LTQ-OrbiTrap XL spectrometer (Thermo Fisher Scientific) equipped with a nanoelectrospray ion source (New Objective), as previously described [54]. The mass spectrometer was operated in the data-dependent mode by automatic switching between full-survey scan MS and consecutive MS/MS acquisition. Survey full scan MS spectra (mass range 400–2000) were acquired in the OrbiTrap section of the instrument with a resolution of r = 60,000 at m/z 400; ion injection times are calculated for each spectrum to allow for accumulation of 10^6^ ions in the OrbiTrap. The ten most intense peptide ions in each survey scan with an intensity above 2000 were sequentially isolated and fragmented in the linear ion trap by collision-induced dissociation. For OrbiTrap measurements, an external calibration was used before each injection series ensuring an overall error mass accuracy below 5 ppm for the detected peptides. MS data were saved in RAW file format (Thermo Fisher Scientific) using XCalibur 2.0.7 with tune 2.4.

The mass spectrometry proteomics data have been deposited to the ProteomeXchange Consortium [55] via the PRIDE [56] partner repository under the project name “Proteomic portraits of egg quality in zebrafish (*Danio rerio*)” with the Pooled Samples Experiment dataset accession number PXD005137 and project DOI number 10.6019/PXD005137, and the Multiple Samples Experiment dataset accession number PXD005129 and project DOI number 10.6019/PXD005129.

### Protein identification, quantification, annotation and statistics

The spectra search was performed with the Proteome Discoverer 1.2 software supported by Mascot (Mascot server v2.2.07; http://www.matrixscience.com). Obtained MS/MS spectra were searched against a target-decoy concatenated database created from the zebrafish Ensembl proteome database (Danio rerio_Zv9, March 2015) using Mascot (Matrix Science). Mass tolerance was set to 10 ppm and 0.5 Daltons for MS and MS/MS, respectively. Enzyme selectivity was set to full trypsin, with one miscleavage allowed. The allowed protein modifications were fixed carbamidomethylation of cysteines and variable oxidation of methionine. Attributed spectra were then analyzed using ProteoIQ 2.8 (Premier Biosoft, Palo Alto, CA, USA) at < 1% FDR, 0.5% minimum protein group probability, and 6 aa minimum peptide length, in order to identify egg quality group-specific and common proteins and quantify them based on their N-SC values. For each protein, obtained spectral counts were normalized in three sequential steps; a) normalization by apportion of shared peptides based on the number of unique peptides each protein group possessed, b) normalization by the total spectral counts between replicates and biological samples, and c) normalization by the size of each protein (amino acid residues).

Protein annotations were performed using the GO, KEGG and Database for Annotation, Visualization and Integrated Discovery (DAVID [57, 58]) functional annotation tools. Only proteins exhibiting a ≥2-fold difference in N-SC between egg quality groups, or proteins unique to an egg quality group, were considered to be differentially expressed between groups and subjected to further analyses. Differentially expressed proteins detected in the HMW and LMW fractions in the Multiple Samples Experiment were combined for classifications by functional category and for enrichment analyses; however they were submitted individually to statistical analyses conducted to detect significant differences in mean N-SC values between egg quality groups.

Differentially expressed proteins were classified into thirteen arbitrarily chosen functional categories that would account for ≥ 90% of the proteins. These functional categories are: protein synthesis (PS), energy metabolism (EM), lipid metabolism (LM), cell cycle, division, growth and fate (CC), protein degradation and synthesis inhibition (PD), apoptosis-related (AP), oncogene-related (OG), endosome/lysosome-related (EL), immune function-related (IF), oxidoreductase (REDOX)- and detoxification (Detox)-related (RD), lectins, ZPs, and Vtgs. Up-regulated proteins that could not be attributed to any of these categories and were placed in the category “other”. For simplicity, proteins were attributed to only one category considered as the ‘best’ fit. Presented results are based on consensus annotations of two independent observers made before any other analyses categorizing the proteins (i.e. observations made ‘blind’). Chi square analysis with significance level of (p≤0.05) after Benjamini Hochberg correction for multiple tests was used to detect differences between egg quality groups in the distribution of up-regulated proteins among functional categories.

Enrichment analyses were conducted separately for each experiment using the PANTHER-GO Slim enrichment tool from GO Consortium [59] available online at http://geneontology.org/ for Biological Process, Molecular Function, Protein Class and Cellular Component. Upregulated proteins from both experiments were subjected to a PANTHER Pathway [60] analysis and proteins attributed to enriched pathways (n = 74) were pooled before being submitted to analysis using the STRING search tool for retrieval of protein-protein interaction networks [61] available from the STRING Consortium online at http://string-db.org/, with the data settings Confidence: High (0.70), Max Number of Interactions to Show: None/query proteins only. For all PANTHER analyses, only statistically significant enrichment results (*p*<0.05 after Bonferroni correction for multiple tests) are shown.

To detect significant differences between egg quality groups in mean N-SC values for differentially expressed proteins from the HMW and LMW fractions (Multiple Samples Experiment) an independent t-test (p<0.05) followed by Benjamini Hochberg correction for multiple tests (p<0.1) was used (IBM SPSS Statistics Version 19.0.0, Armonk, NY).

## Supporting information

S1 Table Proteins differentially regulated in the Pooled Samples Experiment. (PDF)

S2 Table Proteins differentially regulated in the Multiple Samples Experiment. (PDF)

S3 Table Enrichment of PANTHER pathways with differentially regulated proteins. (PDF)

S1 Fig Enrichment of Molecular Function GO terms with differentially regulated proteins. (PDF)

S2 Fig Enrichment of Protein Class GO terms with differentially regulated proteins. (PDF)

S3 Fig Enrichment of Cellular Component GO terms with differentially regulated proteins. (PDF)

S4 Fig Diagrams of PANTHER pathways enriched with differentially regulated proteins. (PDF)
