# Supplementary material for: Scrambled eggs: Proteomic portraits and novel biomarkers of egg quality in zebrafish (Danio rerio)
Source: PLoS One. 2017 Nov 16;12(11):e0188084. doi: 10.1371/journal.pone.0188084 (PMC5690628; doi:10.1371/journal.pone.0188084)
Supplement: S1 Table — (PDF) [file pone.0188084.s001.pdf]

S1 Table. Proteins differentially regulated in the Pooled Samples Experiment. List of the 892 proteins from the Pooled Samples Experiment that were considered to be differentially regulated between egg quality groups and whose distribution among various functional categories is illustrated in **Fig 2a**. These include proteins detected only in poor quality eggs (PQ UNIQUE, n=211), proteins in poor quality eggs with N-SC increased ≥2-fold relative to values for good quality eggs (PQ INCREASED, n=186), proteins detected only in good quality eggs (GQ UNIQUE, n=136), and proteins in good quality eggs with N-SC increased ≥2-fold relative to values for poor quality eggs (GQ INCREASED, n=359). For each protein, the Ensembl Protein ID and associated gene, transcript and protein name, functional category (**Fig 2a**), relative abundance (UNIQUE or INCREASED), and fold-difference in N-SC between egg quality groups (if available) is shown. Color shading corresponds to that used to designate functional categories in **Fig 2**.

	Ensembl Protein ID	Associated Gene Name	Associated Transcript Name	Protein Full Name	Functional Category	Regulation	Fold Difference
1	ENSDARP00000030257	si:ch211-226h8.8	si:ch211-226h8.8-201	Uncharacterized protein containing SUEL type lectin domain	Lectins	PQ UNIQUE	
2	ENSDARP00000113274	si:ch211-226h8.8	si:ch211-226h8.8-001	Uncharacterized protein containing SUEL type lectin domain	Lectins	PQ UNIQUE	-
3	ENSDARP00000121868	zgc:172218	Novel (zgc17218-001)	Uncharacterized protein contining 3SUEL type lectin domains	Lectins	PQ UNIQUE	-
4	ENSDARP00000117390	si:ch211-226h8.11	si:ch211-226h8.11-001	L-rhamnose-binding lectin CSL3-like isoform X1	Lectins	PQ UNIQUE	-
5	ENSDARP00000061148	ENSDARP00000122263	zgc:55461-001	TUBB - tubulin, beta 4A class IVa-like	Cell cycle, division, growth and fate	PQ UNIQUE	-
6	ENSDARP00000072581	zgc:172218	Novel (zgc172218-201)	Uncharacterized protein--w 3 lectin domains	Lectins	PQ UNIQUE	-
7	ENSDARP00000104203	AL929192.3	AL929192.3-201	Novel protein containing galactose binding lectin domains	Lectins	PQ UNIQUE	-
8	ENSDARP00000095074	cnp	cnp-201	2',3'-cyclic nucleotide 3' phosphodiesterase	Cell cycle, division, growth and fate	PQ UNIQUE	-
9	ENSDARP00000053151	cbr1	cbr1-001	Carbonyl reductase 1	Lipid metabolism	PQ UNIQUE	-
10	ENSDARP00000024748	zgc:86598	zgc:86598-001	Uncharacterized protein--casein kinase family protein	Other	PQ UNIQUE	-
11	ENSDARP00000068088	hp	hp-201	Haptoglobin	Immune system related	PQ UNIQUE	-
12	ENSDARP00000108424	zgc:86598	zgc:86598-002	Protien kinase	Protein Synthesis	PQ UNIQUE	-
13	ENSDARP00000106833	ZP2 (4 of 4)	ZP2 (4 of 4)-201	Zona pellucida glycoprotein 2 (sperm receptor)	Zona Pellucida proteins	PQ UNIQUE	-
14	ENSDARP00000117065	zgc:152652	zgc:152652-001	Uncharacterized protein	Other	PQ UNIQUE	-
15	ENSDARP00000109929	zgc:171474	zgc:171474-001	Uncharacterized protein	Other	PQ UNIQUE	-
16	ENSDARP00000125025	hp	hp-001	Haptoglobin	Immune system related	PQ UNIQUE	-
17	ENSDARP00000116911	ck2a1	ck2a1-003	Casein kinase 2 alpha 1	Protein Synthesis	PQ UNIQUE	-
18	ENSDARP00000116024	ck2a1	ck2a1-004	Casein kinase 2 alpha 1	Protein Synthesis	PQ UNIQUE	-
19	ENSDARP00000129511	wdfy1	wdfy1-001	WD repeat and FYVE domain containing 1	Endosome-Lysosome related	PQ UNIQUE	-
20	ENSDARP00000103118	zgc:100918	Novel	uncharacterized Rab7-like, ras-related, protein	Endosome-Lysosome related	PQ UNIQUE	-
21	ENSDARP00000111138	zgc:100918	Novel	uncharacterized Rab7a-like, ras-related, protein	Endosome-Lysosome related	PQ UNIQUE	-
22	ENSDARP00000113694	ck2a1	ck2a1-002	Casein kinase 2 alpha 1	Protein Synthesis	PQ UNIQUE	-
23	ENSDARP00000014852	itln3	itln3-201	Intelectin 3	Immune system related	PQ UNIQUE	-
24	ENSDARP00000042593	hn1b	hn1b-001	Hematological and neurological expressed 1b	Cell cycle, division, growth and fate	PQ UNIQUE	-
25	ENSDARP00000110275	FARS2 (2 of 2)	FARS2 (2 of 2)-201	Phenylalanine-tRNA synthetase 2	Protein Synthesis	PQ UNIQUE	-
26	ENSDARP00000122499	CU467646.3	CU467646.3-201	Uncharacterized protein	Other	PQ UNIQUE	-
27	ENSDARP00000006001	ck2a1	ck2a1-001	Casein kinase 2 alpha 1	Protein Synthesis	PQ UNIQUE	-
28	ENSDARP00000056550	ck2a1	ck2a1-201	Casein kinase 2 alpha 1	Protein Synthesis	PQ UNIQUE	-
29	ENSDARP00000103535	BX548000.1	BX548000.1-201	Uncharacterized protein	Other	PQ UNIQUE	-
30	ENSDARP00000016540	rab11al	rab11al-001	RAB11a, member RAS oncogene family, like	Oncogenes related	PQ UNIQUE	-
31	ENSDARP00000124572	hp	hp-002	Haptoglobin	Immune system related	PQ UNIQUE	-
32	ENSDARP00000053863	gcdh	gcdh-201	Glutaryl-Coenzyme A dehydrogenase	Lipid metabolism	PQ UNIQUE	-
33	ENSDARP00000112983	gcdh	gcdh-001	Glutaryl-Coenzyme A dehydrogenase	Lipid metabolism	PQ UNIQUE	-
34	ENSDARP00000120266	C9H21orf33	C9H21orf33-001	Chromosome 21 open reading frame 33	Energy metabolism	PQ UNIQUE	-
35	ENSDARP00000071656	cox7a3	cox7a3-001	Cytochrome c oxidase subunit VIIa polypeptide 3	Energy metabolism	PQ UNIQUE	-
36	ENSDARP00000125277	USMG5	USMG5-001	Up-regulated during skeletal muscle growth 5 homolog (mouse)	Cell cycle, division, growth and fate	PQ UNIQUE	-
37	ENSDARP00000125356	USMG5	USMG5-002	Up-regulated during skeletal muscle growth 5 homolog (mouse)	Cell cycle, division, growth and fate	PQ UNIQUE	-
38	ENSDARP00000112178	fgg	fgg-201	Fibrinogen, gamma polypeptide	Immune system related	PQ UNIQUE	-
39	ENSDARP00000124470	si:dkey-87o1.2	si:dkey-87o1.2-001	Uncharacterized protein	Other	PQ UNIQUE	-
40	ENSDARP00000121763	coro1cb	coro1cb-002	Coronin, actin binding protein, 1Cb	Endosome-Lysosome related	PQ UNIQUE	-
41	ENSDARP00000108470	SMIM20	SMIM20-201	Small integral membrane protein 20	Other	PQ UNIQUE	-
42	ENSDARP00000049002	atp6v1aa-001	atp6v1aa-001	ATPase, H+ transporting, lysosomal V1 subunit Aa	Endosome-Lysosome related	PQ UNIQUE	-
43	ENSDARP00000116576	fkbp3	fkbp3-001	FK506 binding protein 3	Protein Synthesis	PQ UNIQUE	-
44	ENSDARP00000122221	ccbl1	ccbl1-003	Cysteine conjugate-beta lyase; cytoplasmic (glutamine transaminase K, kyneurenine aminotransferase)	Oncogenes related	PQ UNIQUE	-
45	ENSDARP00000063415	rnasel2	rnasel2-001	Ribonuclease like 2	Immune system related	PQ UNIQUE	-
46	ENSDARP00000113451	si:dkeyp-20e4.8	si:dkeyp-20e4.8-001	Uncharacterized protein	Other	PQ UNIQUE	-
47	ENSDARP00000068889	tia1	tia1-001	Cytotoxic granule-associated RNA binding protein 1	Apoptosis related	PQ UNIQUE	-
48	ENSDARP00000003248	zgc:77118	zgc:77118-001	Palmitoyl-(protein) hydrolase activity	Other	PQ UNIQUE	-
49	ENSDARP00000126272	actr10	actr10-001	Actin-related protein 10 homolog (S. cerevisiae)	Cell cycle, division, growth and fate	PQ UNIQUE	-
50	ENSDARP00000123855	si:dkey-87o1.2	si:dkey-87o1.2-003	Uncharacterized protein	Other	PQ UNIQUE	-
51	ENSDARP00000124841	si:dkey-87o1.2	si:dkey-87o1.2-002	Uncharacterized protein	Other	PQ UNIQUE	-
52	ENSDARP00000128259	sh3glb2a	sh3glb2a-004	SH3-domain GRB2-like endophilin B2a	Endosome-Lysosome related	PQ UNIQUE	-
53	ENSDARP00000026575	rbm8a	rbm8a-001	RNA binding motif protein 8A	Protein Synthesis	PQ UNIQUE	-
54	ENSDARP00000114997	hmgb2a	hmgb2a-003	High-mobility group box 2a	Apoptosis related	PQ UNIQUE	-
55	ENSDARP00000124034	psmc1a	psmc1a-002	Proteasome (prosome, macropain) 26S subunit, ATPase, 1a	Protein degradation and synthesis inhibition	PQ UNIQUE	-
56	ENSDARP00000090477	si:ch73-95l15.3	si:ch73-95l15.3-201	Fatty acid elongation in litochondria-lysosome	Lipid metabolism	PQ UNIQUE	-
57	ENSDARP00000128868	si:ch73-95l15.3	si:ch73-95l15.3-001	Fatty acid elongation in litochondria-lysosome	Lipid metabolism	PQ UNIQUE	-
58	ENSDARP00000100627	purbb	purbb-201	Purine-rich element binding protein Bb	Protein Synthesis	PQ UNIQUE	-
59	ENSDARP00000120767	ufd1l	ufd1l-003	Ubiquitin fusion degradation 1-like	Protein degradation and synthesis inhibition	PQ UNIQUE	-

60	ENSDARP00000124354	mapk1	mapk1-004	Mitogen-activated protein kinase 1	Apoptosis related	PQ UNIQUE	-
61	ENSDARP00000117412	med20	med20-001	Mediator complex subunit 20	Protein Synthesis	PQ UNIQUE	-
62	ENSDARP00000126190	krt17	krt17-002	Keratin 15	Cell cycle, division, growth and fate	PQ UNIQUE	-
63	ENSDARP00000067637	med20	med20-201	Mediator complex subunit 20	Protein Synthesis	PQ UNIQUE	-
64	ENSDARP00000007602	ppp2r2ab	ppp2r2ab-201	Protein phosphatase 2, regulatory subunit B, alpha b	Protein Synthesis	PQ UNIQUE	-
65	ENSDARP00000117378	ppp2r2ab	ppp2r2ab-001	Protein phosphatase 2, regulatory subunit B, alpha b	Cell cycle, division, growth and fate	PQ UNIQUE	-
66	ENSDARP00000066854	fars2	fars2-001	phenylalanyl-tRNA synthetase 2, mitochondrial	Protein Synthesis	PQ UNIQUE	-
67	ENSDARP00000128723	sh3glb2a	sh3glb2a-001	SH3-domain GRB2-like endophilin B2a	Endosome-Lysosome related	PQ UNIQUE	-
68	ENSDARP00000118809	si:dkey-51e6.1	si:dkey-51e6.1-001	Uncharacterized protein	Other	PQ UNIQUE	-
69	ENSDARP00000061755	si:dkey-51e6.1	si:dkey-51e6.1-201	Uncharacterized protein	Other	PQ UNIQUE	-
70	ENSDARP00000110749	naa50	naa50-002	N(alpha)-acetyltransferase 50, NatE catalytic subunit	Oncogenes related	PQ UNIQUE	-
71	ENSDARP00000116767	comtd1	comtd1-002	Catechol-O-methyltransferase domain containing 1	Other	PQ UNIQUE	-
72	ENSDARP00000115915	casp3a	casp3a-004	Caspase 3, apoptosis-related cysteine protease a	Apoptosis related	PQ UNIQUE	-
73	ENSDARP00000111328	BX470131.2	BX470131.2-201	Uncharacterized protein--LOC568697 (LOC568697), mRNA	Other	PQ UNIQUE	-
74	ENSDARP00000112094	polr1c	polr1c-001	Polymerase (RNA) I polypeptide C	Protein Synthesis	PQ UNIQUE	-
75	ENSDARP00000127587	sh3glb2a	sh3glb2a-002	SH3-domain GRB2-like endophilin B2a	Endosome-Lysosome related	PQ UNIQUE	-
76	ENSDARP00000053935	pfdn6	pfdn6-001	Prefoldin subunit 6	Protein Synthesis	PQ UNIQUE	-
77	ENSDARP00000087463	nhp2l1a	nhp2l1a-001	NHP2 non-histone chromosome protein 2-like 1a (S. cerevisiae)	Protein Synthesis	PQ UNIQUE	-
78	ENSDARP00000127398	fntb	fntb-003	Farnesyltransferase, CAAX box, beta	Other	PQ UNIQUE	-
79	ENSDARP00000006309	wdfy1	wdfy1-201	WD repeat and FYVE domain containing 1	Endosome-Lysosome related	PQ UNIQUE	-
80	ENSDARP00000100638	atp6v0d1	atp6v0d1-201	ATPase, H+ transporting, V0 subunit D isoform 1	Endosome-Lysosome related	PQ UNIQUE	-
81	ENSDARP00000113069	NAPB (2 of 2)	NAPB (2 of 2)-001	N-ethylmaleimide-sensitive factor attachment protein, beta	Protein Synthesis	PQ UNIQUE	-
82	ENSDARP00000127072	scrn2	scrn2-002	Secernin 2	Protein degradation and synthesis inhibition	PQ UNIQUE	-
83	ENSDARP00000129285	ECH1 (2 of 2)	ECH1 (2 of 2)-002	Enoyl CoA hydratase 1, peroxisomal	Lipid metabolism	PQ UNIQUE	-
84	ENSDARP00000059475	psmg1	psmg1-001	Proteasome (prosome, macropain) assembly chaperone 1	Protein degradation and synthesis inhibition	PQ UNIQUE	-
85	ENSDARP00000090111	actr10	actr10-201	Actin-related protein 10 homolog (S. cerevisiae)	Cell cycle, division, growth and fate	PQ UNIQUE	-
86	ENSDARP00000105905	CU928126.3	CU928126.3-201	Casein kinase II subunit alpha-like isoform X1	Protein Synthesis	PQ UNIQUE	-
87	ENSDARP00000115880	comtd1	comtd1-001	Catechol-O-methyltransferase domain containing 1	Other	PQ UNIQUE	-
88	ENSDARP00000094516	ppp4ca	ppp4ca-001	Protein phosphatase 4 (formerly X), catalytic subunit a	Apoptosis related	PQ UNIQUE	-
89	ENSDARP00000124293	cbx1b	cbx1b-003	Chromobox homolog 1b (HP1 beta homolog Drosophila)	Cell cycle, division, growth and fate	PQ UNIQUE	-
90	ENSDARP00000101062	purba	purba-001	Purine-rich element binding protein Ba	Protein Synthesis	PQ UNIQUE	-
91	ENSDARP00000125785	seh1l	seh1l-002	SEH1-like (S. cerevisiae)	Cell cycle, division, growth and fate	PQ UNIQUE	-
92	ENSDARP00000112994	zgc:171977	zgc:171977-001	Uncharacterized protein	Other	PQ UNIQUE	-
93	ENSDARP00000122910	snx12	snx12-005	Sorting nexin 12	Endosome-Lysosome related	PQ UNIQUE	-
94	ENSDARP00000008027	snx12	snx12-001	Sorting nexin 12	Endosome-Lysosome related	PQ UNIQUE	-
95	ENSDARP00000122028	pbdc1	pbdc1-001	Polysaccharide biosynthesis domain containing 1	Protein degradation and synthesis inhibition	PQ UNIQUE	-
96	ENSDARP00000123575	hmgb2a	hmgb2a-004	High-mobility group box 2a	Apoptosis related	PQ UNIQUE	-
97	ENSDARP00000088891	ddrgk1	ddrgk1-202	DDRGK domain containing 1	Protein degradation and synthesis inhibition	PQ UNIQUE	-
98	ENSDARP00000004839	dnajb11	dnajb11-201	DnaJ (Hsp40) homolog, subfamily B, member 11	Protein Synthesis	PQ UNIQUE	-
99	ENSDARP00000107433	dnajb11	dnajb11-001	DnaJ (Hsp40) homolog, subfamily B, member 11	Protein Synthesis	PQ UNIQUE	-
100	ENSDARP00000122189	nasp	nasp-003	Nuclear autoantigenic sperm protein (histone-binding)	Cell cycle, division, growth and fate	PQ UNIQUE	-
101	ENSDARP00000004932	myl1	myl1-001	Myosin, light chain 1, alkali; skeletal, fast	Cell cycle, division, growth and fate	PQ UNIQUE	-
102	ENSDARP00000051402	sh3glb2a	sh3glb2a-201	SH3-domain GRB2-like endophilin B2a	Endosome-Lysosome related	PQ UNIQUE	-
103	ENSDARP00000072059	cfb	cfb-001	Complement factor B	Immune system related	PQ UNIQUE	-
104	ENSDARP00000106788	ctsa	ctsa-201	Cathepsin A	Protein degradation and synthesis inhibition	PQ UNIQUE	-
105	ENSDARP00000017647	gins1	gins1-201	GINS complex subunit 1 (Psf1 homolog)	Cell cycle, division, growth and fate	PQ UNIQUE	-
106	ENSDARP00000119427	uba1	uba1-003	Ubiquitin-like modifier activating enzyme 1	Protein degradation and synthesis inhibition	PQ UNIQUE	-
107	ENSDARP00000128686	sh3glb2a	sh3glb2a-003	SH3-domain GRB2-like endophilin B2a	Endosome-Lysosome related	PQ UNIQUE	-
108	ENSDARP00000039666	cbx1b	cbx1b-201	Chromobox homolog 1b (HP1 beta homolog Drosophila)	Cell cycle, division, growth and fate	PQ UNIQUE	-
109	ENSDARP00000105344	novel-actn4_rat	Novel	Novel	Oncogenes related	PQ UNIQUE	-
110	ENSDARP00000113004	cbx1b	cbx1b-001	Chromobox homolog 1b (HP1 beta homolog Drosophila)	Cell cycle, division, growth and fate	PQ UNIQUE	-
111	ENSDARP00000127314	capgb	capgb-002	Capping protein (actin filament), gelsolin-like b	Cell cycle, division, growth and fate	PQ UNIQUE	-
112	ENSDARP00000123185	actn1	actn1-003	Actinin, alpha 1	Cell cycle, division, growth and fate	PQ UNIQUE	-
113	ENSDARP00000095774	krt17	krt17-001	Keratin 15	Cell cycle, division, growth and fate	PQ UNIQUE	-
114	ENSDARP00000118428	psmb7	psmb7-003	Proteasome (prosome, macropain) subunit, beta type, 7	Protein degradation and synthesis inhibition	PQ UNIQUE	-
115	ENSDARP00000118656	cbx1a	cbx1a-001	Chromobox homolog 1a (HP1 beta homolog Drosophila)	Protein degradation and synthesis inhibition	PQ UNIQUE	-
116	ENSDARP00000031553	eif4e1c	eif4e1c-001	Eukaryotic translation initiation factor 4E family member 1c	Protein Synthesis	PQ UNIQUE	-
117	ENSDARP00000047158	hmgb2a	hmgb2a-001	High-mobility group box 2a	Apoptosis related	PQ UNIQUE	-
118	ENSDARP00000016201	tm9sf2	tm9sf2-001	Transmembrane 9 superfamily member 2	Endosome-Lysosome related	PQ UNIQUE	-
119	ENSDARP00000061395	chmp5b	chmp5b-001	charged multivesicular body protein 5b	Endosome-Lysosome related	PQ UNIQUE	-
120	ENSDARP00000127370	eef1db	eef1db-005	Elongation factor-1, delta, b	Protein Synthesis	PQ UNIQUE	-
121	ENSDARP00000129156	napba	napba-001	N-ethylmaleimide-sensitive factor attachment protein, beta a	Protein degradation and synthesis inhibition	PQ UNIQUE	-
122	ENSDARP00000009369	krt15	krt15-201	Keratin 15	Cell cycle, division, growth and fate	PQ UNIQUE	-
123	ENSDARP00000095029	krt15	krt15-001	Keratin 15	Cell cycle, division, growth and fate	PQ UNIQUE	-
124	ENSDARP00000119224	eif4e1c	eif4e1c-003	Eukaryotic translation initiation factor 4E family member 1c	Protein Synthesis	PQ UNIQUE	-
125	ENSDARP00000128345	fars2	fars2-201	Phenylalanine-tRNA synthetase 2	Protein Synthesis	PQ UNIQUE	-
126	ENSDARP00000024741	napba	napba-201	N-ethylmaleimide-sensitive factor attachment protein, beta a	Protein degradation and synthesis inhibition	PQ UNIQUE	-
127	ENSDARP00000028134	psma6b	psma6b-001	Proteasome (prosome, macropain) subunit, alpha type, 6b	Protein degradation and synthesis inhibition	PQ UNIQUE	-

128	ENSDARP00000066106	bcap31	bcap31-001	B-cell receptor-associated protein 31	Protein Synthesis	PQ UNIQUE	-
129	ENSDARP00000108625	bcap31	bcap31-201	B-cell receptor-associated protein 31	Protein Synthesis	PQ UNIQUE	-
130	ENSDARP00000098086	cfb	cfb-201	Complement factor B	Immune system related	PQ UNIQUE	-
131	ENSDARP00000060174	si:dkey-12e7.4	si:dkey-12e7.4-001	Oxidoreductase-NAD(p) binding domain	Lipid metabolism	PQ UNIQUE	-
132	ENSDARP00000112303	psmb7	psmb7-001	Proteasome (prosome, macropain) subunit, beta type, 7	Protein degradation and synthesis inhibition	PQ UNIQUE	-
133	ENSDARP00000035106	PURA (1 of 2)	PURA (1 of 2)-201	Purine-rich element binding protein A [	Cell cycle, division, growth and fate	PQ UNIQUE	-
134	ENSDARP00000059013	zgc:173443	zgc:173443-001	Uncharacterized protein	Other	PQ UNIQUE	-
135	ENSDARP00000055324	psmb7	psmb7-002	Proteasome (prosome, macropain) subunit, beta type, 7	Protein degradation and synthesis inhibition	PQ UNIQUE	-
136	ENSDARP00000101909	pura	pura-002	Purine-rich element binding protein A	Protein Synthesis	PQ UNIQUE	-
137	ENSDARP00000112873	snx12	snx12-002	Sorting nexin 12	Endosome-Lysosome related	PQ UNIQUE	-
138	ENSDARP00000122416	pura	pura-001	Purine-rich element binding protein A	Protein Synthesis	PQ UNIQUE	-
139	ENSDARP00000088108	NAPA (2 of 2)	NAPA (2 of 2)-001	N-ethylmaleimide-sensitive factor attachment protein, alpha	Endosome-Lysosome related	PQ UNIQUE	-
140	ENSDARP00000021426	zgc:66479	zgc:66479-001	Uncharacterized protein	Other	PQ UNIQUE	-
141	ENSDARP00000033064	wdr61	wdr61-201	WD repeat domain 61	Cell cycle, division, growth and fate	PQ UNIQUE	-
142	ENSDARP00000110861	wdr61	wdr61-202	WD repeat domain 61	Cell cycle, division, growth and fate	PQ UNIQUE	-
143	ENSDARP00000025772	syap1	syap1-001	Synapse associated protein 1	Other	PQ UNIQUE	-
144	ENSDARP00000026028	hmgcl	hmgcl-001	3-hydroxymethyl-3-methylglutaryl-Coenzyme A lyase	Lipid metabolism	PQ UNIQUE	-
145	ENSDARP00000104510	hmgcl	hmgcl-201	3-hydroxymethyl-3-methylglutaryl-Coenzyme A lyase	Lipid metabolism	PQ UNIQUE	-
146	ENSDARP00000129523	glg1a	glg1a-002	Golgi glycoprotein 1a	Cell cycle, division, growth and fate	PQ UNIQUE	-
147	ENSDARP00000045487	ck2a2b	ck2a2b-201	Casein kinase 2 alpha 2b	Apoptosis related	PQ UNIQUE	-
148	ENSDARP00000103569	actl6b	actl6b-201	Actin-like 6B	Protein Synthesis	PQ UNIQUE	-
149	ENSDARP00000024096	ACTR2 (1 of 3)	ACTR2 (1 of 3)-001	ARP2 actin-related protein 2 homolog (yeast)	Oncogenes related	PQ UNIQUE	-
150	ENSDARP00000057868	mat1a	mat1a-001	Methionine adenosyltransferase I, alpha	Protein Synthesis	PQ UNIQUE	-
151	ENSDARP00000124307	actl6b	actl6b-001	Actin-like 6B	Protein Synthesis	PQ UNIQUE	-
152	ENSDARP00000071166	aars	aars-201	Alanyl-tRNA synthetase	Protein Synthesis	PQ UNIQUE	-
153	ENSDARP00000088880	viml	viml-201	Vimentin like	Cell cycle, division, growth and fate	PQ UNIQUE	-
154	ENSDARP00000016261	vim	vim-001	Vimentin	Cell cycle, division, growth and fate	PQ UNIQUE	-
155	ENSDARP00000023622	bfb	bfb-001	Complement component bfb	Endosome-Lysosome related	PQ UNIQUE	-
156	ENSDARP00000107935	bfb	bfb-201	Complement component bfb	Endosome-Lysosome related	PQ UNIQUE	-
157	ENSDARP00000113857	coro1cb	coro1cb-001	Coronin, actin binding protein, 1Cb	Endosome-Lysosome related	PQ UNIQUE	-
158	ENSDARP00000039795	coro1cb	coro1cb-201	Coronin, actin binding protein, 1Cb	Endosome-Lysosome related	PQ UNIQUE	-
159	ENSDARP00000091174	aars	aars-001	Alanyl-tRNA synthetase	Protein Synthesis	PQ UNIQUE	-
160	ENSDARP00000012553	aaas	aaas-201	Achalasia, adrenocortical insufficiency, alacrimia	Cell cycle, division, growth and fate	PQ UNIQUE	-
161	ENSDARP00000110386	aars	aars-202	Alanyl-tRNA synthetase	Protein Synthesis	PQ UNIQUE	-
162	ENSDARP00000120649	aars	aars-002	Alanyl-tRNA synthetase	Protein Synthesis	PQ UNIQUE	-
163	ENSDARP00000105615	CU695117.1	CU695117.1-201	Zinc finger MYM-type protein 1	Protein synthesis	PQ UNIQUE	-
164	ENSDARP00000109275	CU929506.1	CU929506.1-201	Zinc finger MYM-type protein 1	Protein synthesis	PQ UNIQUE	-
165	ENSDARP00000074082	C4B	C4B-201	Complement component 4B (Chido blood group)	Immune system related	PQ UNIQUE	-
166	ENSDARP00000115743	C4B	C4B-001	Complement component 4B (Chido blood group)	Immune system related	PQ UNIQUE	-
167	ENSDARP00000000803	actn3b	actn3b-001	Actinin alpha 3b	Cell cycle, division, growth and fate	PQ UNIQUE	-
168	ENSDARP00000005224	actn3a	actn3a-001	Actinin alpha 3b	Cell cycle, division, growth and fate	PQ UNIQUE	-
169	ENSDARP00000090990	actn1	actn1-201	Actinin, alpha 1	Cell cycle, division, growth and fate	PQ UNIQUE	-
170	ENSDARP00000110976	actn1	actn1-202	Actinin, alpha 1	Cell cycle, division, growth and fate	PQ UNIQUE	-
171	ENSDARP00000120755	actn1	actn1-002	Actinin, alpha 1	Cell cycle, division, growth and fate	PQ UNIQUE	-
172	ENSDARP00000123299	actn1	actn1-001	Actinin, alpha 1	Cell cycle, division, growth and fate	PQ UNIQUE	-
173	ENSDARP00000077418	clip2	clip2-201	CAP-GLY domain containing linker protein 2	Cell cycle, division, growth and fate	PQ UNIQUE	-
174	ENSDARP00000124509	clip2	clip2-001	CAP-GLY domain containing linker protein 2	Cell cycle, division, growth and fate	PQ UNIQUE	-
175	ENSDARP00000109853	hdlbpb	hdlbpb-201	High density lipoprotein-binding protein b	Lipid metabolism	PQ UNIQUE	-
176	ENSDARP00000127252	hdlbpb	hdlbpb-001	High density lipoprotein-binding protein b	Lipid metabolism	PQ UNIQUE	-
177	ENSDARP00000052837	acta1a	acta1a-001	Actin alpha 1a skeletal muscle	Cell cycle, division, growth and fate	PQ UNIQUE	-
178	ENSDARP00000055135	actc1b	actc1b-201	Actin, alpha, cardiac muscle 1b	Cell cycle, division, growth and fate	PQ UNIQUE	-
179	ENSDARP00000055379	tubb5	tubb5-201	Tubulin, beta 5	Cell cycle, division, growth and fate	PQ UNIQUE	-
180	ENSDARP00000058628	acta1b	acta1b-001	Actin, alpha 1b, skeletal muscle	Cell cycle, division, growth and fate	PQ UNIQUE	-
181	ENSDARP00000062369	actc1a	actc1a-001	Actin, alpha, cardiac muscle 1a	Cell cycle, division, growth and fate	PQ UNIQUE	-
182	ENSDARP00000063082	zgc:174637	zgc:174637-201	Arylesterase activity	Immune system related	PQ UNIQUE	-
183	ENSDARP00000066429	acta2	acta2-201	Actin, alpha 2, smooth muscle, aorta	Cell cycle, division, growth and fate	PQ UNIQUE	-
184	ENSDARP00000066858	tubb4b	tubb4b-001	Tubulin, beta 4B class IVb	Cell cycle, division, growth and fate	PQ UNIQUE	-
185	ENSDARP00000069926	PPP2CA	PPP2CA-201	Protein phosphatase 2, catalytic subunit, alpha isozyme	Protein Synthesis	PQ UNIQUE	-
186	ENSDARP00000074935	ywhag1	ywhag1-001	3-monooxygenase/tryptophan 5-monooxygenase activation protein, gamma polypeptide 1	Cell cycle, division, growth and fate	PQ UNIQUE	-
187	ENSDARP00000075110	CH211-260D9.2	CH211-260D9.2-001	Actin-novel	Cell cycle, division, growth and fate	PQ UNIQUE	-
188	ENSDARP00000095894	si:dkey-90l23.2	Novel	L-rhamnose-binding lectin CSL3-like isoform X2	Lectins	PQ UNIQUE	-
189	ENSDARP00000096898	ywhag2	ywhag2-001	3-monooxygenase/tryptophan 5-monooxygenase activation protein, gamma polypeptide 2	Cell cycle, division, growth and fate	PQ UNIQUE	-
190	ENSDARP00000100195	zgc:86725	zgc:86725	Actin alpha 1 (ACTA1)-like	Cell cycle, division, growth and fate	PQ UNIQUE	-
191	ENSDARP00000100434	actc1a	Novel	Actin, alpha, cardiac muscle 1a, novel	Cell cycle, division, growth and fate	PQ UNIQUE	-
192	ENSDARP00000102472	vtg4	vtg4-202	Vitellogenin 4	Vitellogenins	PQ UNIQUE	-
193	ENSDARP00000103251	qdprb2	qdprb2-201	Quinoid dihydropteridine reductase b2	REDOX/Detox related	PQ UNIQUE	-
194	ENSDARP00000104702	qdprb2	qdprb2-001	Quinoid dihydropteridine reductase b2	REDOX/Detox related	PQ UNIQUE	-
195	ENSDARP00000105815	zgc:173556	zgc:173556-001	Zona pellucida glycoprotein 3.1	Zona Pellucida proteins	PQ UNIQUE	-

196	ENSDARP00000106988	si:dkeyp-98a7.5	si:dkeyp-98a7.5-202	Novel rhamnose binding lectin-like precursor	Lectins	PQ UNIQUE	-
197	ENSDARP00000107122	tubb4b	tubb4b-201	Tubulin, beta 4B class IVb	Cell cycle, division, growth and fate	PQ UNIQUE	-
198	ENSDARP00000107578	si:dkeyp-98a7.4	si:dkeyp-98a7.4-201	SUEL type lectin domain	Lectins	PQ UNIQUE	-
199	ENSDARP00000109226	AL929192.2	AL929192.2-201	Gal lectin superfamily	Lectins	PQ UNIQUE	-
200	ENSDARP00000110519	AL929192.1	AL929192.1-201	Gal lectin superfamily	Lectins	PQ UNIQUE	-
201	ENSDARP00000111434	si:dkeyp-98a7.5	si:dkeyp-98a7.5-201	SUEL type lectin domain	Lectins	PQ UNIQUE	-
202	ENSDARP00000111784	si:dkeyp-98a7.3	si:dkeyp-98a7.3-201	SUEL type lectin domain	Lectins	PQ UNIQUE	-
203	ENSDARP00000120702	si:dkeyp-98a7.5	si:dkeyp-98a7.5-001	SUEL type lectin domain	Lectins	PQ UNIQUE	-
204	ENSDARP00000124371	acta2	acta2-001	Actin, alpha 2, smooth muscle, aorta	Cell cycle, division, growth and fate	PQ UNIQUE	-
205	ENSDARP00000125193	si:dkeyp-98a7.7	si:dkeyp-98a7.7-001	SUEL type lectin domain	Lectins	PQ UNIQUE	-
206	ENSDARP00000125214	si:dkeyp-98a7.8	si:dkeyp-98a7.8-001	SUEL type lectin domain	Lectins	PQ UNIQUE	-
207	ENSDARP00000125238	si:dkeyp-98a7.4	si:dkeyp-98a7.4-001	SUEL type lectin domain	Lectins	PQ UNIQUE	-
208	ENSDARP00000125244	si:dkeyp-98a7.3	si:dkeyp-98a7.3-001	SUEL type lectin domain	Lectins	PQ UNIQUE	-
209	ENSDARP00000125496	si:ch211-250e5.9	si:ch211-250e5.9-001	SUEL type lectin domain	Lectins	PQ UNIQUE	-
210	ENSDARP00000125818	PPP2CA	PPP2CA-001	Protein phosphatase 2, catalytic subunit, alpha isozyme	Protein Synthesis	PQ UNIQUE	-
211	ENSDARP00000128683	ACTC1 (2 of 2)	ACTC1 (2 of 2)-001	Actin, alpha, cardiac muscle 1a	Cell cycle, division, growth and fate	PQ UNIQUE	-
212	ENSDARP00000094165	wu:fi42e03	wu:fi42e03-201	PAX-interacting protein 1	Cell cycle, division, growth and fate	PQ INCREASED	27.99
213	ENSDARP00000091393	ZP2 (2 of 4)	ZP2 (2 of 4)-001	Zona pellucida glycoprotein 2 (sperm receptor)	Zona Pellucida proteins	PQ INCREASED	12.17
214	ENSDARP00000116912	zgc:173856	zgc:173856-001	Nuclear receptor coactivayor 6-like	Protein Synthesis	PQ INCREASED	11.43
215	ENSDARP00000072211	zp2l1	zp2l1-001	Zona pellucida glycoprotein 2, like 1	Zona Pellucida proteins	PQ INCREASED	10.63
216	ENSDARP00000107576	suclg2	suclg2-201	Succinate-CoA ligase, GDP-forming, beta subunit	Energy metabolism	PQ INCREASED	9.58
217	ENSDARP00000068662	zp3.2	zp3.2-202	Zona pellucida glycoprotein 3, tandem duplicate 2	Zona Pellucida proteins	PQ INCREASED	9.13
218	ENSDARP00000122970	zp3b	zp3b-002	Zona pellucida glycoprotein 3b	Zona Pellucida proteins	PQ INCREASED	8.85
219	ENSDARP00000081954	suclg2	suclg2-001	Succinate-CoA ligase, GDP-forming, beta subunit	Energy metabolism	PQ INCREASED	8.74
220	ENSDARP00000071171	zp3b	zp3b-201	Zona pellucida glycoprotein 3b	Zona Pellucida proteins	PQ INCREASED	7.40
221	ENSDARP00000071703	alg1	alg1-001	Asparagine-linked glycosylation 1 homolog (yeast, beta-1,4-mannosyltransferase)	Protein Synthesis	PQ INCREASED	6.96
222	ENSDARP00000099884	ZP2 (3 of 4)	ZP2 (3 of 4)-201	Zona pellucida glycoprotein 2 (sperm receptor)	Zona Pellucida proteins	PQ INCREASED	6.54
223	ENSDARP00000058255	zp3b	zp3b-001	Zona pellucida glycoprotein 3b	Zona Pellucida proteins	PQ INCREASED	6.53
224	ENSDARP00000054229	fgg	fgg-001	Fibrinogen, gamma polypeptide	Immune system related	PQ INCREASED	6.11
225	ENSDARP00000088788	ERLIN2	ERLIN2-201	ER lipid raft associated 2 (erlin2), mRNA	Lipid metabolism	PQ INCREASED	6.10
226	ENSDARP00000121177	ERLIN2	ERLIN2-001	ER lipid raft associated 2 (erlin2), mRNA	Lipid metabolism	PQ INCREASED	6.10
227	ENSDARP00000124617	rpa2	rpa2-002	Replication protein A2	Cell cycle, division, growth and fate	PQ INCREASED	5.24
228	ENSDARP00000094806	sdf4	sdf4-001	Stromal cell derived factor 4	Immune system related	PQ INCREASED	5.22
229	ENSDARP00000122503	pus1	pus1-001	Pseudouridine synthase 1	Cell cycle, division, growth and fate	PQ INCREASED	5.22
230	ENSDARP00000121591	CRP	CRP-001	C-reactive protein, pentraxin-related	Immune system related	PQ INCREASED	5.22
231	ENSDARP00000054025	rcc1	rcc1-001	Regulator of chromosome condensation 1	Cell cycle, division, growth and fate	PQ INCREASED	5.20
232	ENSDARP00000127355	capgb	capgb-001	Capping protein (actin filament), gelsolin-like b	Cell cycle, division, growth and fate	PQ INCREASED	4.37
233	ENSDARP00000036766	zgc:165539	zgc:165539-201	Uncharacterized protein	Cell cycle, division, growth and fate	PQ INCREASED	4.36
234	ENSDARP00000029607	gfap	gfap-001	Glial fibrillary acidic protein	Cell cycle, division, growth and fate	PQ INCREASED	4.35
235	ENSDARP00000116942	zgc:165539	zgc:165539-001	Uncharacterized protein	Cell cycle, division, growth and fate	PQ INCREASED	4.35
236	ENSDARP00000107173	si:dkey-241l7.5	si:dkey-241l7.5-001	C type lectin	Lectins	PQ INCREASED	4.32
237	ENSDARP00000109765	zgc:171670	zgc:171670-001	C type lectin	Lectins	PQ INCREASED	4.32
238	ENSDARP00000127261	zgc:171670	zgc:171670-002	C type lectin	Lectins	PQ INCREASED	4.32
239	ENSDARP00000127453	si:dkey-241l7.5	si:dkey-241l7.5-002	C type lectin	Lectins	PQ INCREASED	4.32
240	ENSDARP00000018923	eif4a1b	eif4a1b-001	Eukaryotic translation initiation factor 4A, isoform 1B	Protein Synthesis	PQ INCREASED	4.04
241	ENSDARP00000128336	eif4a3	eif4a3-002	Eukaryotic translation initiation factor 4A, isoform 1B	Protein Synthesis	PQ INCREASED	4.01
242	ENSDARP00000061750	zp3a.2	zp3a.2-001	Zona pellucida glycoprotein 3a, tandem duplicate 2	Zona Pellucida proteins	PQ INCREASED	3.98
243	ENSDARP00000004016	fen1	fen1-201	Flap structure-specific endonuclease 1	Cell cycle, division, growth and fate	PQ INCREASED	3.91
244	ENSDARP00000043687	si:dkey-241l7.2	si:dkey-241l7.2-001	C type lectin	Lectins	PQ INCREASED	3.84
245	ENSDARP00000127204	si:dkey-241l7.2	si:dkey-241l7.2-002	C type lectin	Lectins	PQ INCREASED	3.84
246	ENSDARP00000127328	si:dkey-241l7.2	si:dkey-241l7.2-003	C type lectin	Lectins	PQ INCREASED	3.84
247	ENSDARP00000104065	zgc:173837	zgc:173837-201	Uncharacterized dopey family member 2-like protein	Endosome-Lysosome related	PQ INCREASED	3.80
248	ENSDARP00000070941	ap2a1	ap2a1-201	Adaptor-related protein complex 2, alpha 1 subunit	Protein degradation and synthesis inhibition	PQ INCREASED	3.53
249	ENSDARP00000105782	mat2aa	mat2aa-001	S-adenosylmethionine synthase-Methionine adenosyltransferase II, alpha a	Protein Synthesis	PQ INCREASED	3.50
250	ENSDARP00000028571	ndufs1	ndufs1-001	NADH dehydrogenase (ubiquinone) Fe-S protein 1	Energy metabolism	PQ INCREASED	3.49
251	ENSDARP00000118532	erlin2	erlin2-001	ER lipid raft associated 2 (erlin2), mRNA	Lipid metabolism	PQ INCREASED	3.49
252	ENSDARP00000110335	isoc1	isoc1-201	Isochorismatase domain containing 1	REDOX/Detox related	PQ INCREASED	3.49
253	ENSDARP00000063856	scrn2	scrn2-001	Secernin 2	Protein degradation and synthesis inhibition	PQ INCREASED	3.49
254	ENSDARP00000114795	casp3a	casp3a-003	Caspase 3, apoptosis-related cysteine protease a	Apoptosis related	PQ INCREASED	3.49
255	ENSDARP00000110330	HOMER2	HOMER2-201	Homer homolog 2 (Drosophila)	Other	PQ INCREASED	3.48
256	ENSDARP00000116562	sepp1a	sepp1a-001	Selenoprotein P, plasma, 1a	REDOX/Detox related	PQ INCREASED	3.48
257	ENSDARP00000090521	nap1l4b	nap1l4b-201	Nucleosome assembly protein 1-like 4b	Cell cycle, division, growth and fate	PQ INCREASED	3.48
258	ENSDARP00000115833	nap1l4b	nap1l4b-001	Nucleosome assembly protein 1-like 4b	Cell cycle, division, growth and fate	PQ INCREASED	3.48
259	ENSDARP00000076885	HOMER2	HOMER2-202	Homer homolog 2 (Drosophila)	Other	PQ INCREASED	3.48
260	ENSDARP00000057824	cfd	cfd-001	Complement factor D (adipsin)	Protein degradation and synthesis inhibition	PQ INCREASED	3.48
261	ENSDARP00000034563	fntb	fntb-001	Farnesyltransferase, CAAX box, beta	Cell cycle, division, growth and fate	PQ INCREASED	3.48
262	ENSDARP00000025160	psmd8	psmd8-001	Proteasome (prosome, macropain) 26S subunit, non-ATPase, 8	Protein Synthesis	PQ INCREASED	3.48
263	ENSDARP00000078713	isoc1	isoc1-001	Isochorismatase domain containing 1	REDOX/Detox related	PQ INCREASED	3.47

264	ENSDARP00000060245	dnajc3	dnajc3-001	DnaJ (Hsp40) homolog, subfamily C, member 3	Protein Synthesis	PQ INCREASED	3.47
265	ENSDARP00000128168	ap2a1	ap2a1-001	Adaptor-related protein complex 2, alpha 1 subunit	Protein degradation and synthesis inhibition	PQ INCREASED	3.44
266	ENSDARP00000073542	CR559930.2	CR559930.2-201	Serpin superfamily	Other	PQ INCREASED	3.40
267	ENSDARP00000005519	zgc:171927	zgc:171927-001	Uncharacterized protein	Apoptosis related	PQ INCREASED	3.35
268	ENSDARP00000100922	CABZ01059627.2	CABZ01059627.2-202	Mucin-5AC	Cell cycle, division, growth and fate	PQ INCREASED	3.35
269	ENSDARP00000107790	zp3a.1	zp3a.1-201	Zona pellucida glycoprotein 3a, tandem duplicate 1	Zona Pellucida proteins	PQ INCREASED	3.34
270	ENSDARP00000103071	zgc:173770	zgc:173770-201	Mucin-5AC-like	Cell cycle, division, growth and fate	PQ INCREASED	3.31
271	ENSDARP00000118880	rplp1	rplp1-002	Ribosomal protein, large, P1	Protein Synthesis	PQ INCREASED	3.14
272	ENSDARP00000027643	zgc:63587	zgc:63587-201	Uncharacterized protein	Cell cycle, division, growth and fate	PQ INCREASED	3.10
273	ENSDARP00000058955	ywhabl	ywhabl-001	Tyrosine 3-monooxygenase/tryptophan 5-monooxygenase activation protein, beta polypeptide like	Cell cycle, division, growth and fate	PQ INCREASED	3.08
274	ENSDARP00000103720	ywhabl	ywhabl-201	Tyrosine 3-monooxygenase/tryptophan 5-monooxygenase activation protein, beta polypeptide like	Cell cycle, division, growth and fate	PQ INCREASED	3.08
275	ENSDARP00000060688	eif2b2	eif2b2-001	Eukaryotic translation initiation factor 2B, subunit 2 beta	Protein Synthesis	PQ INCREASED	3.05
276	ENSDARP00000096076	si:ch211-226h8.6	si:ch211-226h8.6-001	Uncharacterized protein	Other	PQ INCREASED	3.05
277	ENSDARP00000122128	si:ch211-11k18.4	si:ch211-11k18.4-002	Uncharacterized protein	Other	PQ INCREASED	3.05
278	ENSDARP00000011707	uqcrfs1	uqcrfs1-001	Ubiquinol-cytochrome c reductase, Rieske iron-sulfur polypeptide 1	Energy metabolism	PQ INCREASED	3.05
279	ENSDARP00000099928	uqcrfs1	uqcrfs1-201	Ubiquinol-cytochrome c reductase, Rieske iron-sulfur polypeptide 1	Energy metabolism	PQ INCREASED	3.05
280	ENSDARP00000103915	c9	c9-202	Complement component 9	Immune system related	PQ INCREASED	2.95
281	ENSDARP00000121323	c9	c9-001	Complement component 9	Immune system related	PQ INCREASED	2.95
282	ENSDARP00000128894	thop1	thop1-002	Thimet oligopeptidase 1	Protein degradation and synthesis inhibition	PQ INCREASED	2.91
283	ENSDARP00000051373	c9	c9-201	Complement component 9	Immune system related	PQ INCREASED	2.90
284	ENSDARP00000109027	cdk1	cdk1-001	Cyclin-dependent kinase 1	Cell cycle, division, growth and fate	PQ INCREASED	2.90
285	ENSDARP00000054077	rpa2	rpa2-001	Replication protein A2	Cell cycle, division, growth and fate	PQ INCREASED	2.90
286	ENSDARP00000058424	CABZ01059627.1	CABZ01059627.1-201	Mucin 5Ac	Cell cycle, division, growth and fate	PQ INCREASED	2.87
287	ENSDARP00000069494	eif2s2	eif2s2-001	Translation initiation factor 2 subunit 2	Protein Synthesis	PQ INCREASED	2.74
288	ENSDARP00000065372	eef1b2	eef1b2-001	Elongation factor 1-beta	Protein Synthesis	PQ INCREASED	2.72
289	ENSDARP00000009441	pcid2	pcid2-001	PCI domain containing 2	Immune system related	PQ INCREASED	2.62
290	ENSDARP00000116670	nap1l4b	nap1l4b-002	Nucleosome assembly protein 1-like 4b	Cell cycle, division, growth and fate	PQ INCREASED	2.62
291	ENSDARP00000096322	arl3	arl3-001	ADP-ribosylation factor-like 3	Cell cycle, division, growth and fate	PQ INCREASED	2.62
292	ENSDARP00000095451	tpd52l2b	tpd52l2b-002	Tumor protein D52-like 2b	Oncogenes related	PQ INCREASED	2.62
293	ENSDARP00000022360	anp32a	anp32a-001	Acidic (leucine-rich) nuclear phosphoprotein 32 family, member A	Cell cycle, division, growth and fate	PQ INCREASED	2.62
294	ENSDARP00000123639	tcn2l	tcn2l-003	Transcobalamin II	Other	PQ INCREASED	2.62
295	ENSDARP00000112902	srsf1b	srsf1b-002	Serine/arginine-rich splicing factor 1b	Protein Synthesis	PQ INCREASED	2.62
296	ENSDARP00000120687	actr2b	actr2b-002	Actin-related protein 2b homolog	Cell cycle, division, growth and fate	PQ INCREASED	2.62
297	ENSDARP00000107467	snrpa1	snrpa1-002	Small nuclear ribonucleoprotein polypeptide A	Protein Synthesis	PQ INCREASED	2.62
298	ENSDARP00000116615	anp32a	anp32a-002	Acidic (leucine-rich) nuclear phosphoprotein 32 family, member A	Cell cycle, division, growth and fate	PQ INCREASED	2.62
299	ENSDARP00000015887	skp1	skp1-001	S-phase kinase-associated protein 1	Protein degradation and synthesis inhibition	PQ INCREASED	2.62
300	ENSDARP00000115511	tpd52l2b	tpd52l2b-004	Tumor protein D52-like 2b	Oncogenes related	PQ INCREASED	2.62
301	ENSDARP00000060303	dhrs13a.3	dhrs13a.3-001	Dehydrogenase/reductase (SDR family) member 13a, duplicate 3	REDOX/Detox related	PQ INCREASED	2.62
302	ENSDARP00000127050	nup43	nup43-003	Nucleoporin 43	Protein Synthesis	PQ INCREASED	2.62
303	ENSDARP00000117901	tpd52l2b	tpd52l2b-006	Tumor protein D52-like 2b	Oncogenes related	PQ INCREASED	2.62
304	ENSDARP00000105248	ist1	ist1-201	Increased sodium tolerance 1 homolog (yeast)	Protein degradation and synthesis inhibition	PQ INCREASED	2.62
305	ENSDARP00000116690	pus1	pus1-002	Pseudouridylate synthase 1	Protein Synthesis	PQ INCREASED	2.61
306	ENSDARP00000116432	slc25a5	slc25a5-003	Solute carrier family 25 (mitochondrial carrier; adenine nucleotide translocator), member 5	Other	PQ INCREASED	2.61
307	ENSDARP00000059040	h1m	h1m-201	Linker histone 1M	Cell cycle, division, growth and fate	PQ INCREASED	2.61
308	ENSDARP00000104211	myl9b	myl9b-201	Myosin, light chain 9b, regulatory	Cell cycle, division, growth and fate	PQ INCREASED	2.61
309	ENSDARP00000121790	tpd52l2b	tpd52l2b-008	Tumor protein D52-like 2b	Oncogenes related	PQ INCREASED	2.61
310	ENSDARP00000094070	ndufa5	ndufa5-001	NADH dehydrogenase (ubiquinone) 1 alpha subcomplex subunit 5	Energy metabolism	PQ INCREASED	2.61
311	ENSDARP00000023772	marcksb	marcksb-001	Myristoylated alanine-rich C-kinase substrate	Apoptosis related	PQ INCREASED	2.61
312	ENSDARP00000066545	nif3l1	nif3l1-001	NIF3 NGG1 interacting factor 3-like 1	Other	PQ INCREASED	2.61
313	ENSDARP00000117802	tpd52l2b	tpd52l2b-009	Tumor protein D52-like 2b	Oncogenes related	PQ INCREASED	2.61
314	ENSDARP00000116734	ist1	ist1-002	Increased sodium tolerance 1 homolog (yeast)	Protein degradation and synthesis inhibition	PQ INCREASED	2.61
315	ENSDARP00000117716	casp3a	casp3a-002	Aspase 3, apoptosis-related cysteine peptidase a	Apoptosis related	PQ INCREASED	2.61
316	ENSDARP00000068086	ist1	ist1-001	Increased sodium tolerance 1 homolog (yeast)	Protein degradation and synthesis inhibition	PQ INCREASED	2.61
317	ENSDARP00000067511	psma4	psma4-001	Proteasome (prosome, macropain) subunit, alpha type, 4	Protein degradation and synthesis inhibition	PQ INCREASED	2.61
318	ENSDARP00000025342	cox5aa	cox5aa-201	Cytochrome c oxidase subunit 5a	Energy metabolism	PQ INCREASED	2.61
319	ENSDARP00000105687	cox5aa	cox5aa-203	Cytochrome c oxidase subunit 5a	Energy metabolism	PQ INCREASED	2.61
320	ENSDARP00000106612	cox5aa	cox5aa-202	Cytochrome c oxidase subunit 5a	Energy metabolism	PQ INCREASED	2.61
321	ENSDARP00000123538	cox5aa	cox5aa-001	Cytochrome c oxidase subunit 5a	Energy metabolism	PQ INCREASED	2.61
322	ENSDARP00000051185	nccrp1	nccrp1-001	Non-specific cytotoxic cell receptor protein 1 homolog (zebrafish)	Immune system related	PQ INCREASED	2.61
323	ENSDARP00000073363	fdx1	fdx1-005	Ferredoxin 1	REDOX/Detox related	PQ INCREASED	2.61
324	ENSDARP00000113657	fdx1	fdx1-002	Ferredoxin 1	REDOX/Detox related	PQ INCREASED	2.61
325	ENSDARP00000124949	fdx1	fdx1-004	Ferredoxin 1	REDOX/Detox related	PQ INCREASED	2.61
326	ENSDARP00000064438	nup43	nup43-001	Nucleoporin 43	Protein Synthesis	PQ INCREASED	2.61
327	ENSDARP00000069298	si:ch73-207h17.1	si:ch73-207h17.1-201	Uncharacterized protein	Other	PQ INCREASED	2.61
328	ENSDARP00000121841	tpd52l2b	tpd52l2b-007	Tumor protein D52-like 2b	Oncogenes related	PQ INCREASED	2.61
329	ENSDARP00000003222	cyp20a1	cyp20a1-001	Cytochrome P450, family 20, subfamily A, polypeptide 1	REDOX/Detox related	PQ INCREASED	2.61
330	ENSDARP00000097644	anp32a	anp32a-201	Anp32a-001	Cell cycle, division, growth and fate	PQ INCREASED	2.61
331	ENSDARP00000129796	ap2a1	ap2a1-003	Adaptor-related protein complex 2, alpha 1 subunit	Protein degradation and synthesis inhibition	PQ INCREASED	2.61

332	ENSDARP00000051197	mis18a	mis18a-001	MIS18 kinetochore protein A	Cell cycle, division, growth and fate	PQ INCREASED	2.61
333	ENSDARP00000003173	seh1l	seh1l-001	Nucleoporin SEH1	Cell cycle, division, growth and fate	PQ INCREASED	2.61
334	ENSDARP00000040239	tiprl	tiprl-201	TIP41, TOR signaling pathway regulator-like (S. cerevisiae)	Cell cycle, division, growth and fate	PQ INCREASED	2.61
335	ENSDARP00000023199	tpd52l2b	tpd52l2b-001	Tumor protein D52-like 2b	Oncogenes related	PQ INCREASED	2.61
336	ENSDARP00000104624	tpd52l2b	tpd52l2b-201	Tumor protein D52-like 2b	Oncogenes related	PQ INCREASED	2.61
337	ENSDARP00000045554	zgc:63637	zgc:63637-001	RAB41, member RAS oncogene family [	Oncogenes related	PQ INCREASED	2.60
338	ENSDARP00000110405	zgc:63637	zgc:63637-201	RAB41, member RAS oncogene family [	Oncogenes related	PQ INCREASED	2.60
339	ENSDARP00000095078	actl6a	actl6a-001	Actin-like 6A	Cell cycle, division, growth and fate	PQ INCREASED	2.60
340	ENSDARP00000015934	rragd	rragd-001	Ras-related GTP binding D	Apoptosis related	PQ INCREASED	2.60
341	ENSDARP00000044837	ndrg1a	ndrg1a-001	N-myc downstream regulated 1a	Cell cycle, division, growth and fate	PQ INCREASED	2.49
342	ENSDARP00000027276	eif4a3	eif4a3-001	Eukaryotic translation initiation factor 4A3	Protein Synthesis	PQ INCREASED	2.43
343	ENSDARP00000039951	idh3a	idh3a-201	Isocitrate dehydrogenase 3 (NAD+) alpha	Energy metabolism	PQ INCREASED	2.40
344	ENSDARP00000105892	ddost	ddost-001	Dolichyl-diphosphooligosaccharide--protein glycosyltransferase subunit (non-catalytic	Protein Synthesis	PQ INCREASED	2.39
345	ENSDARP00000121212	myl6	myl6-004	Myosin light chain 6	Cell cycle, division, growth and fate	PQ INCREASED	2.37
346	ENSDARP00000033600	rplp1	rplp1-001	Ribosomal protein, large, P1	Protein Synthesis	PQ INCREASED	2.36
347	ENSDARP00000075666	krt5	krt5-201	Keratin 5	Cell cycle, division, growth and fate	PQ INCREASED	2.33
348	ENSDARP00000079733	l2hgdh	l2hgdh-001	L-2-hydroxyglutarate dehydrogenase [	REDOX/Detox related	PQ INCREASED	2.33
349	ENSDARP00000092718	rragca	rragca-001	Ras-related GTP binding Ca	Cell cycle, division, growth and fate	PQ INCREASED	2.32
350	ENSDARP00000107677	ppid	ppid-201	Peptidylprolyl isomerase D	Protein Synthesis	PQ INCREASED	2.32
351	ENSDARP00000108711	krt5	krt5-001	Keratin 5	Cell cycle, division, growth and fate	PQ INCREASED	2.32
352	ENSDARP00000016453	krt4	krt4-001	Keratin 4	Cell cycle, division, growth and fate	PQ INCREASED	2.32
353	ENSDARP00000065350	krt4	krt4-201	Keratin 4	Cell cycle, division, growth and fate	PQ INCREASED	2.32
354	ENSDARP00000126991	nif3l1	nif3l1-002	NIF3 NGG1 interacting factor 3-like 1	Other	PQ INCREASED	2.32
355	ENSDARP00000061743	zp3a.1	zp3a.1-001	zona pellucida glycoprotein 3a, tandem duplicate 1	Zona Pellucida proteins	PQ INCREASED	2.27
356	ENSDARP00000103385	ppp4cb	ppp4cb-001	Protein phosphatase 4, catalytic subunit b	Protein degradation and synthesis inhibition	PQ INCREASED	2.23
357	ENSDARP00000093277	anp32b	anp32b-001	Acidic (leucine-rich) nuclear phosphoprotein 32 family, member B	Cell cycle, division, growth and fate	PQ INCREASED	2.18
358	ENSDARP00000055503	cyc1	cyc1-001	Ubiquinol-cytochrome c reductase cytochrome c1 subunit	Energy metabolism	PQ INCREASED	2.18
359	ENSDARP00000075074	arpc3	arpc3-001	Actin related protein 2/3 complex, subunit 3	Apoptosis related	PQ INCREASED	2.18
360	ENSDARP00000094142	ociad1	ociad1-001	OCIA domain containing 2	Endosome-Lysosome related	PQ INCREASED	2.18
361	ENSDARP00000111978	ociad1	ociad1-201	OCIA domain containing 2	Endosome-Lysosome related	PQ INCREASED	2.18
362	ENSDARP00000112683	ociad1	ociad1-002	OCIA domain containing 2	Endosome-Lysosome related	PQ INCREASED	2.18
363	ENSDARP00000039222	gnpda2	gnpda2-201	Glucosamine-6-phosphate deaminase 2	Energy metabolism	PQ INCREASED	2.18
364	ENSDARP00000114418	gnpda2	gnpda2-001	Glucosamine-6-phosphate deaminase 2	Energy metabolism	PQ INCREASED	2.18
365	ENSDARP00000069977	rpl23	rpl23-001	Ribosomal protein L23	Protein Synthesis	PQ INCREASED	2.18
366	ENSDARP00000109902	zgc:162816	zgc:162816-201	D-serine dehydratase-like	Other	PQ INCREASED	2.18
367	ENSDARP00000117083	tomm70a	tomm70a-001	Translocase of outer mitochondrial membrane 70 homolog A (S. cerevisiae)	Other	PQ INCREASED	2.18
368	ENSDARP00000011426	ambp	ambp-001	Arginyl aminopeptidase (aminopeptidase B)	Protein degradation and synthesis inhibition	PQ INCREASED	2.18
369	ENSDARP00000098620	mvda	mvda-001	Mevalonate (diphospho) decarboxylase a	Lipid metabolism	PQ INCREASED	2.18
370	ENSDARP00000127428	rrm2	rrm2-004	Ribonucleotide reductase M2 polypeptide	Cell cycle, division, growth and fate	PQ INCREASED	2.18
371	ENSDARP00000107514	fkbp3	fkbp3-202	FK506 binding protein 3	Protein Synthesis	PQ INCREASED	2.17
372	ENSDARP00000008018	map2k1	map2k1-001	Mitogen-activated protein kinase kinase 1	Apoptosis related	PQ INCREASED	2.17
373	ENSDARP00000027385	hnrnph1l	hnrnph1l-001	Heterogeneous nuclear ribonucleoprotein H1, like	Protein Synthesis	PQ INCREASED	2.17
374	ENSDARP00000128433	serpinb1l3	serpinb1l3-002	Serpin peptidase inhibitor, clade B (ovalbumin), member 1, like 3	Other	PQ INCREASED	2.12
375	ENSDARP00000014914	map2k2a	map2k2a-001	Mitogen-activated protein kinase kinase 2a	Apoptosis related	PQ INCREASED	2.12
376	ENSDARP00000059346	bckdha	bckdha-001	Branched chain keto acid dehydrogenase E1, alpha polypeptide	Protein degradation and synthesis inhibition	PQ INCREASED	2.12
377	ENSDARP00000106474	bckdha	bckdha-201	Branched chain keto acid dehydrogenase E1, alpha polypeptide	Protein degradation and synthesis inhibition	PQ INCREASED	2.12
378	ENSDARP00000093670	ndrg1a	ndrg1a-002	N-myc downstream regulated gene 1a	Cell cycle, division, growth and fate	PQ INCREASED	2.12
379	ENSDARP00000047263	eef1db	eef1db-202	Elongation factor-1, delta, b	Protein Synthesis	PQ INCREASED	2.09
380	ENSDARP00000129723	thop1	thop1-003	Thimet oligopeptidase 1	Protein degradation and synthesis inhibition	PQ INCREASED	2.09
381	ENSDARP00000109129	si:ch211-11k18.4	si:ch211-11k18.4-201	Uncharacterized protein	Other	PQ INCREASED	2.09
382	ENSDARP00000123562	si:ch211-11k18.4	si:ch211-11k18.4-001	Uncharacterized protein	Other	PQ INCREASED	2.09
383	ENSDARP00000100592	eef1db	eef1db-001	Elongation factor-1, delta, b	Protein Synthesis	PQ INCREASED	2.08
384	ENSDARP00000029821	eef1db	eef1db-201	Elongation factor-1, delta, b	Protein Synthesis	PQ INCREASED	2.08
385	ENSDARP00000104512	hsp90ab1	hsp90ab1-201	Heat shock protein 90kDa alpha (cytosolic), class B member 1	Protein Synthesis	PQ INCREASED	2.08
386	ENSDARP00000014978	hsp90ab1	hsp90ab1-001	Heat shock protein 90kDa alpha (cytosolic), class B member 1	Protein Synthesis	PQ INCREASED	2.08
387	ENSDARP00000042703	eef1db	eef1db-002	Elongation factor-1, delta, b	Protein Synthesis	PQ INCREASED	2.08
388	ENSDARP00000070780	pcna	pcna-001	Proliferating cell nuclear antigen	Cell cycle, division, growth and fate	PQ INCREASED	2.07
389	ENSDARP00000022789	bzw1a	bzw1a-001	Basic leucine zipper and W2 domains 1a	Cell cycle, division, growth and fate	PQ INCREASED	2.07
390	ENSDARP00000038550	mapk1	mapk1-001	Mitogen-activated protein kinase kinase 2a	Apoptosis related	PQ INCREASED	2.03
391	ENSDARP00000123916	mapk1	mapk1-002	Mitogen-activated protein kinase kinase 2a	Apoptosis related	PQ INCREASED	2.03
392	ENSDARP00000076624	rragcb	rragcb-001	Ras-related GTP binding Ca	Cell cycle, division, growth and fate	PQ INCREASED	2.03
393	ENSDARP00000064746	stoml2	stoml2-201	Stomatin (EPB72)-like 2	Other	PQ INCREASED	2.03
394	ENSDARP00000124593	idh3b	idh3b-001	Isocitrate dehydrogenase 3 (NAD+) beta	Energy metabolism	PQ INCREASED	2.03
395	ENSDARP00000048382	creld2	creld2-001	Cysteine-rich with EGF-like domains 2	Other	PQ INCREASED	2.03
396	ENSDARP00000109480	vtg5	vtg5-201	Vitellogenin 5	Vitellogenins	PQ INCREASED	2.03
397	ENSDARP00000123659	tpi1b	tpi1b-002	Triosephosphate isomerase 1b	Energy metabolism	PQ INCREASED	2.00
398	ENSDARP00000113319	eif3ea	eif3ea-003	Translation initiation factor 3 subunit E	Protein Synthesis	GQ INCREASED	0.50
399	ENSDARP00000037758	cotl1	cotl1-001	Coactosin-like 1 (Dictyostelium)	Cell cycle, division, growth and fate	GQ INCREASED	0.50

400	ENSDARP00000022310	adsl	adsl-001	Adenylosuccinate lyase	Cell cycle, division, growth and fate	GQ INCREASED	0.50
401	ENSDARP00000007588	pgd	pgd-001	Phosphogluconate hydrogenase	Energy metabolism	GQ INCREASED	0.50
402	ENSDARP00000014732	cyb5r3	cyb5r3-001	Cytochrome b5 reductase 3	Energy metabolism	GQ INCREASED	0.50
403	ENSDARP00000097681	prps1a	prps1a-002	Phosphoribosyl pyrophosphate synthetase 1A	Protein Synthesis	GQ INCREASED	0.50
404	ENSDARP00000009909	gpia	gpia-001	Glucose phosphate isomerase a	Energy metabolism	GQ INCREASED	0.50
405	ENSDARP00000003107	cct7	cct7-001	Chaperonin containing TCP1, subunit 7	Protein Synthesis	GQ INCREASED	0.50
406	ENSDARP00000123779	fasn	fasn-001	Fatty acid synthase	Lipid metabolism	GQ INCREASED	0.50
407	ENSDARP00000017693	ppp2r1b	ppp2r1b-001	Protein phosphatase 2	Protein Synthesis	GQ INCREASED	0.50
408	ENSDARP00000127789	si:ch211-157j23.3	si:ch211-157j23.3-202	NXPE family member 3-like	Other	GQ INCREASED	0.50
409	ENSDARP00000114435	si:dkey-105h11.2	si:dkey-105h11.2-001	NXPE family member 3-like	Other	GQ INCREASED	0.50
410	ENSDARP00000058383	gapdhs	gapdhs-001	Glyceraldehyde-3-phosphate dehydrogenase, spermatogenic	Energy metabolism	GQ INCREASED	0.50
411	ENSDARP00000093927	FBXO2	FBXO2-201	F-box protein 2	Protein degradation and synthesis inhibition	GQ INCREASED	0.50
412	ENSDARP00000018064	acat2	acat2-001	Acetyl-CoA acetyltransferase 2	Energy metabolism	GQ INCREASED	0.50
413	ENSDARP00000108188	ssr1	ssr1-201	Translocon-associated protein subunit alpha	Protein Synthesis	GQ INCREASED	0.50
414	ENSDARP00000012454	phc2b	phc2b-201	Polyhomeotic-like protein 2-Histone H2A	Cell cycle, division, growth and fate	GQ INCREASED	0.50
415	ENSDARP00000029067	rpa1	rpa1-001	Replication protein A1	Cell cycle, division, growth and fate	GQ INCREASED	0.50
416	ENSDARP00000093898	p4hb	p4hb-001	Procollagen-proline, 2-oxoglutarate 4-dioxygenase (proline 4-hydroxylase), beta polypeptide	Protein Synthesis	GQ INCREASED	0.50
417	ENSDARP00000104575	tcp1	tcp1-201	T-complex polypeptide 1	Protein Synthesis	GQ INCREASED	0.50
418	ENSDARP00000034904	psmc4	psmc4-001	Proteasome (prosome, macropain) 26S subunit, ATPase, 4	Protein Synthesis	GQ INCREASED	0.50
419	ENSDARP00000113985	si:ch211-251f6.7	si:ch211-251f6.7-001	Fish egg lectin like	Lectins	GQ INCREASED	0.50
420	ENSDARP00000009285	napa	napa-001	N-ethylmaleimide-sensitive factor attachment protein, alpha	Other	GQ INCREASED	0.50
421	ENSDARP00000048990	si:ch211-157j23.2	si:ch211-157j23.2-001	NXPE family member 3-like	Other	GQ INCREASED	0.50
422	ENSDARP00000129506	eprs	eprs-001	Glutamyl-prolyl-tRNA synthetase	Protein Synthesis	GQ INCREASED	0.49
423	ENSDARP00000125075	sept7b	Novel	Cytoskeleton proteins	Cell cycle, division, growth and fate	GQ INCREASED	0.49
424	ENSDARP00000112912	impa1	impa1-002	Inositol(myo)-1(or 4)-monophosphatase 1	Cell cycle, division, growth and fate	GQ INCREASED	0.49
425	ENSDARP00000076390	rtn4a	rtn4a-003	Reticulon 4a	Protein Synthesis	GQ INCREASED	0.49
426	ENSDARP00000079227	prosc	prosc-201	Proline synthetase co-transcribed homolog (bacterial)	Protein Synthesis	GQ INCREASED	0.49
427	ENSDARP00000116169	prosc	prosc-001	Proline synthetase co-transcribed homolog (bacterial)	Protein Synthesis	GQ INCREASED	0.49
428	ENSDARP00000018737	rpl27	rpl27-001	Arge subunit ribosomal protein L27e	Protein Synthesis	GQ INCREASED	0.49
429	ENSDARP00000117864	rpl27	rpl27-002	Arge subunit ribosomal protein L27e	Protein Synthesis	GQ INCREASED	0.49
430	ENSDARP00000072525	C20H6orf211	C20H6orf211-001	Protein-glutamate O-methyltransferase	Cell cycle, division, growth and fate	GQ INCREASED	0.49
431	ENSDARP00000066897	rps16	rps16-001	Ribosomal protein S16	Protein Synthesis	GQ INCREASED	0.49
432	ENSDARP00000089806	si:ch211-251f6.7	si:ch211-251f6.7-201	Fish egg lectin like	Lectins	GQ INCREASED	0.49
433	ENSDARP00000075671	abhd14a	abhd14a-001	Abhydrolase domain containing 14A	Protein degradation and synthesis inhibition	GQ INCREASED	0.49
434	ENSDARP00000109466	abhd14a	abhd14a-201	Abhydrolase domain containing 14A	Protein degradation and synthesis inhibition	GQ INCREASED	0.49
435	ENSDARP00000028641	dnaja2	dnaja2-201	DnaJ homolog subfamily A member 2	Protein Synthesis	GQ INCREASED	0.49
436	ENSDARP00000045893	rnaseh2a	rnaseh2a-001	Ribonuclease H2 subunit A	Cell cycle, division, growth and fate	GQ INCREASED	0.49
437	ENSDARP00000108257	sept2-	sept2-001	Septin 2	Cell cycle, division, growth and fate	GQ INCREASED	0.49
438	ENSDARP00000105058	sept2-	sept2-203	Septin 2	Cell cycle, division, growth and fate	GQ INCREASED	0.49
439	ENSDARP00000056965	sept2-	sept2-201	Septin 2	Cell cycle, division, growth and fate	GQ INCREASED	0.49
440	ENSDARP00000105338	sept2-	sept2-202	Septin 2	Cell cycle, division, growth and fate	GQ INCREASED	0.49
441	ENSDARP00000046972	capza1a	capza1a-201	Capping protein (actin filament) muscle Z-line, alpha 1a	Cell cycle, division, growth and fate	GQ INCREASED	0.49
442	ENSDARP00000062644	scarb1	scarb1-201	Scavenger receptor class B, member 1	Endosome-Lysosome related	GQ INCREASED	0.49
443	ENSDARP00000104653	eif4a2	eif4a2-001	Eukaryotic translation initiation factor 4A, isoform 1B	Protein Synthesis	GQ INCREASED	0.49
444	ENSDARP00000070224	eprs	eprs-201	Glutamyl-prolyl-tRNA synthetase	Protein Synthesis	GQ INCREASED	0.49
445	ENSDARP00000121669	dctn2	dctn2-001	Dynactin 2	Cell cycle, division, growth and fate	GQ INCREASED	0.49
446	ENSDARP00000093918	dctn2	dctn2-202	Dynactin 2	Cell cycle, division, growth and fate	GQ INCREASED	0.49
447	ENSDARP00000127771	zgc:152830	zgc:152830-001	Peptidase M17 superfamily-	Protein degradation and synthesis inhibition	GQ INCREASED	0.49
448	ENSDARP00000097937	zgc:152830	zgc:152830-001	Cytosol aminopeptidase family	Protein degradation and synthesis inhibition	GQ INCREASED	0.49
449	ENSDARP00000119229	si:ch211-157j23.5	si:ch211-157j23.5-201	NXPE family member 3-like	Other	GQ INCREASED	0.49
450	ENSDARP00000129574	si:ch211-157j23.5	si:ch211-157j23.5-001	NXPE family member 3-like	Other	GQ INCREASED	0.49
451	ENSDARP00000057943	rpl26	rpl26-001	Ribosomal protein L26	Protein Synthesis	GQ INCREASED	0.49
452	ENSDARP00000126732	rpl24	rpl24-001	Ribosomal protein L24	Protein Synthesis	GQ INCREASED	0.49
453	ENSDARP00000105266	zgc:152830	zgc:152830-201	Peptidase M17 superfamily-	Protein degradation and synthesis inhibition	GQ INCREASED	0.48
454	ENSDARP00000103413	vars	vars-202	Valyl-tRNA synthetase	Protein Synthesis	GQ INCREASED	0.48
455	ENSDARP00000094372	npc1	npc1-001	Niemann-Pick disease, type C1	Endosome-Lysosome related	GQ INCREASED	0.48
456	ENSDARP00000059310	siae	siae-001	Sialic acid acetylesterase	Immune system related	GQ INCREASED	0.48
457	ENSDARP00000042594	dctn2	dctn2-201	Dynactin 2	Cell cycle, division, growth and fate	GQ INCREASED	0.48
458	ENSDARP00000124582	cnp	cnp-001	2',3'-cyclic nucleotide 3' phosphodiesterase	Cell cycle, division, growth and fate	GQ INCREASED	0.48
459	ENSDARP00000061100	igf2r	igf2r-001	Insulin-like growth factor 2 receptor	Endosome-Lysosome related	GQ INCREASED	0.48
460	ENSDARP00000129036	si:dkey-241l7.6	si:dkey-241l7.6-201	C-type lectin	Lectins	GQ INCREASED	0.48
461	ENSDARP00000060465	si:dkey-241l7.6	si:dkey-241l7.6-001	C-type lectin	Lectins	GQ INCREASED	0.48
462	ENSDARP00000127254	si:dkey-241l7.6	si:dkey-241l7.6-003	C-type lectin	Lectins	GQ INCREASED	0.48
463	ENSDARP00000006510	pgm1	pgm1-001	Phosphoglucomutase 1	Energy metabolism	GQ INCREASED	0.48
464	ENSDARP00000092429	calrl2	calrl2-001	Calreticulin, like 2	Protein Synthesis	GQ INCREASED	0.48
465	ENSDARP00000096983	ids	ids-001	Iduronate 2-sulfatase	Endosome-Lysosome related	GQ INCREASED	0.48
466	ENSDARP00000090432	zgc:55413	zgc:55413-202	Palmitoyl-(protein) hydrolase activity	Other	GQ INCREASED	0.48
467	ENSDARP00000125995	pkma	pkma-002	Pyruvate kinase, muscle, a		GQ INCREASED	0.48

468	ENSDARP00000125765	pkma	pkma-001	Pyruvate kinase, muscle, a	Energy metabolism	GQ INCREASED	0.48
469	ENSDARP00000099755	psmb5	psmb5-001	Proteasome (prosome, macropain) subunit, beta type, 5	Protein degradation and synthesis inhibition	GQ INCREASED	0.48
470	ENSDARP00000020154	ehd3	ehd3-001	EH-domain containing 3	Protein degradation and synthesis inhibition	GQ INCREASED	0.48
471	ENSDARP00000074719	pgam2	pgam2-001	Phosphoglycerate mutase 2 (muscle	Energy metabolism	GQ INCREASED	0.48
472	ENSDARP00000004738	cct3	cct3-001	Chaperonin containing Tcp1, subunit 3	Protein Synthesis	GQ INCREASED	0.48
473	ENSDARP00000052929	ndufv1	ndufv1-001	NADH dehydrogenase (ubiquinone) flavoprotein 1	Energy metabolism	GQ INCREASED	0.47
474	ENSDARP00000087338	ppp1cab	ppp1cab-001	Protein phosphatase 1, catalytic subunit, alpha isoform b	Cell cycle, division, growth and fate	GQ INCREASED	0.47
475	ENSDARP00000019120	anxa11b	anxa11b-201	Annexin A11b	Immune system related	GQ INCREASED	0.47
476	ENSDARP00000124670	anxa11b	anxa11b-001	Annexin A11b	Immune system related	GQ INCREASED	0.47
477	ENSDARP00000112654	eif5a	eif5a-003	Eukaryotic translation initiation factor 5A	Protein Synthesis	GQ INCREASED	0.47
478	ENSDARP00000124216	cnp	cnp-007	2',3'-cyclic nucleotide 3' phosphodiesterase	Cell cycle, division, growth and fate	GQ INCREASED	0.47
479	ENSDARP00000112576	zgc:66313	zgc:66313-001	Alpha-amylase	Energy metabolism	GQ INCREASED	0.47
480	ENSDARP00000119679	cnp	cnp-010	2',3'-cyclic nucleotide 3' phosphodiesterase	Cell cycle, division, growth and fate	GQ INCREASED	0.47
481	ENSDARP00000127543	si:ch211-157j23.2	si:ch211-157j23.2-201	NXPE family member 3-like	Other	GQ INCREASED	0.47
482	ENSDARP00000062650	pkma	pkma-201	Pyruvate kinase, muscle, a	Energy metabolism	GQ INCREASED	0.47
483	ENSDARP00000069223	shmt1	shmt1-001	Serine hydroxymethyltransferase 1 (soluble)	Protein Synthesis	GQ INCREASED	0.46
484	ENSDARP00000120541	cnp	cnp-003	2',3'-cyclic nucleotide 3' phosphodiesterase	Cell cycle, division, growth and fate	GQ INCREASED	0.46
485	ENSDARP00000110339	pkma	pkma-203	Pyruvate kinase, muscle, a	Energy metabolism	GQ INCREASED	0.46
486	ENSDARP00000091365	pdia5	pdia5-001	Protein disulfide isomerase family A, member 5	Protein Synthesis	GQ INCREASED	0.46
487	ENSDARP00000108306	cdc42	cdc42-202	Cell division cycle 42	Cell cycle, division, growth and fate	GQ INCREASED	0.46
488	ENSDARP00000116065	zgc:66313	zgc:66313-007	Alpha-amylase	Energy metabolism	GQ INCREASED	0.46
489	ENSDARP00000015775	sars	sars-001	Seryl-aminoacyl-tRNA synthetase	Protein Synthesis	GQ INCREASED	0.46
490	ENSDARP00000114417	cnp	cnp-004	2',3'-cyclic nucleotide 3' phosphodiesterase	Cell cycle, division, growth and fate	GQ INCREASED	0.46
491	ENSDARP00000117465	cnp	cnp-009	2',3'-cyclic nucleotide 3' phosphodiesterase	Cell cycle, division, growth and fate	GQ INCREASED	0.46
492	ENSDARP00000118406	cnp	cnp-005	2',3'-cyclic nucleotide 3' phosphodiesterase	Cell cycle, division, growth and fate	GQ INCREASED	0.45
493	ENSDARP00000118615	zgc:66313	zgc:66313-003	Alpha-amylase	Energy metabolism	GQ INCREASED	0.45
494	ENSDARP00000112608	cct6a	cct6a-002	Chaperonin containing TCP1, subunit 6A	Protein Synthesis	GQ INCREASED	0.45
495	ENSDARP00000027102	nap1l1	nap1l1-201	Nucleosome assembly protein 1-like 1	Cell cycle, division, growth and fate	GQ INCREASED	0.45
496	ENSDARP00000003468	cct8	cct8-001	T-complex protein 1 subunit theta	Protein Synthesis	GQ INCREASED	0.45
497	ENSDARP00000107134	ktn1	ktn1-001	Kinectin 1	Other	GQ INCREASED	0.44
498	ENSDARP00000120480	zgc:66313	zgc:66313-002	Alpha-amylase	Energy metabolism	GQ INCREASED	0.44
499	ENSDARP00000122652	zgc:66313	zgc:66313-005	Alpha-amylase	Energy metabolism	GQ INCREASED	0.44
500	ENSDARP00000082159	igf2bp1	igf2bp1-001	Insulin-like growth factor 2 mRNA binding protein 1	Protein Synthesis	GQ INCREASED	0.44
501	ENSDARP00000005691	ktn1	ktn1-201	Kinectin 1	Other	GQ INCREASED	0.44
502	ENSDARP00000020770	mthfd1b	mthfd1b-001	Methylenetetrahydrofolate dehydrogenase (NADP+ dependent) 1	Cell cycle, division, growth and fate	GQ INCREASED	0.44
503	ENSDARP00000102463	sept8a	sept8a-001	Septin 8a	Cell cycle, division, growth and fate	GQ INCREASED	0.44
504	ENSDARP00000056720	ldhd	ldhd-001	D-lactate dehydrogenase (cytochrome)	Energy metabolism	GQ INCREASED	0.44
505	ENSDARP00000125746	neu3.4	neu3.4-001	Sialidase 3, tandem duplicate 4	Lipid metabolism	GQ INCREASED	0.44
506	ENSDARP00000037906	usp14	usp14-201	Ubiquitin specific peptidase 14	Protein degradation and synthesis inhibition	GQ INCREASED	0.44
507	ENSDARP00000127023	si:ch1073-13h15.3	si:ch1073-13h15.3-001	Putative all-trans-retinol 13,14-reductase	REDOX/Detox related	GQ INCREASED	0.44
508	ENSDARP00000011673	ddi2	ddi2-001	DNA-damage inducible protein 2	Protein degradation and synthesis inhibition	GQ INCREASED	0.44
509	ENSDARP00000013512	zgc:112334	zgc:112334-201	Rab GDP dissociation inhibitor alpha	Other	GQ INCREASED	0.44
510	ENSDARP00000049225	acox1	acox1-001	Acyl-CoA oxidase 1, palmitoyl	Lipid metabolism	GQ INCREASED	0.44
511	ENSDARP00000067559	acox1	acox1-002	Acyl-CoA oxidase 1, palmitoyl	Lipid metabolism	GQ INCREASED	0.44
512	ENSDARP00000017123	glud1b	glud1b-201	Glutamate dehydrogenase 1b	Energy metabolism	GQ INCREASED	0.44
513	ENSDARP00000091816	glud1b	glud1b-001	Glutamate dehydrogenase 1b	Energy metabolism	GQ INCREASED	0.44
514	ENSDARP00000024643	adh8a	adh8a-001	Alcohol dehydrogenase 8a	REDOX/Detox related	GQ INCREASED	0.44
515	ENSDARP00000090576	arpc2	arpc2-001	Actin related protein 2/3 complex, subunit 2	Apoptosis related	GQ INCREASED	0.44
516	ENSDARP00000111384	zgc:112334	zgc:112334-202	Rab GDP dissociation inhibitor alpha	Other	GQ INCREASED	0.44
517	ENSDARP00000069585	carhsp1	carhsp1-201	Calcium regulated heat stable protein 1	Protein Synthesis	GQ INCREASED	0.44
518	ENSDARP00000125927	carhsp1	carhsp1-001	Calcium regulated heat stable protein 1	Protein Synthesis	GQ INCREASED	0.44
519	ENSDARP00000064111	glrx5	glrx5-001	Monothiol glutaredoxin	Protein Synthesis	GQ INCREASED	0.44
520	ENSDARP00000041446	ktn1	ktn1-002	Kinectin 1	Other	GQ INCREASED	0.44
521	ENSDARP00000080584	ktn1	ktn1-202	Kinectin 1	Other	GQ INCREASED	0.44
522	ENSDARP00000050670	rap1aa	rap1aa-002	Rap-1A-member of RAS oncogene family a	Oncogenes related	GQ INCREASED	0.44
523	ENSDARP00000116523	rap1aa	rap1aa-001	Rap-1A-member of RAS oncogene family a	Oncogenes related	GQ INCREASED	0.44
524	ENSDARP00000112718	cryz	cryz-002	Crystallin, zeta (quinone reductase)	Energy metabolism	GQ INCREASED	0.44
525	ENSDARP00000006973	dlst	dlst-001	Dihydrolipoamide S-succinyltransferase	Energy metabolism	GQ INCREASED	0.44
526	ENSDARP00000067865	RPS17	RPS17-201	Ribosomal protein S17	Protein Synthesis	GQ INCREASED	0.44
527	ENSDARP00000129150	RPS17	RPS17-001	Ribosomal protein S17	Protein Synthesis	GQ INCREASED	0.44
528	ENSDARP00000095998	CABZ01034848.1	CABZ01034848.1-201	Myosin 9-like	Energy metabolism	GQ INCREASED	0.44
529	ENSDARP00000003030	hibadhb	hibadhb-001	3-hydroxyisobutyrate dehydrogenase b	Protein degradation and synthesis inhibition	GQ INCREASED	0.44
530	ENSDARP00000011148	rps6	rps6-201	Small subunit ribosomal protein S6e	Protein Synthesis	GQ INCREASED	0.44
531	ENSDARP00000093469	ppp2r5d	ppp2r5d-201	Protein phosphatase 2, regulatory subunit B', delta	Protein Synthesis	GQ INCREASED	0.44
532	ENSDARP00000024354	impdh2	impdh2-001	Inosine 5'-monophosphate dehydrogenase 2	Cell cycle, division, growth and fate	GQ INCREASED	0.44
533	ENSDARP00000125879	neu3.5	neu3.5-001	Sialidase 3, tandem duplicate 4	Lipid metabolism	GQ INCREASED	0.44
534	ENSDARP00000126188	rps18	rps18-006	Small subunit ribosomal protein S18e	Protein Synthesis	GQ INCREASED	0.44
535	ENSDARP00000095645	trap1	trap1-201	TNF receptor-associated protein 1	Protein Synthesis	GQ INCREASED	0.44

536	ENSDARP00000095167	smx5	smx5-201	U6 snRNA-associated Sm-like protein LSm2	Protein Synthesis	GQ INCREASED	0.44
537	ENSDARP00000127825	rps28	rps28-004	Small subunit ribosomal protein S28e	Protein Synthesis	GQ INCREASED	0.44
538	ENSDARP00000001565	echs1	echs1-001	Noyl CoA hydratase, short chain, 1, mitochondrial	Lipid metabolism	GQ INCREASED	0.44
539	ENSDARP00000121906	pcca	pcca-002	Propionyl CoA carboxylase, alpha polypeptide	Energy metabolism	GQ INCREASED	0.44
540	ENSDARP00000107200	si:dkey-179j5.5	si:dkey-179j5.5-202	Gal lectin superfamily	Lectins	GQ INCREASED	0.44
541	ENSDARP00000107323	trap1	trap1-202	TNF receptor-associated protein 1	Protein Synthesis	GQ INCREASED	0.44
542	ENSDARP00000052062	rps28	rps28-003	Small subunit ribosomal protein S28e	Protein Synthesis	GQ INCREASED	0.44
543	ENSDARP00000115796	rps28	rps28-001	Small subunit ribosomal protein S28e	Protein Synthesis	GQ INCREASED	0.44
544	ENSDARP00000072253	serpinb1l2	serpinb1l2-001	Serpin peptidase inhibitor, clade B (ovalbumin), member 1, like 2	Other	GQ INCREASED	0.44
545	ENSDARP00000093527	si:dkey-179j5.5	si:dkey-179j5.5-201	Gal lectin superfamily	Lectins	GQ INCREASED	0.44
546	ENSDARP00000114428	cpt2	cpt2-002	Carnitine palmitoyltransferase II	Lipid metabolism	GQ INCREASED	0.44
547	ENSDARP00000121781	myh11a	myh11a-002	Myosin, heavy chain 11a, smooth muscle	Cell cycle, division, growth and fate	GQ INCREASED	0.44
548	ENSDARP00000047012	srprb	srprb-001	Signal recognition particle receptor, B subunit	Protein Synthesis	GQ INCREASED	0.44
549	ENSDARP00000112598	si:ch211-157j23.3	si:ch211-157j23.3-001	NXPE family member 3-like	Other	GQ INCREASED	0.44
550	ENSDARP00000103437	cpdb	cpdb-201	Carboxypeptidase D, b	Protein degradation and synthesis inhibition	GQ INCREASED	0.44
551	ENSDARP00000107538	CABZ01073085.1	CABZ01073085.1-201	Chaperonin containing TCP1, subunit 2 (beta)	Protein Synthesis	GQ INCREASED	0.44
552	ENSDARP00000115288	ufd1l	ufd1l-004	Ubiquitin fusion degradation 1-like	Protein degradation and synthesis inhibition	GQ INCREASED	0.44
553	ENSDARP00000007716	atp5l	atp5l-001	ATP synthase, H+ transporting, mitochondrial F0 complex, subunit g	Energy metabolism	GQ INCREASED	0.44
554	ENSDARP00000126736	ca15b	ca15b-005	Carbonic anhydrase XV b	Energy metabolism	GQ INCREASED	0.44
555	ENSDARP00000025042	sub1b	sub1b-001	UB1 homolog b (S. cerevisiae)	Protein Synthesis	GQ INCREASED	0.44
556	ENSDARP00000117668	man2b1	man2b1-002	Mannosidase, alpha, class 2B, member 1	Energy metabolism	GQ INCREASED	0.44
557	ENSDARP00000118860	si:ch1073-75o15.4	si:ch1073-75o15.4-001	SUEL type lectin	Lectins	GQ INCREASED	0.44
558	ENSDARP00000113030	trap1	trap1-001	TNF receptor-associated protein 1	Protein Synthesis	GQ INCREASED	0.44
559	ENSDARP00000121416	si:ch1073-75o15.4	si:ch1073-75o15.4-002	SUEL type lectin	Lectins	GQ INCREASED	0.44
560	ENSDARP00000075627	neu3.3	neu3.3-001	Sialidase 3, tandem duplicate 4	Lipid metabolism	GQ INCREASED	0.44
561	ENSDARP00000079035	neu3.3	neu3.3-201	Sialidase 3, tandem duplicate 4	Lipid metabolism	GQ INCREASED	0.44
562	ENSDARP00000032037	pdcd5	pdcd5-001	Programmed cell death 5	Apoptosis related	GQ INCREASED	0.44
563	ENSDARP00000116530	snx3	snx3-003	Sorting nexin-3/12	Protein degradation and synthesis inhibition	GQ INCREASED	0.44
564	ENSDARP00000075204	SEPT7-	SEPT7-201	Cytoskeleton proteins	Cell cycle, division, growth and fate	GQ INCREASED	0.44
565	ENSDARP00000069063	hm:zeh0351	hm:zeh0351	Cytoskeleton proteins	Cell cycle, division, growth and fate	GQ INCREASED	0.44
566	ENSDARP00000117207	CU469531.2	CU469531.2-201	Ras-related protein Rab-11B	Other	GQ INCREASED	0.44
567	ENSDARP00000124654	glyctk	glyctk-001	Glycerate kinase	Energy metabolism	GQ INCREASED	0.43
568	ENSDARP00000113159	ppp2r5d	ppp2r5d-001	Protein phosphatase 2, regulatory subunit B', delta	Protein Synthesis	GQ INCREASED	0.43
569	ENSDARP00000012850	asns	asns-201	Asparagine synthetase (glutamine-hydrolyzing)	Protein Synthesis	GQ INCREASED	0.43
570	ENSDARP00000107713	asns	asns-001	Asparagine synthetase (glutamine-hydrolyzing)	Protein Synthesis	GQ INCREASED	0.43
571	ENSDARP00000110193	asns	asns-202	Asparagine synthetase (glutamine-hydrolyzing)	Protein Synthesis	GQ INCREASED	0.43
572	ENSDARP00000042511	pitpnbl	pitpnbl-201	Phosphatidylinositol transfer protein, beta, like	Lipid metabolism	GQ INCREASED	0.43
573	ENSDARP00000095113	pitpnbl	pitpnbl-202	Phosphatidylinositol transfer protein, beta, like	Lipid metabolism	GQ INCREASED	0.43
574	ENSDARP00000113091	adsl	adsl-002	Adenylosuccinate lyase	Cell cycle, division, growth and fate	GQ INCREASED	0.43
575	ENSDARP00000074214	ndufs2	ndufs2-201	NADH dehydrogenase (ubiquinone) Fe-S protein 2	Energy metabolism	GQ INCREASED	0.43
576	ENSDARP00000026858	sept8b	sept8b-001	Septin 8a	Cell cycle, division, growth and fate	GQ INCREASED	0.43
577	ENSDARP00000012094	snx3	snx3-001	Sorting nexin-3/12		GQ INCREASED	0.43
578	ENSDARP00000124720	eif3eb	eif3eb-003	Eukaryotic translation initiation factor	Protein Synthesis	GQ INCREASED	0.43
579	ENSDARP00000114890	acat2	acat2-002	Acetyl-CoA acetyltransferase 2	Energy metabolism	GQ INCREASED	0.43
580	ENSDARP00000121223	sept8a	sept8a-002	Septin 8a	Cell cycle, division, growth and fate	GQ INCREASED	0.43
581	ENSDARP00000058796	hebp2	hebp2-201	Heme binding protein 2	Other	GQ INCREASED	0.43
582	ENSDARP00000062535	hebp2	hebp2-001	Heme binding protein 2	Other	GQ INCREASED	0.43
583	ENSDARP00000114030	scarb1	scarb1-002	Scavenger receptor class B, member 1	Endosome-Lysosome related	GQ INCREASED	0.43
584	ENSDARP00000123981	mcm7	mcm7-001	Inichromosome maintenance complex component 7	Cell cycle, division, growth and fate	GQ INCREASED	0.43
585	ENSDARP00000122752	pcca	pcca-003	Propionyl CoA carboxylase, alpha polypeptide	Energy metabolism	GQ INCREASED	0.43
586	ENSDARP00000070414	vtnb	vtnb-001	Vitronectin b	Immune system related	GQ INCREASED	0.43
587	ENSDARP00000106695	NHLRC3	NHLRC3-201	NHL repeat containing 3	Other	GQ INCREASED	0.43
588	ENSDARP00000042249	rap1b	rap1b-201	RAP1B, member of RAS oncogene family	Oncogenes related	GQ INCREASED	0.43
589	ENSDARP00000114387	rap1b	rap1b-001	RAP1B, member of RAS oncogene family	Oncogenes related	GQ INCREASED	0.43
590	ENSDARP00000035945	sccpdhb	sccpdhb-001	Saccharopine dehydrogenase b	Other	GQ INCREASED	0.43
591	ENSDARP00000052702	nucb2b	nucb2b-001	Nucleobindin 2	Other	GQ INCREASED	0.43
592	ENSDARP00000129420	ddi2	ddi2-002	DNA-damage inducible protein 2		GQ INCREASED	0.43
593	ENSDARP00000068144	pcxb	pcxb-201	Pyruvate carboxylase b	Energy metabolism	GQ INCREASED	0.43
594	ENSDARP00000017335	gorasp2	gorasp2-001	Golgi reassembly stacking protein 2, 55kDa	Protein Synthesis	GQ INCREASED	0.43
595	ENSDARP00000069178	si:ch1073-13h15.3	si:ch1073-13h15.3-201	Putative all-trans-retinol 13,14-reductase	REDOX/Detox related	GQ INCREASED	0.43
596	ENSDARP00000123687	shmt1	shmt1-003	Serine hydroxymethyltransferase 1 (soluble)	Protein Synthesis	GQ INCREASED	0.42
597	ENSDARP00000121832	shmt1	shmt1-002	Serine hydroxymethyltransferase 1 (soluble)	Protein Synthesis	GQ INCREASED	0.42
598	ENSDARP00000025374	uap1l1	uap1l1-001	UDP-N-acteylglucosamine pyrophosphorylase 1, like 1	Cell cycle, division, growth and fate	GQ INCREASED	0.42
599	ENSDARP00000054618	prps1b	prps1b-001	Phosphoribosyl pyrophosphate synthetase 1A	Protein Synthesis	GQ INCREASED	0.41
600	ENSDARP00000090402	eif3s6ip	eif3s6ip-201	Eukaryotic translation initiation factor 3, subunit 6 interacting protein	Protein Synthesis	GQ INCREASED	0.40
601	ENSDARP00000112498	eif3s6ip	eif3s6ip-202	Eukaryotic translation initiation factor 3, subunit 6 interacting protein	Protein Synthesis	GQ INCREASED	0.40
602	ENSDARP00000096295	ACTR3 (1 of 2)	ACTR3 (1 of 2)-201	ARP3 actin-related protein 3 homolog (yeast)	Cell cycle, division, growth and fate	GQ INCREASED	0.40
603	ENSDARP00000124779	cat	cat-001	Catalase	REDOX/Detox related	GQ INCREASED	0.40

604	ENSDARP00000026554	psmc3	psmc3-201	Proteasome (prosome, macropain) 26S subunit, ATPase, 3	Protein degradation and synthesis inhibition	GQ INCREASED	0.40
605	ENSDARP00000081816	ddx6	ddx6-201	DEAD (Asp-Glu-Ala-Asp) box helicase 6	Cell cycle, division, growth and fate	GQ INCREASED	0.40
606	ENSDARP00000120609	rps18	rps18-005	Small subunit ribosomal protein S18e	Protein Synthesis	GQ INCREASED	0.39
607	ENSDARP00000016831	cct6a	cct6a-201	Chaperonin containing TCP1, subunit 6A	Protein Synthesis	GQ INCREASED	0.39
608	ENSDARP00000118006	cct6a	cct6a-001	Chaperonin containing TCP1, subunit 6A	Protein Synthesis	GQ INCREASED	0.39
609	ENSDARP00000063876	prdx6	prdx6-001	Peroxiredoxin 6	REDOX/Detox related	GQ INCREASED	0.39
610	ENSDARP00000013402	cat	cat-201	Catalase	REDOX/Detox related	GQ INCREASED	0.38
611	ENSDARP00000107673	cat	cat-202	Catalase	REDOX/Detox related	GQ INCREASED	0.38
612	ENSDARP00000098561	cnp	cnp-202	2',3'-cyclic nucleotide 3' phosphodiesterase	Cell cycle, division, growth and fate	GQ INCREASED	0.38
613	ENSDARP00000117370	cnp	cnp-001	2',3'-cyclic nucleotide 3' phosphodiesterase	Cell cycle, division, growth and fate	GQ INCREASED	0.38
614	ENSDARP00000093249	dars	dars-201	Aspartyl-tRNA synthetase	Protein Synthesis	GQ INCREASED	0.38
615	ENSDARP00000090277	shmt2	shmt2-201	Serine hydroxymethyltransferase 2 (mitochondrial)	Protein Synthesis	GQ INCREASED	0.37
616	ENSDARP00000051290	hexa	hexa-001	Hexosaminidase A (alpha polypeptide)	Energy metabolism	GQ INCREASED	0.37
617	ENSDARP00000042258	clybl	clybl-001	Citrate lyase subunit beta / citryl-CoA lyase	Energy metabolism	GQ INCREASED	0.37
618	ENSDARP00000025466	cct4	cct4-201	Chaperonin containing Tcp1, subunit 4	Protein Synthesis	GQ INCREASED	0.37
619	ENSDARP00000102802	shmt2	shmt2-202	Serine hydroxymethyltransferase 2 (mitochondrial)	Protein Synthesis	GQ INCREASED	0.37
620	ENSDARP00000074247	vdac3	vdac3-201	Voltage-dependent anion channel 3	Other	GQ INCREASED	0.37
621	ENSDARP00000121437	vdac3	vdac3-001	Voltage-dependent anion channel 3	Other	GQ INCREASED	0.37
622	ENSDARP00000101564	CU914772.1	CU914772.1-201	Glycosidase	Energy metabolism	GQ INCREASED	0.37
623	ENSDARP00000102398	eif3s10	eif3s10-001	Eukaryotic translation initiation factor	Protein Synthesis	GQ INCREASED	0.37
624	ENSDARP00000052502	nudcd1	nudcd1-001	NudC domain containing 1	Other	GQ INCREASED	0.36
625	ENSDARP00000111483	kpna2	kpna2-202	Karyopherin alpha 2	Protein Synthesis	GQ INCREASED	0.36
626	ENSDARP00000055489	kpna2	kpna2-001	Karyopherin alpha 2	Protein Synthesis	GQ INCREASED	0.36
627	ENSDARP00000117923	eno3	eno3-002	Enolase 3	Energy metabolism	GQ INCREASED	0.36
628	ENSDARP00000014955	man2b1	man2b1-001	Mannosidase, alpha, class 2B, member 1	Energy metabolism	GQ INCREASED	0.35
629	ENSDARP00000006424	agla	agla-201	Amylo-1, 6-glucosidase, 4-alpha-glucanotransferase a	Energy metabolism	GQ INCREASED	0.35
630	ENSDARP00000129094	agla	agla-001	Amylo-1, 6-glucosidase, 4-alpha-glucanotransferase a	Energy metabolism	GQ INCREASED	0.35
631	ENSDARP00000006882	me3	me3-001	Mailc enzyme	Energy metabolism	GQ INCREASED	0.35
632	ENSDARP00000075198	uba3	uba3-001	Ubiquitin-like modifier activating enzyme 3	Protein degradation and synthesis inhibition	GQ INCREASED	0.35
633	ENSDARP00000123254	pcxb	pcxb-001	Pyruvate carboxylase b	Energy metabolism	GQ INCREASED	0.35
634	ENSDARP00000041577	stx12	stx12-201	Syntaxin 12/13	Other	GQ INCREASED	0.35
635	ENSDARP00000107227	stx12	stx12-001	Syntaxin 12/13	Other	GQ INCREASED	0.35
636	ENSDARP00000072874	rps8a	rps8a-001	Ribosomal protein S8a	Protein Synthesis	GQ INCREASED	0.35
637	ENSDARP00000123532	vdac3	vdac3-002	Voltage-dependent anion channel 3	Cell cycle, division, growth and fate	GQ INCREASED	0.35
638	ENSDARP00000003850	akr7a3	akr7a3-201	Aldo-keto reductase family 7, member A3 -aflatoxin B1 aldehyde reductase	REDOX/Detox related	GQ INCREASED	0.35
639	ENSDARP00000005719	rps18	rps18-001	Small subunit ribosomal protein S18e	Protein Synthesis	GQ INCREASED	0.35
640	ENSDARP00000008399	drg2	drg2-001	Developmentally regulated GTP binding protein 2	Cell cycle, division, growth and fate	GQ INCREASED	0.35
641	ENSDARP00000105639	drg2	drg2-002	Developmentally regulated GTP binding protein 2	Cell cycle, division, growth and fate	GQ INCREASED	0.35
642	ENSDARP00000112087	zgc:158614	zgc:158614-202	Aifm4-apoptosis-inducing factor, mitochondrion-associated, 4	Apoptosis related	GQ INCREASED	0.34
643	ENSDARP00000129014	zgc:158614	zgc:158614-001	Aifm4-apoptosis-inducing factor, mitochondrion-associated, 4	Apoptosis related	GQ INCREASED	0.34
644	ENSDARP00000110981	dars	dars-202	Aspartyl-tRNA synthetase	Protein Synthesis	GQ INCREASED	0.33
645	ENSDARP00000116150	uap1	uap1-002	UDP-N-acteylglucosamine pyrophosphorylase 1	Cell cycle, division, growth and fate	GQ INCREASED	0.33
646	ENSDARP00000005651	rpl8	rpl8-201	Ribosomal protein L8	Protein Synthesis	GQ INCREASED	0.33
647	ENSDARP00000115147	rpl8	rpl8-001	Ribosomal protein L8	Protein Synthesis	GQ INCREASED	0.33
648	ENSDARP00000006244	dnpep	dnpep-001	Aspartyl aminopeptidase	Protein degradation and synthesis inhibition	GQ INCREASED	0.33
649	ENSDARP00000110484	dnpep	dnpep-201	Aspartyl aminopeptidase	Protein degradation and synthesis inhibition	GQ INCREASED	0.33
650	ENSDARP00000027655	rnpep	rnpep-001	Cytochrome-c peroxidase	Energy metabolism	GQ INCREASED	0.33
651	ENSDARP00000098660	snrnp200	snrnp200-001	Small nuclear ribonucleoprotein 200kDa	Protein Synthesis	GQ INCREASED	0.32
652	ENSDARP00000070479	cpdb	cpdb-001	Carboxypeptidase D, b	Protein degradation and synthesis inhibition	GQ INCREASED	0.30
653	ENSDARP00000040031	rtn4ip1	rtn4ip1-001	Reticulon 4 interacting protein 1	Protein Synthesis	GQ INCREASED	0.29
654	ENSDARP00000124612	glyctk	glyctk-004	Glycerate kinase	Energy metabolism	GQ INCREASED	0.29
655	ENSDARP00000121663	clybl	clybl-002	Citrate lyase subunit beta / citryl-CoA lyase	Energy metabolism	GQ INCREASED	0.29
656	ENSDARP00000114273	tceb2	tceb2-001	Transcription elongation factor B, polypeptide 2	Protein degradation and synthesis inhibition	GQ INCREASED	0.29
657	ENSDARP00000128442	zgc:158614	zgc:158614-003	Aifm4-apoptosis-inducing factor, mitochondrion-associated, 4	Apoptosis related	GQ INCREASED	0.29
658	ENSDARP00000055359	tceb2	tceb2-002	Transcription elongation factor B, polypeptide 2	Protein degradation and synthesis inhibition	GQ INCREASED	0.29
659	ENSDARP00000084961	rhoae	rhoae-001	Ras homolog gene family, member A	Cell cycle, division, growth and fate	GQ INCREASED	0.29
660	ENSDARP00000123757	glyctk	glyctk-003	Glycerate kinase	Energy metabolism	GQ INCREASED	0.29
661	ENSDARP00000118814	rpl23a	rpl23a-004	Large subunit ribosomal protein L32e	Protein Synthesis	GQ INCREASED	0.29
662	ENSDARP00000104485	zgc:158614	zgc:158614-201	Aifm4-apoptosis-inducing factor, mitochondrion-associated, 4	Apoptosis related	GQ INCREASED	0.29
663	ENSDARP00000002234	oxct1a	oxct1a-001	3-oxoacid CoA transferase 1a	Energy metabolism	GQ INCREASED	0.29
664	ENSDARP00000089234	APOD (3 of 3)	APOD (3 of 3)-201	Apolipoprotein D	Lipid metabolism	GQ INCREASED	0.29
665	ENSDARP00000113343	APOD (3 of 3)	APOD (3 of 3)-001	Apolipoprotein D	Lipid metabolism	GQ INCREASED	0.29
666	ENSDARP00000117077	APOD (3 of 3)	APOD (3 of 3)-004	Apolipoprotein D	Lipid metabolism	GQ INCREASED	0.29
667	ENSDARP00000123122	APOD (3 of 3)	APOD (3 of 3)-003	Apolipoprotein D	Lipid metabolism	GQ INCREASED	0.29
668	ENSDARP00000123418	APOD (3 of 3)	APOD (3 of 3)-002	Apolipoprotein D	Lipid metabolism	GQ INCREASED	0.29
669	ENSDARP00000035324	selenbp1	selenbp1-201	Selenium binding protein 1	Protein Synthesis	GQ INCREASED	0.29
670	ENSDARP00000062897	ephx1	ephx1-001	Epoxide hydrolase 1, microsomal (xenobiotic)	REDOX/Detox related	GQ INCREASED	0.29
671	ENSDARP00000121216	rhoad	rhoad-002	Ras homolog gene family, member A	Cell cycle, division, growth and fate	GQ INCREASED	0.29

672	ENSDARP00000006624	rpl23a	rpl23a-001	Large subunit ribosomal protein L32e	Protein Synthesis	GQ INCREASED	0.29
673	ENSDARP00000022829	dph5	dph5-201	Diphthine synthas	Protein Synthesis	GQ INCREASED	0.29
674	ENSDARP00000108734	dph5	dph5-202	Diphthine synthas	Protein Synthesis	GQ INCREASED	0.29
675	ENSDARP00000111794	dph5	dph5-203	Diphthine synthas	Protein Synthesis	GQ INCREASED	0.29
676	ENSDARP00000082459	prmt5	prmt5-201	Protein arginine methyltransferase 5	Protein Synthesis	GQ INCREASED	0.29
677	ENSDARP00000116916	rps18	rps18-002	Small subunit ribosomal protein S18e	Protein Synthesis	GQ INCREASED	0.29
678	ENSDARP00000122082	rps18	rps18-003	Small subunit ribosomal protein S18e	Protein Synthesis	GQ INCREASED	0.29
679	ENSDARP00000052267	pitpnb	pitpnb-201	Phosphatidylinositol transfer protein, beta, like	Lipid metabolism	GQ INCREASED	0.29
680	ENSDARP00000124921	sept7b	Novel	Cytoskeleton proteins	Cell cycle, division, growth and fate	GQ INCREASED	0.29
681	ENSDARP00000068657	eif2a	eif2a-201	Eukaryotic translation initiation factor	Protein Synthesis	GQ INCREASED	0.29
682	ENSDARP00000125913	eif2a	eif2a-001	Eukaryotic translation initiation factor	Protein Synthesis	GQ INCREASED	0.29
683	ENSDARP00000115411	pdhb	pdhb-002	Pyruvate dehydrogenase (lipoamide) beta	Energy metabolism	GQ INCREASED	0.29
684	ENSDARP00000111558	pitpnb	pitpnb-202	Phosphatidylinositol transfer protein, beta, like	Lipid metabolism	GQ INCREASED	0.29
685	ENSDARP00000124765	glyctk	glyctk-002	Glycerate kinase	Energy metabolism	GQ INCREASED	0.29
686	ENSDARP00000109870	hdac10	hdac10-001	Histone deacetylase 10	Cell cycle, division, growth and fate	GQ INCREASED	0.29
687	ENSDARP00000067534	cap2	cap2-201	CAP, adenylate cyclase-associated protein, 2 (yeast)	Other	GQ INCREASED	0.29
688	ENSDARP00000106164	cltcb	cltcb-201	Clathrin, heavy polypeptide b	Protein degradation and synthesis inhibition	GQ INCREASED	0.28
689	ENSDARP00000105470	cltcb	cltcb-202	Clathrin, heavy polypeptide b	Protein degradation and synthesis inhibition	GQ INCREASED	0.28
690	ENSDARP00000119038	cltcb	cltcb-001	Clathrin, heavy polypeptide b	Protein degradation and synthesis inhibition	GQ INCREASED	0.28
691	ENSDARP00000077445	acad9	acad9-001	Acyl-CoA dehydrogenase family, member 9	Energy metabolism	GQ INCREASED	0.28
692	ENSDARP00000103810	prep	prep-001	Prolyl endopeptidase	Protein degradation and synthesis inhibition	GQ INCREASED	0.26
693	ENSDARP00000048675	prkar2aa	prkar2aa-001	Protein kinase, cAMP-dependent, regulatory, type II, alpha A	Apoptosis related	GQ INCREASED	0.26
694	ENSDARP00000046358	myh10	myh10-201	Myosin, heavy chain 10, non-muscle	Cell cycle, division, growth and fate	GQ INCREASED	0.26
695	ENSDARP00000125918	myh10	myh10-001	Myosin, heavy chain 10, non-muscle	Cell cycle, division, growth and fate	GQ INCREASED	0.26
696	ENSDARP00000007817	farsb	farsb-001	Phenylalanyl-tRNA synthetase, beta subunit	Protein Synthesis	GQ INCREASED	0.26
697	ENSDARP00000125771	myh10	myh10-002	Myosin, heavy chain 10, non-muscle	Cell cycle, division, growth and fate	GQ INCREASED	0.26
698	ENSDARP00000116604	selenbp1	selenbp1-001	Selenium binding protein 1	Protein Synthesis	GQ INCREASED	0.26
699	ENSDARP00000039986	pgm3	pgm3-001	Phosphoacetylglucosamine mutase	Energy metabolism	GQ INCREASED	0.26
700	ENSDARP00000023819	mccc2	mccc2-001	Methylcrotonoyl-CoA carboxylase 2 (beta)	Protein degradation and synthesis inhibition	GQ INCREASED	0.25
701	ENSDARP00000015404	ehd1a	ehd1a-201	EH domain-containing protein 1	Lipid metabolism	GQ INCREASED	0.25
702	ENSDARP00000057947	anxa11a	anxa11a-002	Annexin A11a	Immune system related	GQ INCREASED	0.24
703	ENSDARP00000092551	anxa11a	anxa11a-001	Annexin A11a	Immune system related	GQ INCREASED	0.24
704	ENSDARP00000052941	serpinh2	serpinh2	Serpin peptidase inhibitor, clade H (heat shock protein 47), member 1, (collagen binding protein 1)	Other	GQ INCREASED	0.24
705	ENSDARP00000105167	serpinh2	serpinh2	Serpin peptidase inhibitor, clade H (heat shock protein 47), member 1, (collagen binding protein 1)	Other	GQ INCREASED	0.24
706	ENSDARP00000125495	GBE1 (2 of 2)	GBE1 (2 of 2)-001	Glucan (1,4-alpha-), branching enzyme 1	Energy metabolism	GQ INCREASED	0.24
707	ENSDARP00000086805	myh9b	myh9b-201	Myosin, heavy polypeptide 9b, non-muscle	Cell cycle, division, growth and fate	GQ INCREASED	0.24
708	ENSDARP00000114437	myh9b	myh9b-001	Myosin, heavy polypeptide 9b, non-muscle	Cell cycle, division, growth and fate	GQ INCREASED	0.23
709	ENSDARP00000068053	tes	tes-201	Testis derived transcript (3 LIM domains)	Cell cycle, division, growth and fate	GQ INCREASED	0.23
710	ENSDARP00000111043	supt16h	supt16h-203	Suppressor of Ty 16 homolog (S. cerevisiae) (FACT complex subunit SPT16)	Protein Synthesis	GQ INCREASED	0.22
711	ENSDARP00000039188	lmf2b	lmf2b-201	Lipase maturation factor 2b	Protein Synthesis	GQ INCREASED	0.22
712	ENSDARP00000100277	supt16h	supt16h-201	Suppressor of Ty 16 homolog (S. cerevisiae) (FACT complex subunit SPT16)	Protein Synthesis	GQ INCREASED	0.22
713	ENSDARP00000109077	supt16h	supt16h-202	Suppressor of Ty 16 homolog (S. cerevisiae) (FACT complex subunit SPT16)	Protein Synthesis	GQ INCREASED	0.22
714	ENSDARP00000014578	gpib	gpib-201	Glucose phosphate isomerase b	Energy metabolism	GQ INCREASED	0.22
715	ENSDARP00000089816	atp6v1h	atp6v1h-201	V-type H+-transporting ATPase subunit H	Energy metabolism	GQ INCREASED	0.22
716	ENSDARP00000113453	atp6v1h	atp6v1h-002	V-type H+-transporting ATPase subunit H	Energy metabolism	GQ INCREASED	0.22
717	ENSDARP00000068054	tes	tes-001	Testis derived transcript (3 LIM domains)	Cell cycle, division, growth and fate	GQ INCREASED	0.22
718	ENSDARP00000113128	arcn1b	arcn1b-002	Archain 1b	Other	GQ INCREASED	0.22
719	ENSDARP00000073441	cpvl	cpvl-001	Vitellogenic carboxypeptidase-like protein	Protein degradation and synthesis inhibition	GQ INCREASED	0.22
720	ENSDARP00000094422	si:ch211-160d14.1	si:ch211-160d14.1-001	Fascin	Cell cycle, division, growth and fate	GQ INCREASED	0.22
721	ENSDARP00000024886	atp6v1h	atp6v1h-001	V-type H+-transporting ATPase subunit H	Energy metabolism	GQ INCREASED	0.22
722	ENSDARP00000074155	nle1	nle1-002	Notchless homolog 1 (Drosophila)	Protein Synthesis	GQ INCREASED	0.22
723	ENSDARP00000122687	nle1	nle1-001	Notchless homolog 1 (Drosophila)	Protein Synthesis	GQ INCREASED	0.22
724	ENSDARP00000083458	lap3	lap3-001	Leucine aminopeptidase 3	Protein degradation and synthesis inhibition	GQ INCREASED	0.22
725	ENSDARP00000058889	ephx2	ephx2-001	Epoxide hydrolase 2, cytoplasmic	Lipid metabolism	GQ INCREASED	0.20
726	ENSDARP00000117022	cratb	cratb-001	Carnitine O-acetyltransferase b	Energy metabolism	GQ INCREASED	0.20
727	ENSDARP00000129034	eml2	eml2-001	Echinoderm microtubule associated protein like 2	Cell cycle, division, growth and fate	GQ INCREASED	0.20
728	ENSDARP00000008223	eml2	eml2-201	Echinoderm microtubule associated protein like 2	Cell cycle, division, growth and fate	GQ INCREASED	0.20
729	ENSDARP00000101418	GBE1 (2 of 2)	GBE1 (2 of 2)-201	Glucan (1,4-alpha-), branching enzyme 1	Energy metabolism	GQ INCREASED	0.19
730	ENSDARP00000083382	fbxo10	fbxo10-001	F-box protein 10	Protein degradation and synthesis inhibition	GQ INCREASED	0.18
731	ENSDARP00000094289	cpne1	cpne1-202	Copine I	Other	GQ INCREASED	0.18
732	ENSDARP00000121695	si:ch211-93f2.1	Novel	Carboxylic ester hydrolase	REDOX/Detox related	GQ INCREASED	0.18
733	ENSDARP00000017582	zc3h15	zc3h15-001	Zinc finger CCCH-type containing 15	Protein Synthesis	GQ INCREASED	0.18
734	ENSDARP00000082086	cpne1	cpne1-201	Copine I	Other	GQ INCREASED	0.18
735	ENSDARP00000007446	cox4i1	cox4i1-001	Cytochrome c oxidase subunit IV isoform 1	Energy metabolism	GQ INCREASED	0.17
736	ENSDARP00000035261	pfdn2	pfdn2-001	Prefoldin subunit 2	Protein Synthesis	GQ INCREASED	0.17
737	ENSDARP00000068644	BX322787.1	BX322787.1-201	ATP-binding cassette, sub-family F, member 2-like	Protein Synthesis	GQ INCREASED	0.17
738	ENSDARP00000119994	eno1a	eno1a-002	Enolase 1 a	Energy metabolism	GQ INCREASED	0.17
739	ENSDARP00000053867	aldh9a1b	aldh9a1b-001	Aldehyde dehydrogenase 9 family, member A1b	Lipid metabolism	GQ INCREASED	0.16

740	ENSDARP00000034052	pdcd4a	pdcd4a-201	Programmed cell death protein 4	Apoptosis related	GQ INCREASED	0.16
741	ENSDARP00000011311	arcn1a	arcn1a-001	Archain 1a	Other	GQ INCREASED	0.15
742	ENSDARP00000101190	RAP1GDS1	RAP1GDS1-201	RAP1, GTP-GDP dissociation stimulator 1	Other	GQ INCREASED	0.15
743	ENSDARP00000113140	larsb	larsb-001	Leucyl-tRNA synthetase b	Protein Synthesis	GQ INCREASED	0.15
744	ENSDARP00000004838	rcc2	rcc2-001	Regulator of chromosome condensation 2	Cell cycle, division, growth and fate	GQ INCREASED	0.15
745	ENSDARP00000123019	zp3c	zp3c-001	Zona pellucida protein	Zona Pellucida proteins	GQ INCREASED	0.15
746	ENSDARP00000003359	larsb	larsb-201	Leucyl-tRNA synthetase b	Protein Synthesis	GQ INCREASED	0.14
747	ENSDARP00000011011	eif3d	eif3d-201	Eukaryotic translation initiation factor	Protein Synthesis	GQ INCREASED	0.12
748	ENSDARP00000112626	oxct1a	oxct1a-003	3-oxoacid CoA transferase 1a	Energy metabolism	GQ INCREASED	0.12
749	ENSDARP00000116681	eif3d	eif3d-001	Eukaryotic translation initiation facto	Protein Synthesis	GQ INCREASED	0.12
750	ENSDARP00000056639	abcf2a	abcf2a-001	ATP-binding cassette, sub-family F (GCN20), member 2a	Protein Synthesis	GQ INCREASED	0.12
751	ENSDARP00000116165	cltcb	cltcb-003	Clathrin, heavy polypeptide b	Protein degradation and synthesis inhibition	GQ INCREASED	0.12
752	ENSDARP00000003738	eno1a	eno1a-001	Enolase 1 a	Energy metabolism	GQ INCREASED	0.12
753	ENSDARP00000047901	arcn1b	arcn1b-001	Archain 1b	Other	GQ INCREASED	0.12
754	ENSDARP00000020181	ehd1b	ehd1b-001	EH-domain containing 3	Protein degradation and synthesis inhibition	GQ INCREASED	0.11
755	ENSDARP00000051798	hdac1	hdac1-001	Histone deacetylase 1	Cell cycle, division, growth and fate	GQ INCREASED	0.11
756	ENSDARP00000095265	si:ch211-93f2.1	Novel	Carboxylic ester hydrolase-esterase lipase sueprfamily-drug metabolism	REDOX/Detox related	GQ INCREASED	0.10
757	ENSDARP00000021016	zgc:158620	Novel	Tubulin, beta 2A class IIa	Cell cycle, division, growth and fate	GQ UNIQUE	-
758	ENSDARP00000016957	rpl36	rpl36-001	Ribosomal protein L36	Protein Synthesis	GQ UNIQUE	-
759	ENSDARP00000124304	rpl36	rpl36-002	Ribosomal protein L36	Protein Synthesis	GQ UNIQUE	-
760	ENSDARP00000012767		aldh2.1-001	Aldehyde dehydrogenase 2 family (mitochondrial), tandem duplicate 2	Energy metabolism	GQ UNIQUE	-
761	ENSDARP00000118282	oxct1a	oxct1a-004	3-oxoacid CoA transferase 1a	Lipid metabolism	GQ UNIQUE	-
762	ENSDARP00000109625	tubb2	tubb2-001 (novel)	Beta tubulin	Cell cycle, division, growth and fate	GQ UNIQUE	-
763	ENSDARP00000108963	CABZ01032488.1	CABZ01032488.1-201	Aldehyde dehydrogenase super family	Energy metabolism	GQ UNIQUE	-
764	ENSDARP00000110851	FP102463.1	FP102463.1-201	Beta tubulin	Cell cycle, division, growth and fate	GQ UNIQUE	-
765	ENSDARP00000126192	eif3d	eif3d-004	Eukaryotic translation initiation factor 3, subunit D	Protein Synthesis	GQ UNIQUE	-
766	ENSDARP00000035541	ube2l3a	ube2l3a-201	Ubiquitin-conjugating enzyme E2L 3a	Cell cycle, division, growth and fate	GQ UNIQUE	-
767	ENSDARP00000054355	pam16	pam16-001	Presequence translocase-associated motor 16 homolog (S. cerevisiae)	Energy metabolism	GQ UNIQUE	-
768	ENSDARP00000072006	tuba1c	tuba1c-001	Tubulin alpha 1c	Cell cycle, division, growth and fate	GQ UNIQUE	-
769	ENSDARP00000016749	me2	me2-001	Malic enzyme 2, NAD(+)-dependent, mitochondrial	Energy metabolism	GQ UNIQUE	-
770	ENSDARP00000109682	me2	me2-201	Malic enzyme 2, NAD(+)-dependent, mitochondrial	Energy metabolism	GQ UNIQUE	-
771	ENSDARP00000127763	HIST1H2BA (9 of 9)	HIST1H2BA (9 of 9)-001	Histone	Cell cycle, division, growth and fate	GQ UNIQUE	-
772	ENSDARP00000115350	aldh9a1b	aldh9a1b-002	Aldehyde dehydrogenase 9 family, member A1b	Energy metabolism	GQ UNIQUE	-
773	ENSDARP00000088020	aldh2.2	aldh2.2-202	Aldehyde dehydrogenase 2 family (mitochondrial), tandem duplicate 2	Energy metabolism	GQ UNIQUE	-
774	ENSDARP00000088019	aldh2.2	aldh2.2-201	Aldehyde dehydrogenase 2 family (mitochondrial), tandem duplicate 2	Energy metabolism	GQ UNIQUE	-
775	ENSDARP00000105094	tuba1b	tuba1b-001	Tubulin alpha 1b	Cell cycle, division, growth and fate	GQ UNIQUE	-
776	ENSDARP00000083766	dak	dak-001	Dihydroxyacetone kinase 2 homolog (S. cerevisiae)	Energy metabolism	GQ UNIQUE	-
777	ENSDARP00000105448	pkma	pkma-202	Pyruvate kinase, muscle, a	Energy metabolism	GQ UNIQUE	-
778	ENSDARP00000105213	CABZ01112387.1	CABZ01112387.1-201	Myosin 9 like-myosin N super family	Cell cycle, division, growth and fate	GQ UNIQUE	-
779	ENSDARP00000009846	arf1	arf1-201	ADP-ribosylation factor 1	Cell cycle, division, growth and fate	GQ UNIQUE	-
780	ENSDARP00000102498	arf1	arf1-202	ADP-ribosylation factor 1	Cell cycle, division, growth and fate	GQ UNIQUE	-
781	ENSDARP00000128933	ppp5c	ppp5c-001	Protein phosphatase 5, catalytic subunit	Cell cycle, division, growth and fate	GQ UNIQUE	-
782	ENSDARP00000110687	LOC101884557	Novel	Myosin 9 like-myosin N super family	Cell cycle, division, growth and fate	GQ UNIQUE	-
783	ENSDARP00000124733	me2	me2-002	Malic enzyme 2, NAD(+)-dependent, mitochondrial	Energy metabolism	GQ UNIQUE	-
784	ENSDARP00000114018	prmt3	prmt3-002	Protein arginine methyltransferase 3	Protein Synthesis	GQ UNIQUE	-
785	ENSDARP00000003829	prmt3	prmt3-001	Protein arginine methyltransferase 3	Protein Synthesis	GQ UNIQUE	-
786	ENSDARP00000122171	rpl8	rpl8-002	Ribosomal protein L8	Protein Synthesis	GQ UNIQUE	-
787	ENSDARP00000104932	sh3d21	Novel	Uncharacterized protein--SH3 domain-containing protein 21	Cell cycle, division, growth and fate	GQ UNIQUE	-
788	ENSDARP00000058636	sh3d21	Novel	Uncharacterized protein--SH3 domain-containing protein 21	Cell cycle, division, growth and fate	GQ UNIQUE	-
789	ENSDARP00000062517	zgc:162356	zgc:162356-001	Glutathione S-transferase-glutathione metabolism-xenobiotics biodegredation and metabolism	Cell cycle, division, growth and fate	GQ UNIQUE	-
790	ENSDARP00000104897	dbnlb	dbnlb-202	Drebrin-like b	Endosome-Lysosome related	GQ UNIQUE	-
791	ENSDARP00000112697	sh3d21	Novel	Uncharacterized protein--SH3 domain-containing protein 21	Cell cycle, division, growth and fate	GQ UNIQUE	-
792	ENSDARP00000122208	ERAP1 (2 of 2)	ERAP1 (2 of 2)-201	Endoplasmic reticulum aminopeptidase 1	Immune system related	GQ UNIQUE	-
793	ENSDARP00000095260	cox4i2	cox4i2-001	Cytochrome c oxidase subunit IV isoform 2	Energy metabolism	GQ UNIQUE	-
794	ENSDARP00000066122	KPNA6	KPNA6-201	Karyopherin alpha 6 (importin alpha 7	Protein Synthesis	GQ UNIQUE	-
795	ENSDARP00000117154	dnpep	dnpep-003	Aspartyl aminopeptidase	Protein degradation and synthesis inhibition	GQ UNIQUE	-
796	ENSDARP00000113894	hars	hars-002	Histidyl-tRNA synthetase	Protein Synthesis	GQ UNIQUE	-
797	ENSDARP00000051468	si:ch211-186e20.7	si:ch211-186e20.7-201	Serpin super family-alpha antitrypsin domain	Other	GQ UNIQUE	-
798	ENSDARP00000123705	si:ch211-186e20.7	si:ch211-186e20.7-001	Serpin super family-alpha antitrypsin domain	Other	GQ UNIQUE	-
799	ENSDARP00000114386	eno2	eno2-003	Enolase 2	Energy metabolism	GQ UNIQUE	-
800	ENSDARP00000117079	sh3d21	Novel	Uncharacterized protein--SH3 domain-containing protein 21	Cell cycle, division, growth and fate	GQ UNIQUE	-
801	ENSDARP00000124373	atp6v1h	atp6v1h-004	ATPase, H+ transporting, lysosomal, V1 subunit H	Endosome-Lysosome related	GQ UNIQUE	-
802	ENSDARP00000027911	dbnlb	dbnlb-201	Drebrin-like b	Endosome-Lysosome related	GQ UNIQUE	-
803	ENSDARP00000014921	zgc:92137	zgc:92137-201	alpha amylase	Energy metabolism	GQ UNIQUE	-
804	ENSDARP00000038975	dhdhl	dhdhl-001	Dihydrodiol dehydrogenase (dimeric), like	Energy metabolism	GQ UNIQUE	-
805	ENSDARP00000008466	hpx	hpx-201	Hemopexin	Other	GQ UNIQUE	-
806	ENSDARP00000111337	hpx	hpx-001	Hemopexin	Other	GQ UNIQUE	-
807	ENSDARP00000052498	gla	gla-201	Galactosidase, alpha	Lipid metabolism	GQ UNIQUE	-

808	ENSDARP00000075482	sb:cb252	sb:cb252-201	ES1 protein homolog, mitochondrial	Other	GQ UNIQUE	-
809	ENSDARP00000106738	larsa	larsa-201	Leucyl-tRNA synthetase a	Protein Synthesis	GQ UNIQUE	-
810	ENSDARP00000097448	trmt112	trmt112-001	TRNA methyltransferase 11-2 homolog (S. cerevisiae)	Protein Synthesis	GQ UNIQUE	-
811	ENSDARP00000114835	gla	gla-001	Galactosidase, alpha	Lipid metabolism	GQ UNIQUE	-
812	ENSDARP00000062263	sdhb	sdhb-201	Succinate dehydrogenase complex, subunit B, iron sulfur (Ip)	Energy metabolism	GQ UNIQUE	-
813	ENSDARP00000019062	vars	vars-201	Valyl-tRNA synthetase	Protein Synthesis	GQ UNIQUE	-
814	ENSDARP00000120746	ces3	ces3-001	Carboxylesterase 3	Lipid metabolism	GQ UNIQUE	-
815	ENSDARP00000107172	ces3	ces3-203	Carboxylesterase 3	Lipid metabolism	GQ UNIQUE	-
816	ENSDARP00000021453	amy2a	amy2a-001	Amylase, alpha 2A; pancreatic	Energy metabolism	GQ UNIQUE	-
817	ENSDARP00000091190	lancl2	lancl2-201	LanC lantibiotic synthetase component C-like 2 (bacterial)	Immune system related	GQ UNIQUE	-
818	ENSDARP00000114912	lancl2	lancl2-001	LanC lantibiotic synthetase component C-like 2 (bacterial)	Immune system related	GQ UNIQUE	-
819	ENSDARP00000121084	dnpep	dnpep-002	Aspartyl aminopeptidase	Protein degradation and synthesis inhibition	GQ UNIQUE	-
820	ENSDARP00000046625	PRKAR2B	PRKAR2B-201	Protein kinase, cAMP-dependent, regulatory, type II, beta	Energy metabolism	GQ UNIQUE	-
821	ENSDARP00000023098	dlat	dlat-001	Dihydrolipoamide S-acetyltransferase (E2 component of pyruvate dehydrogenase complex)	Energy metabolism	GQ UNIQUE	-
822	ENSDARP00000091998	ddx39aa	ddx39aa-201	DEAD (Asp-Glu-Ala-Asp) box polypeptide 39Aa	Cell cycle, division, growth and fate	GQ UNIQUE	-
823	ENSDARP00000115525	oxsr1b	oxsr1b-001	Oxidative-stress responsive 1b	Cell cycle, division, growth and fate	GQ UNIQUE	-
824	ENSDARP00000126065	sesn2	sesn2-003	Sestrin 2	Cell cycle, division, growth and fate	GQ UNIQUE	-
825	ENSDARP00000119784	zgc:165539	zgc:165539-003	Mucin 5Ac	Immune system related	GQ UNIQUE	-
826	ENSDARP00000117895	ITPA (2 of 2)	ITPA (2 of 2)-201	Inosine triphosphatase (nucleoside triphosphate pyrophosphatase)	Cell cycle, division, growth and fate	GQ UNIQUE	-
827	ENSDARP00000123429	g3bp1	g3bp1-002	GTPase activating protein (SH3 domain) binding protein 1	Cell cycle, division, growth and fate	GQ UNIQUE	-
828	ENSDARP00000016959	carm1	carm1-001	Coactivator-associated arginine methyltransferase 1	Protein Synthesis	GQ UNIQUE	-
829	ENSDARP00000074674	itpa	itpa-001	Inosine triphosphatase (nucleoside triphosphate pyrophosphatase)	Cell cycle, division, growth and fate	GQ UNIQUE	-
830	ENSDARP00000105903	anxa6	anxa6-203	Annexin A6	Endosome-Lysosome related	GQ UNIQUE	-
831	ENSDARP00000006047	ugp2a	ugp2a-001	UDP-glucose pyrophosphorylase 2a	Energy metabolism	GQ UNIQUE	-
832	ENSDARP00000076180	rab25a	rab25a-201	RAB25, member RAS oncogene family a	Oncogenes related	GQ UNIQUE	-
833	ENSDARP00000008513	ddx39aa	ddx39aa-001	DEAD (Asp-Glu-Ala-Asp) box polypeptide 39Aa	Cell cycle, division, growth and fate	GQ UNIQUE	-
834	ENSDARP00000093160	sesn2	sesn2-001	Sestrin 2	Cell cycle, division, growth and fate	GQ UNIQUE	-
835	ENSDARP00000087672	papl	papl-201	Iron/zinc purple acid phosphatase-like protein	Protein degradation and synthesis inhibition	GQ UNIQUE	-
836	ENSDARP00000114865	eef1da	eef1da-005	Elongation factor-1, delta, a	Protein Synthesis	GQ UNIQUE	-
837	ENSDARP00000122064	pold2	pold2-001	Polymerase (DNA directed), delta 2, regulatory subunit	Cell cycle, division, growth and fate	GQ UNIQUE	-
838	ENSDARP00000125677	sesn2	sesn2-002	Sestrin 2	Cell cycle, division, growth and fate	GQ UNIQUE	-
839	ENSDARP00000041675	tph1a	tph1a-201	Tryptophan hydroxylase 1 (tryptophan 5-monooxygenase) a	Protein Synthesis	GQ UNIQUE	-
840	ENSDARP00000112644	sh3d21	Novel	SH3 domain-containing protein 21	Cell cycle, division, growth and fate	GQ UNIQUE	-
841	ENSDARP00000067158	tph1a	tph1a-202	Tryptophan hydroxylase 1 (tryptophan 5-monooxygenase) a	Protein Synthesis	GQ UNIQUE	-
842	ENSDARP00000113340	pold2	pold2-002	Polymerase (DNA directed), delta 2, regulatory subunit	Cell cycle, division, growth and fate	GQ UNIQUE	-
843	ENSDARP00000125959	carm1	carm1-003	Coactivator-associated arginine methyltransferase 1	Protein Synthesis	GQ UNIQUE	-
844	ENSDARP00000052682	c3c	c3c-001	Complement component c3c	Immune system related	GQ UNIQUE	-
845	ENSDARP00000083043	rangap1a	rangap1a-001	RAN GTPase activating protein 1a	Cell cycle, division, growth and fate	GQ UNIQUE	-
846	ENSDARP00000112996	phex	phex-002	Phosphate regulating gene with homologues to endopeptidases on the X chromosome	Protein degradation and synthesis inhibition	GQ UNIQUE	-
847	ENSDARP00000009876	memo1	memo1-001	Mediator of cell motility 1	Cell cycle, division, growth and fate	GQ UNIQUE	-
848	ENSDARP00000025917	ppp6c	ppp6c-001	Protein phosphatase 6, catalytic subunit	Cell cycle, division, growth and fate	GQ UNIQUE	-
849	ENSDARP00000023671	flj13639	flj13639-201	Uncharacterized protein--short chain dehydrogenase/reductase	Other	GQ UNIQUE	-
850	ENSDARP00000113500	ndufaf5	ndufaf5-001	NADH dehydrogenase (ubiquinone) complex I, assembly factor 5	Energy metabolism	GQ UNIQUE	-
851	ENSDARP00000104359	si:ch211-165b10.3	si:ch211-165b10.3-201	Uncharacterized protein	Other	GQ UNIQUE	-
852	ENSDARP00000098664	ME1 (2 of 2)	ME1 (2 of 2)-201	Malic enzyme 1, NADP(+)-dependent, cytosolic	Energy metabolism	GQ UNIQUE	-
853	ENSDARP00000124689	nmt1a	nmt1a-002	N-myristoyltransferase 1a	Other	GQ UNIQUE	-
854	ENSDARP00000128976	si:ch211-165b10.3	si:ch211-165b10.3-001	Uncharacterized protein	Other	GQ UNIQUE	-
855	ENSDARP00000076538	PPP2R5C (2 of 2)	PPP2R5C (2 of 2)-201	Protein phosphatase 2, regulatory subunit B,, gamma	Cell cycle, division, growth and fate	GQ UNIQUE	-
856	ENSDARP00000026627	anxa6	anxa6-201	Annexin A6	Endosome-Lysosome related	GQ UNIQUE	-
857	ENSDARP00000081541	EIF4G1 (1 of 3)	EIF4G1 (1 of 3)-201	Eukaryotic translation initiation factor 4 gamma, 1	Protein Synthesis	GQ UNIQUE	-
858	ENSDARP00000081554	EIF4G1 (1 of 3)	EIF4G1 (1 of 3)-002	Eukaryotic translation initiation factor 4 gamma, 1	Protein Synthesis	GQ UNIQUE	-
859	ENSDARP00000098745	nomo	nomo-201	Nodal modulator	Cell cycle, division, growth and fate	GQ UNIQUE	-
860	ENSDARP00000128647	nomo	nomo-001	Nodal modulator	Cell cycle, division, growth and fate	GQ UNIQUE	-
861	ENSDARP00000010925	ddx39ab	ddx39ab-001	DEAD (Asp-Glu-Ala-Asp) box polypeptide 39Ab	Cell cycle, division, growth and fate	GQ UNIQUE	-
862	ENSDARP00000052354	ddx39b	ddx39b-001	DEAD (Asp-Glu-Ala-Asp) box polypeptide 39Ab	Cell cycle, division, growth and fate	GQ UNIQUE	-
863	ENSDARP00000069703	me1	me1-201	Malic enzyme 1, NADP(+)-dependent, cytosolic	Energy metabolism	GQ UNIQUE	-
864	ENSDARP00000012808	pah	pah-001	Phenylalanine hydroxylase	Protein degradation and synthesis inhibition	GQ UNIQUE	-
865	ENSDARP00000042296	kdelc1	kdelc1-001	KDEL (Lys-Asp-Glu-Leu) containing 1	Protein Synthesis	GQ UNIQUE	-
866	ENSDARP00000047072	oxsr1a	oxsr1a-001	Oxidative-stress responsive 1a	Cell cycle, division, growth and fate	GQ UNIQUE	-
867	ENSDARP00000055010	PDILT	PDILT-001	Protein disulfide isomerase-like, testis expressed	Protein Synthesis	GQ UNIQUE	-
868	ENSDARP00000066974	impdh1b	impdh1b-002	Inosine 5,-phosphate dehydrogenase 1b	Cell cycle, division, growth and fate	GQ UNIQUE	-
869	ENSDARP00000074184	oxct1b	oxct1b-001	3-oxoacid CoA transferase 1b	Lipid metabolism	GQ UNIQUE	-
870	ENSDARP00000066972	impdh1b	impdh1b-001	Inosine 5,-phosphate dehydrogenase 1b	Cell cycle, division, growth and fate	GQ UNIQUE	-
871	ENSDARP00000092803	kpna5	kpna5-001	Karyopherin alpha 5 (importin alpha 6)	Protein Synthesis	GQ UNIQUE	-
872	ENSDARP00000019185	impdh1a	impdh1a-001	Inosine 5,-phosphate dehydrogenase 1a	Cell cycle, division, growth and fate	GQ UNIQUE	-
873	ENSDARP00000125968	usp7	usp7-001	Ubiquitin specific peptidase 7 (herpes virus-associated)	Cell cycle, division, growth and fate	GQ UNIQUE	-
874	ENSDARP00000002502	usp7	usp7-201	Ubiquitin specific peptidase 7 (herpes virus-associated)	Cell cycle, division, growth and fate	GQ UNIQUE	-
875	ENSDARP00000096118	anxa6	anxa6-202	Annexin A6	Endosome-Lysosome related	GQ UNIQUE	-

876	ENSDARP00000124518	anxa6	anxa6-001	Annexin A6	Endosome-Lysosome related	GQ UNIQUE	-
877	ENSDARP00000014963	ppp2r5cb	ppp2r5cb-201	Protein phosphatase 2, regulatory subunit B,, gamma b	Cell cycle, division, growth and fate	GQ UNIQUE	-
878	ENSDARP00000101961	ppp2r5cb	ppp2r5cb-202	Protein phosphatase 2, regulatory subunit B,, gamma b	Cell cycle, division, growth and fate	GQ UNIQUE	-
879	ENSDARP00000103928	ppp2r5cb	ppp2r5cb-001	Protein phosphatase 2, regulatory subunit B,, gamma b	Cell cycle, division, growth and fate	GQ UNIQUE	-
880	ENSDARP00000012056	ctps1b	ctps1b-001	CTP synthase 1b	Cell cycle, division, growth and fate	GQ UNIQUE	-
881	ENSDARP00000028051	lmnl3	lmnl3-001	Lamin L3	Cell cycle, division, growth and fate	GQ UNIQUE	-
882	ENSDARP00000070709	ctps1b	ctps1b-201	CTP synthase 1b	Cell cycle, division, growth and fate	GQ UNIQUE	-
883	ENSDARP00000092744	me1	me1-002	Malic enzyme 1, NADP(+)-dependent, cytosolic	Energy metabolism	GQ UNIQUE	-
884	ENSDARP00000113885	me1	me1-001	Malic enzyme 1, NADP(+)-dependent, cytosolic	Energy metabolism	GQ UNIQUE	-
885	ENSDARP00000120790	kpna1	kpna1-001	Karyopherin alpha 1 (importin alpha 5)	Protein Synthesis	GQ UNIQUE	-
886	ENSDARP00000091935	impdh1a	impdh1a-201	Inosine 5,-phosphate dehydrogenase 1a	Cell cycle, division, growth and fate	GQ UNIQUE	-
887	ENSDARP00000059197	PARP14	PARP14-201	Poly (ADP-ribose) polymerase family, member 14	Apoptosis related	GQ UNIQUE	-
888	ENSDARP00000016922	aglb	aglb-001	Amylo-1, 6-glucosidase, 4-alpha-glucanotransferase b	Energy metabolism	GQ UNIQUE	-
889	ENSDARP00000098492	zfat	zfat-201	Zinc finger and AT hook domain containing	Protein Synthesis	GQ UNIQUE	-
890	ENSDARP00000127519	zfat	zfat-001	Zinc finger and AT hook domain containing	Protein Synthesis	GQ UNIQUE	-
891	ENSDARP00000025533	tln1	tln1-201	Talin 1	Cell cycle, division, growth and fate	GQ UNIQUE	-
892	ENSDARP00000072597	tln1	tln1-001	Talin 1	Cell cycle, division, growth and fate	GQ UNIQUE	-
