# Supplementary material for: Scrambled eggs: Proteomic portraits and novel biomarkers of egg quality in zebrafish (Danio rerio)
Source: PLoS One. 2017 Nov 16;12(11):e0188084. doi: 10.1371/journal.pone.0188084 (PMC5690628; doi:10.1371/journal.pone.0188084)
Supplement: S2 Table — (PDF) [file pone.0188084.s002.pdf]

S2 Table. Proteins differentially regulated in the Multiple Samples Experiment. List of the 274 proteins from the Multiple Samples Experiment that were considered to be differentially regulated between egg quality groups and whose distribution among various functional categories is illustrated in **Fig 2b**. These include proteins detected only in poor quality eggs (PQ UNIQUE, n=93), proteins in poor quality eggs with N-SC increased ≥2-fold relative to values for good quality eggs (PQ INCREASED, n=58), proteins detected only in good quality eggs (GQ UNIQUE, n=90), and proteins in good quality eggs with N-SC increased ≥2-fold relative to values for poor quality eggs (GQ INCREASED, n=32). For each protein, the Ensembl Protein ID and associated gene, transcript and protein name, functional category (see **Fig 2b**), regulation (UNIQUE or INCREASED), fold-difference in N-SC between egg quality groups (if available), and fraction in which the protein was originally detected (LMW, low molecular weight; HMW, high molecular weight) is shown. Color shading corresponds to that used to designate functional categories in **Fig 2**.

	ENSEMBL Protein ID	Asociated Gene Name	Associated Transcript Name	Protein Full Name	Functional Category	Regulation	Fold Difference	Fraction
1	ENSDARP00000043192	si:ch211-161h7.6	si:ch211-161h7.6-002	cst14a.2	Other	PQ UNIQUE	-	LMW
2	ENSDARP00000121287	si:ch211-161h7.6	si:ch211-161h7.6-001	cst14a.2	Other	PQ UNIQUE	-	LMW
3	ENSDARP00000053567	dap1b	dap1b-201	Death associated protein 1b	Apoptosis related	PQ UNIQUE	-	LMW
4	ENSDARP00000109203	dap1b	dap1b-202	Death associated protein b-apopotolic signaling pathway	Apoptosis related	PQ UNIQUE	-	LMW
5	ENSDARP00000022120	rab7	rab7-001	RAB7, member RAS oncogene family	Endosome-Lysosome related	PQ UNIQUE	-	LMW
6	ENSDARP00000113451	si:dkeyp-20e4.8	si:dkeyp-20e4.8-001	Uncharacterized protein	Other	PQ UNIQUE	-	LMW
7	ENSDARP00000069977	rpl23	rpl23-001	Ribosomal protein L23	Protein synthesis	PQ UNIQUE	-	LMW
8	ENSDARP00000112856	rpl30	rpl30-002	Ribosomal protein L30	Protein synthesis	PQ UNIQUE	-	LMW
9	ENSDARP00000052081	rpl30	rpl30-001	Ribosomal protein L30	Protein synthesis	PQ UNIQUE	-	LMW
10	ENSDARP00000025539	Rab7-like, rab7b,	Rab7-like, rab7b,	Rab7-like, rab7b	Endosome-Lysosome related	PQ UNIQUE	-	LMW
11	ENSDARP00000106613	Rab7-like	Rab7-like	Rab7-like	Endosome-Lysosome related	PQ UNIQUE	-	LMW
12	ENSDARP00000118883	apom	apom-002	Apolipoprotein M	Immune system related	PQ UNIQUE	-	LMW
13	ENSDARP00000103118	zgc:100918	Novel transcript	Uncharacterized protein-Rab7-like, ras-related, protein	Endosome-Lysosome related	PQ UNIQUE	-	LMW
14	ENSDARP00000111138	zgc:100918	Novel transcript	Uncharacterized protein-Rab7a-like, ras-related, protein	Endosome-Lysosome related	PQ UNIQUE	-	LMW
15	ENSDARP00000122132	apom	apom-003	Apolipoprotein M	Immune system related	PQ UNIQUE	-	LMW
16	ENSDARP00000016540	rab11al	rab11al-001	RAB11a, member RAS oncogene family, like	Endosome-Lysosome related	PQ UNIQUE	-	LMW
17	ENSDARP00000123136	apom	apom-001	Apolipoprotein M	Immune system related	PQ UNIQUE	-	LMW
18	ENSDARP00000128683	ACTC1 (2 of 2)	ACTC1 (2 of 2)-001	Actin, alpha, cardiac muscle 1a	Cell cycle, division, growth and fate	PQ UNIQUE	-	LMW
19	ENSDARP00000030257	si:ch211-226h8.8	si:ch211-226h8.8-201	Uncharacterized protein containing SUEL type lectin domain	Lectins	PQ UNIQUE	-	LMW
20	ENSDARP00000036607	fkbp1aa	fkbp1aa-001	FK506 binding protein 1Aa	Protein synthesis	PQ UNIQUE	-	LMW
21	ENSDARP00000119718	fkbp1aa	fkbp1aa-003	FK506 binding protein 1Aa	Protein synthesis	PQ UNIQUE	-	LMW
22	ENSDARP00000123671	fkbp1aa	fkbp1aa-002	FK506 binding protein 1Aa	Protein synthesis	PQ UNIQUE	-	LMW
23	ENSDARP00000075918	sri	sri-001	Sorcin	Protein degradation and synthesis inhibition	PQ UNIQUE	-	LMW
24	ENSDARP00000113274	si:ch211-226h8.8	si:ch211-226h8.8-001	Uncharacterized protein-Gal lectin superfamily	Lectins	PQ UNIQUE	-	LMW
25	ENSDARP00000121868	zgc:172218	Novel transcript	Uncharacterized protein containing 3SUEL type lectin domains	Lectins	PQ UNIQUE	-	LMW
26	ENSDARP00000117390	si:ch211-226h8.11	si:ch211-226h8.11-001	L-rhamnose-binding lectin CSL3-like isoform X1	Lectins	PQ UNIQUE	-	LMW
27	ENSDARP00000037689	ITIH4	ITIH4-201	Inter-alpha-trypsin inhibitor heavy chain family member 4	Other	PQ UNIQUE	-	LMW
28	ENSDARP00000059193	snrpd2	snrpd2-001	Small nuclear ribonucleoprotein D2 polypeptide	Protein synthesis	PQ UNIQUE	-	LMW
29	ENSDARP00000069885	rpl31	rpl31-201	Ribosomal protein L31	Protein synthesis	PQ UNIQUE	-	LMW
30	ENSDARP00000123730	rpl31	rpl31-002	Ribosomal protein L31	Protein synthesis	PQ UNIQUE	-	LMW
31	ENSDARP00000124804	rpl31	rpl31-001	Ribosomal protein L31	Protein synthesis	PQ UNIQUE	-	LMW
32	ENSDARP00000116530	snx3	snx3-003	Sorting nexin 3	Endosome-Lysosome related	PQ UNIQUE	-	LMW
33	ENSDARP00000120388	actb2	actb2-002	Actin, beta 2	Cell cycle, division, growth and fate	PQ UNIQUE	-	LMW
34	ENSDARP00000116418	actb1	actb1-002	Actin, beta 1	Cell cycle, division, growth and fate	PQ UNIQUE	-	LMW
35	ENSDARP00000107711	CABZ01044980.1	CABZ01044980.1-201	Zona pellucida sperm-binding protein 3-like precursor	Zona Pellucida proteins	PQ UNIQUE	-	LMW
36	ENSDARP00000115342	park7	park7-002	Parkinson protein 7	REDOX/Detox related	PQ UNIQUE	-	LMW
37	ENSDARP00000012094	snx3	snx3-001	Sorting nexin 3	Endosome-Lysosome related	PQ UNIQUE	-	LMW
38	ENSDARP00000104879	cp	cp-002	Ceruloplasmin	REDOX/Detox related	PQ UNIQUE	-	LMW
39	ENSDARP00000106910	cp	cp-003	Ceruloplasmin	REDOX/Detox related	PQ UNIQUE	-	LMW
40	ENSDARP00000067432	ube2nb	ube2nb-001	Ubiquitin-conjugating enzyme E2Nb	Protein degradation and synthesis inhibition	PQ UNIQUE	-	LMW
41	ENSDARP00000038063	lect2l	lect2l-001	Leukocyte cell-derived chemotaxin 2 like	Immune system related	PQ UNIQUE	-	LMW
42	ENSDARP00000095287	cp	cp-202	Ceruloplasmin	REDOX/Detox related	PQ UNIQUE	-	LMW
43	ENSDARP00000119305	cdh1	cdh1-001	Cadherin 1, type 1, E-cadherin (epithelial)	Cell cycle, division, growth and fate	PQ UNIQUE	-	LMW
44	ENSDARP00000117259	MFAP4 (12 of 14)	MFAP4 (12 of 14)-201	Microfibrillar-associated protein 4	Cell cycle, division, growth and fate	PQ UNIQUE	-	LMW
45	ENSDARP00000088490	cdh1	cdh1-201	Cadherin 1, type 1, E-cadherin (epithelial)	Cell cycle, division, growth and fate	PQ UNIQUE	-	LMW
46	ENSDARP00000095285	cp	cp-201	Ceruloplasmin	REDOX/Detox related	PQ UNIQUE	-	LMW
47	ENSDARP00000117207	CU469531.2	CU469531.2-201	Small GTPase mediated signal transduction-protein transport	Other	PQ UNIQUE	-	LMW
48	ENSDARP00000129339	cdc42	cdc42-001	Cell division cycle 42	Cell cycle, division, growth and fate	PQ UNIQUE	-	LMW
49	ENSDARP00000062517	zgc:162356	zgc:162356-001	Glutathione S-transferase rho	REDOX/Detox related	PQ UNIQUE	-	LMW
50	ENSDARP00000052837	acta1a	acta1a-001	Actin alpha 1 a skeletal muscle	Cell cycle, division, growth and fate	PQ UNIQUE	-	LMW
51	ENSDARP00000055135	actc1b	actc1b-201	Actin, alpha, cardiac muscle 1b	Cell cycle, division, growth and fate	PQ UNIQUE	-	LMW
52	ENSDARP00000058628	acta1b	acta1b-001	Actin, alpha 1b, skeletal muscle	Cell cycle, division, growth and fate	PQ UNIQUE	-	LMW
53	ENSDARP00000022550	ywhaqb	ywhaqb-001	Tyrosine 3-monooxygenase/tryptophan 5-monooxygenase activation protein, theta polypeptide b	REDOX/Detox related	PQ UNIQUE	-	LMW

54	ENSDARP00000062382	ywhaqa	ywhaqa-001	Tyrosine 3-monooxygenase/tryptophan 5-monooxygenase activation protein, theta polypeptide b	REDOX/Detox related	PQ UNIQUE	-	LMW
55	ENSDARP00000074935	ywhag1	ywhag1-001	3-monooxygenase/tryptophan 5-monooxygenase activation protein, gamma polypeptide 1	Cell cycle, division, growth and fate	PQ UNIQUE	-	LMW
56	ENSDARP00000096898	ywhag2	ywhag2-001	3-monooxygenase/tryptophan 5-monooxygenase activation protein, gamma polypeptide 2	Cell cycle, division, growth and fate	PQ UNIQUE	-	LMW
57	ENSDARP00000040421	bhmt	bhmt-201	Betaine-homocysteine methyltransferase	Other	PQ UNIQUE	-	LMW
58	ENSDARP00000104071	f2	f2-002	Coagulation fcator II (thrombin)	Immune system related	PQ UNIQUE	-	LMW
59	ENSDARP00000052324	f2	f2-001	Coagulation fcator II (thrombin)	Immune system related	PQ UNIQUE	-	LMW
60	ENSDARP00000015293	cp	cp-001	Ceruloplasmin	REDOX/Detox related	PQ UNIQUE	-	LMW
61	ENSDARP00000109575	si:ch1073-263o8.2	si:ch1073-263o8.2-201	Uncharacterized protein	Other	PQ UNIQUE	-	LMW
62	ENSDARP00000125992	si:ch1073-263o8.2	si:ch1073-263o8.2-001	Uncharacterized protein	Other	PQ UNIQUE	-	LMW
63	ENSDARP00000102472	vtg4	vtg4-202	Vitellogenin 4	Vitellogenins	PQ UNIQUE	-	LMW
64	ENSDARP00000103251	qdprb2	qdprb2-201	Quinoid dihydropteridine reductase b2	REDOX/Detox related	PQ UNIQUE	-	LMW
65	ENSDARP00000104702	qdprb2	qdprb2-001	Quinoid dihydropteridine reductase b2	REDOX/Detox related	PQ UNIQUE	-	LMW
66	ENSDARP00000005402	zgc:173556	zgc:173556-201	Zona pellucida protein superfamily	Zona Pellucida proteins	PQ UNIQUE	-	HMW
67	ENSDARP00000010482	ybx1	ybx1-001	Y box binding protein 1	Protein synthesis	PQ UNIQUE	-	HMW
68	ENSDARP00000016831	cct6a	cct6a-201	Chaperonin containing TCP1, subunit 6A (zeta 1)	Protein synthesis	PQ UNIQUE	-	HMW
69	ENSDARP00000059040	h1m	h1m-201	Linker histone H1M	Cell cycle, division, growth and fate	PQ UNIQUE	-	HMW
70	ENSDARP00000061743	zp3a.1	zp3a.1-001	Zona pellucida glycoprotein 3a, tandem duplicate 1	Zona Pellucida proteins	PQ UNIQUE	-	HMW
71	ENSDARP00000067534	cap2	cap2-201	CAP, adenylate cyclase-associated protein, 2 (yeast)	Cell cycle, division, growth and fate	PQ UNIQUE	-	HMW
72	ENSDARP00000068662	zp3.2	zp3.2-202	Zona pellucida glycoprotein 3, tandem duplicate 2	Zona Pellucida proteins	PQ UNIQUE	-	HMW
73	ENSDARP00000068670	zgc:173556	zgc:173556-203	Zona pellucida protein superfamily	Zona Pellucida proteins	PQ UNIQUE	-	HMW
74	ENSDARP00000095894	si:dkey-90l23.2	Novel transcript	L-rhamnose-binding lectin CSL3-like isoform X2	Lectins	PQ UNIQUE	-	HMW
75	ENSDARP00000099884	ZP2 (3 of 4)	ZP2 (3 of 4)-201	Zona pellucida glycoprotein 2 (sperm receptor)	Zona Pellucida proteins	PQ UNIQUE	-	HMW
76	ENSDARP00000101517	ybx1	ybx1-201	Y box binding protein 1	Protein synthesis	PQ UNIQUE	-	HMW
77	ENSDARP00000105615	CU695117.1	CU695117.1-201	Zinc finger MYM-type protein 1	Protein synthesis	PQ UNIQUE	-	HMW
78	ENSDARP00000105815	zgc:173556	zgc:173556-001	Zona pellucida glycoprotein 3.1	Zona Pellucida proteins	PQ UNIQUE	-	HMW
79	ENSDARP00000107790	zp3a.1	zp3a.1-201	Zona pellucida glycoprotein 3a, tandem duplicate 1	Zona Pellucida proteins	PQ UNIQUE	-	HMW
80	ENSDARP00000107948	CU467646.1	CU467646.1-201	Mucin-5B-like isoform X5	Other	PQ UNIQUE	-	HMW
81	ENSDARP00000109119	zp3.2	zp3.2-201	Zona pellucida protein superfamily	Zona Pellucida proteins	PQ UNIQUE	-	HMW
82	ENSDARP00000109275	CU929506.1	CU929506.1-201	Zinc finger MYM-type protein 1	Protein synthesis	PQ UNIQUE	-	HMW
83	ENSDARP00000112608	cct6a	cct6a-002	Chaperonin containing TCP1, subunit 6A (zeta 1)	Protein synthesis	PQ UNIQUE	-	HMW
84	ENSDARP00000116487	zgc:165539	zgc:165539-002	Mucin-5AC	Other	PQ UNIQUE	-	HMW
85	ENSDARP00000118006	cct6a	cct6a-001	Chaperonin containing TCP1, subunit 6A (zeta 1)	Protein synthesis	PQ UNIQUE	-	HMW
86	ENSDARP00000118276	si:dkey-90l23.1	Novel transcript	SUEL type lectin domain	Lectins	PQ UNIQUE	-	HMW
87	ENSDARP00000021016	tubb2	Novel transcript	Tubulin beta 2	Cell cycle, division, growth and fate	PQ UNIQUE	-	HMW
88	ENSDARP00000055379	tubb5	tubb5-201	Tubulin beta 5	Cell cycle, division, growth and fate	PQ UNIQUE	-	HMW
89	ENSDARP00000095074	cnp	cnp-201	2',3'-cyclic nucleotide 3' phosphodiesterase	Cell cycle, division, growth and fate	PQ UNIQUE	-	HMW
90	ENSDARP00000102472	vtg4	vtg4-202	Vitellogenin 4	Vitellogenins	PQ UNIQUE	-	HMW
91	ENSDARP00000109625	tubb2	Novel transcript	Tubulin beta 2A chain	Cell cycle, division, growth and fate	PQ UNIQUE	-	HMW
92	ENSDARP00000110851	FP102463.1	FP102463.1-201	Tubulin	Cell cycle, division, growth and fate	PQ UNIQUE	-	HMW
93	ENSDARP00000119055	zgc:123194	zgc:123194-001	Tubulin	Cell cycle, division, growth and fate	PQ UNIQUE	-	HMW
94	ENSDARP00000062142	ARF5 (1 of 2)	ARF5 (1 of 2)-001	ADP-ribosylation factor 5	Endosome-Lysosome related	PQ INCREASED	7.06	LMW
95	ENSDARP00000094243	arf5	arf5-201	ADP-ribosylation factor 5	Endosome-Lysosome related	PQ INCREASED	7.06	LMW
96	ENSDARP00000116010	vtg7	vtg7-002	Vitellogenin 7	Vitellogenins	PQ INCREASED	7.00	LMW
97	ENSDARP00000115259	actb1	actb1-004	Actin beta 1	Cell cycle, division, growth and fate	PQ INCREASED	6.85	LMW
98	ENSDARP00000112639	actb1	actb1-005	Actin, beta 1	Cell cycle, division, growth and fate	PQ INCREASED	6.81	LMW
99	ENSDARP00000111487	rpl22	rpl22-001	Ribosomal protein L22	Protein synthesis	PQ INCREASED	5.90	LMW
100	ENSDARP00000067606	arf4a	arf4a-202	ADP-ribosylation factor 4a	Cell cycle, division, growth and fate	PQ INCREASED	5.16	LMW
101	ENSDARP00000067603	arf4a	arf4a-201	ADP-ribosylation factor 4a	Cell cycle, division, growth and fate	PQ INCREASED	5.10	LMW
102	ENSDARP00000067677	zgc:110339	zgc:110339-001	Uncharacterized	REDOX/Detox related	PQ INCREASED	4.24	LMW
103	ENSDARP00000046181	gstm3	gstm3-001	Glutathione S-transferase M3 (brain)	REDOX/Detox related	PQ INCREASED	3.20	LMW
104	ENSDARP00000054986	actb1	actb1-001	Actin beta 1	Cell cycle, division, growth and fate	PQ INCREASED	3.13	LMW
105	ENSDARP00000055193	actb2	actb2-007	Actin beta 2	Cell cycle, division, growth and fate	PQ INCREASED	3.13	LMW
106	ENSDARP00000122263	actb2	actb2-001	Actin beta 2	Cell cycle, division, growth and fate	PQ INCREASED	3.13	LMW
107	ENSDARP00000116310	ube2l3b	ube2l3b-002	Ubiquitin-conjugating enzyme E2L 3b	Protein degradation and synthesis inhibition	PQ INCREASED	3.07	LMW
108	ENSDARP00000035541	ube2l3a	ube2l3a-201	Ubiquitin-conjugating enzyme E2L 3a	Protein degradation and synthesis inhibition	PQ INCREASED	3.06	LMW
109	ENSDARP00000008064	ube2l3b	ube2l3b-003	Ubiquitin-conjugating enzyme E2L 3b	Protein degradation and synthesis inhibition	PQ INCREASED	3.05	LMW
110	ENSDARP00000090376	ube2l3b	ube2l3b-001	Ubiquitin-conjugating enzyme E2L 3b	Protein degradation and synthesis inhibition	PQ INCREASED	3.05	LMW
111	ENSDARP00000032643	zgc:56530	zgc:56530-001	MGC174082 protein-mRNA	Other	PQ INCREASED	3.04	LMW
112	ENSDARP00000035513	sumo3a	sumo3a-001	Small ubiquitin-related modifier 3-like	Cell cycle, division, growth and fate	PQ INCREASED	2.73	LMW
113	ENSDARP00000052255	sumo3b	sumo3b-001	Small ubiquitin-related modifier	Cell cycle, division, growth and fate	PQ INCREASED	2.73	LMW
114	ENSDARP00000108738	sumo3a	sumo3a-201	Small ubiquitin-related modifier 3-like	Cell cycle, division, growth and fate	PQ INCREASED	2.73	LMW
115	ENSDARP00000010242	sumo2b	sumo2b-201	Small ubiquitin-related modifier 2	Cell cycle, division, growth and fate	PQ INCREASED	2.71	LMW

116	ENSDARP00000066628	sumo2b	sumo2b-001	Small ubiquitin-related modifier 2	Cell cycle, division, growth and fate	PQ INCREASED	2.71	LMW
117	ENSDARP00000006783	cfl2	cfl2-001	Cofilin	Cell cycle, division, growth and fate	PQ INCREASED	2.69	LMW
118	ENSDARP00000062369	actc1a	actc1a-001	Actin, alpha, cardiac muscle 1a	Cell cycle, division, growth and fate	PQ INCREASED	2.54	LMW
119	ENSDARP00000066429	acta2	acta2-201	Actin, alpha 2, smooth muscle, aorta	Cell cycle, division, growth and fate	PQ INCREASED	2.54	LMW
120	ENSDARP00000075110	CH211-260D9.2-001	Novel transcript	Actin, novel	Cell cycle, division, growth and fate	PQ INCREASED	2.54	LMW
121	ENSDARP00000100195	ACTC1 (2 of 2)	ACTC1 (2 of 2)-002	Actin alpha 1 (ACTA1)-like	Cell cycle, division, growth and fate	PQ INCREASED	2.54	LMW
122	ENSDARP00000100434	si:ch73-187m15.4	Novel transcript	Actin, alpha, cardiac muscle 1a, novel	Cell cycle, division, growth and fate	PQ INCREASED	2.54	LMW
123	ENSDARP00000009096	rpl12	rpl12-001	Ribosomal protein L12	Protein synthesis	PQ INCREASED	2.43	LMW
124	ENSDARP00000124371	acta2	acta2-001	Actin, alpha 2, smooth muscle, aorta	Cell cycle, division, growth and fate	PQ INCREASED	2.35	LMW
125	ENSDARP00000069198	nots	nots-201	Nothepsin	Protein degradation and synthesis inhibition	PQ INCREASED	2.27	LMW
126	ENSDARP00000119104	dut	dut-005	dUTP pyrophosphatase	Cell cycle, division, growth and fate	PQ INCREASED	2.20	LMW
127	ENSDARP00000122542	ddx41	ddx41-002	DEAD (Asp-Glu-Ala-Asp) box polypeptide 41	Cell cycle, division, growth and fate	PQ INCREASED	2.03	LMW
128	ENSDARP00000122730	ddx41	ddx41-001	DEAD (Asp-Glu-Ala-Asp) box polypeptide 41	Cell cycle, division, growth and fate	PQ INCREASED	2.03	LMW
129	ENSDARP00000118860	si:ch1073-75o15.4	si:ch1073-75o15.4-001	SUEL type lectin domain	Lectins	PQ INCREASED	1.94	LMW
130	ENSDARP00000121416	si:ch1073-75o15.4	si:ch1073-75o15.4-002	L-rhamnose-binding lectin CSL1-like isoform X1	Lectins	PQ INCREASED	1.93	LMW
131	ENSDARP00000120984	si:ch211-145c1.1	si:ch211-145c1.1-001	Gal lectin superfamily, L-rhamnose-binding lectin CSL3	Lectins	PQ INCREASED	6.88	HMW
132	ENSDARP00000093515	si:ch211-145c1.1	si:ch211-145c1.1-002	Gal lectin superfamily, L-rhamnose-binding lectin CSL3	Lectins	PQ INCREASED	6.85	HMW
133	ENSDARP00000020578	cap1	cap1-001	Adenylyl cyclase-associated protein	Cell cycle, division, growth and fate	PQ INCREASED	3.78	HMW
134	ENSDARP00000005460	c3b	c3b-201	Complement component c3b	Immune system related	PQ INCREASED	3.13	HMW
135	ENSDARP00000108724	c3b	c3b-001	Complement component c3b	Immune system related	PQ INCREASED	3.13	HMW
136	ENSDARP00000076290	pdia4	pdia4-201	Protein disulfide-isomerase A4	Protein synthesis	PQ INCREASED	2.99	HMW
137	ENSDARP00000027102	nap1l1	nap1l1-201	Nucleosome assembly protein 1, like 1	Cell cycle, division, growth and fate	PQ INCREASED	2.76	HMW
138	ENSDARP00000104033	A2ML1 (7 of 12)	A2ML1 (7 of 12)-201	Alpha-2-macroglobulin-like 1	Immune system related	PQ INCREASED	2.50	HMW
139	ENSDARP00000121031	hspa8	hspa8-003	Heat shock protein 8	Protein synthesis	PQ INCREASED	2.47	HMW
140	ENSDARP00000100695	A2ML1 (7 of 12)	A2ML1 (7 of 12)-001	Alpha-2-macroglobulin-like 1	Immune system related	PQ INCREASED	2.43	HMW
141	ENSDARP00000100381	A2ML1 (7 of 12)	A2ML1 (7 of 12)-203	Alpha-2-macroglobulin-like 1	Immune system related	PQ INCREASED	2.38	HMW
142	ENSDARP00000100196	A2ML1 (7 of 12)	A2ML1 (7 of 12)-202	Alpha-2-macroglobulin-like 1	Immune system related	PQ INCREASED	2.37	HMW
143	ENSDARP00000124860	nap1l4a	nap1l4a-002	Nucleosome assembly protein 1, like 1	Cell cycle, division, growth and fate	PQ INCREASED	2.25	HMW
144	ENSDARP00000094479	nap1l4a	nap1l4a-001	Nucleosome assembly protein 1, like 2	Cell cycle, division, growth and fate	PQ INCREASED	2.24	HMW
145	ENSDARP00000010384	prmt1	prmt1-001	Protein arginine methyltransferase 1	Cell cycle, division, growth and fate	PQ INCREASED	2.14	HMW
146	ENSDARP00000027115	taldo1	taldo1-201	Transaldolase	Energy metabolism	PQ INCREASED	2.00	HMW
147	ENSDARP00000067816	chia.3	chia.3-001	Chitinase, acidic.3	Immune system related	PQ INCREASED	2.00	HMW
148	ENSDARP00000103361	si:ch211-14a17.7	si:ch211-14a17.7-201	Zona pellucida protein superfamily	Zona Pellucida proteins	PQ INCREASED	2.00	HMW
149	ENSDARP00000116459	si:ch211-14a17.7	si:ch211-14a17.7-001	Zona pellucida protein superfamily	Zona Pellucida proteins	PQ INCREASED	2.00	HMW
150	ENSDARP00000003107	cct7	cct7-001	Chaperonin containing TCP1, subunit 7	Protein synthesis	PQ INCREASED	2.00	HMW
151	ENSDARP00000020361	gcat	gcat-001	Glycine C-acetyltransferase	Protein synthesis	PQ INCREASED	2.00	HMW
152	ENSDARP00000090833	prdx4	prdx4-001	Peroxiredoxin 4	REDOX/Detox related	GQ INCREASED	0.50	LMW
153	ENSDARP00000094194	vtg3	vtg3-202	Vitellogenin 3	Vitellogenins	GQ INCREASED	0.50	LMW
154	ENSDARP00000072678	vtg7	vtg7-201	Vitellogenin 7	Vitellogenins	GQ INCREASED	0.50	LMW
155	ENSDARP00000114558	vtg7	vtg7-001	Vitellogenin 7	Vitellogenins	GQ INCREASED	0.50	LMW
156	ENSDARP00000116066	celsr3	celsr3-002	Cadherin, EGF LAG seven-pass G-type receptor 3	Other	GQ INCREASED	0.50	LMW
157	ENSDARP00000118276	si:dkey-90l23.1	Novel transcript	SUEL type lectin domain	Lectins	GQ INCREASED	0.50	LMW
158	ENSDARP00000063876	prdx6	prdx6-001	Peroxiredoxin 6	REDOX/Detox related	GQ INCREASED	0.50	LMW
159	ENSDARP00000096079	si:ch211-226h8.4	si:ch211-226h8.4-001	SUEL type lectin domain	Lectins	GQ INCREASED	0.49	LMW
160	ENSDARP00000115012	vtg4	vtg4-001	Vitellogenin 4	Vitellogenins	GQ INCREASED	0.49	LMW
161	ENSDARP00000050237	vtg1	vtg1-001	Vitellogenin 1	Vitellogenins	GQ INCREASED	0.49	LMW
162	ENSDARP00000101309	vtg4	vtg4-201	Vitellogenin 4	Vitellogenins	GQ INCREASED	0.49	LMW
163	ENSDARP00000072738	vtg1	vtg1-201	Vitellogenin 1	Vitellogenins	GQ INCREASED	0.48	LMW
164	ENSDARP00000072790	vtg2	vtg2-201	Vitellogenin 2	Vitellogenins	GQ INCREASED	0.47	LMW
165	ENSDARP00000096034	vtg2	vtg2-202	Vitellogenin 2	Vitellogenins	GQ INCREASED	0.47	LMW
166	ENSDARP00000120934	prdx3	prdx3-002	Peroxiredoxin 3	REDOX/Detox related	GQ INCREASED	0.45	LMW
167	ENSDARP00000123406	si:dkeyp-46h3.8	si:dkeyp-46h3.8-001	SUEL type lectin domain	Lectins	GQ INCREASED	0.44	LMW
168	ENSDARP00000115568	si:dkeyp-46h3.8	si:dkeyp-46h3.8-201	SUEL type lectin domain	Lectins	GQ INCREASED	0.44	LMW
169	ENSDARP00000096443	crp6	crp6-001	C-reactive protein 6	Immune system related	GQ INCREASED	0.43	LMW
170	ENSDARP00000113089	dut	dut-003	dUTP pyrophosphatase	Cell cycle, division, growth and fate	GQ INCREASED	0.41	LMW
171	ENSDARP00000122570	dut	dut-002	dUTP pyrophosphatase	Cell cycle, division, growth and fate	GQ INCREASED	0.40	LMW
172	ENSDARP00000108320	dut	dut-001	dUTP pyrophosphatase-deoxyuridine triphosphatase	Cell cycle, division, growth and fate	GQ INCREASED	0.40	LMW
173	ENSDARP00000023863	rpe	rpe-001	Ribulose-5-phosphate-3-epimerase	Energy metabolism	GQ INCREASED	0.40	LMW
174	ENSDARP00000048374	stmn4	stmn4-001	Stathmin-like 4	Cell cycle, division, growth and fate	GQ INCREASED	0.39	LMW
175	ENSDARP00000113219	stmn4	stmn4-002	Stathmin-like 4	Cell cycle, division, growth and fate	GQ INCREASED	0.39	LMW
176	ENSDARP00000023158	vtg3	vtg3-201	Vitellogenin 3	Vitellogenins	GQ INCREASED	0.38	LMW
177	ENSDARP00000120407	si:ch211-250e5.16	si:ch211-250e5.16-001	SUEL type lectin domain	Lectins	GQ INCREASED	0.36	LMW

178	ENSDARP00000128406	vtg3	vtg3-001	Vitellogenin 3	Vitellogenins	GQ INCREASED	0.36	LMW
179	ENSDARP00000032532	ran	ran-003	GTP-binding nuclear protein Ran-Ras related nuclear protein	Cell cycle, division, growth and fate	GQ INCREASED	0.35	LMW
180	ENSDARP00000108629	ran	ran-001	RAN, member RAS oncogene family	Cell cycle, division, growth and fate	GQ INCREASED	0.35	LMW
181	ENSDARP00000121831	ran	ran-002	RAN, member RAS oncogene family	Cell cycle, division, growth and fate	GQ INCREASED	0.35	LMW
182	ENSDARP00000090756	glo1	glo1-001	Glyoxalase 1-Lactoylglutathione lyase	Energy metabolism	GQ INCREASED	0.34	LMW
183	ENSDARP00000117771	eif5a2	eif5a2-004	Translation initiation factor	Protein synthesis	GQ INCREASED	0.33	LMW
184	ENSDARP00000073095	eif5a2	eif5a2-001	Translation initiation factor	Protein synthesis	GQ INCREASED	0.33	LMW
185	ENSDARP00000117947	eif5a2	eif5a2-002	Translation initiation factor	Protein synthesis	GQ INCREASED	0.33	LMW
186	ENSDARP00000120991	eif5a2	eif5a2-003	Translation initiation factor	Protein synthesis	GQ INCREASED	0.33	LMW
187	ENSDARP00000117883	si:dkey-88l16.3	si:dkey-88l16.3-001	Low-density lipoprotein receptor-related protein 2 isoform X5	Lipid metabolism	GQ INCREASED	0.30	LMW
188	ENSDARP00000056381	crp3	crp3-201	C-reactive protein 2	Immune system related	GQ INCREASED	0.23	LMW
189	ENSDARP00000104927	crp3	crp3-202	C-reactive protein 3	Immune system related	GQ INCREASED	0.23	LMW
190	ENSDARP00000116589	crp3	crp3-001	C-reactive protein 3	Immune system related	GQ INCREASED	0.23	LMW
191	ENSDARP00000053425	pfn2l	pfn2l-001	Profilin 2 like	Cell cycle, division, growth and fate	GQ INCREASED	0.16	LMW
192	ENSDARP00000113417	pfn2l	pfn2l-002	Profilin 2 like	Cell cycle, division, growth and fate	GQ INCREASED	0.16	LMW
193	ENSDARP00000128438	pfn2l	pfn2l-003	Profilin 2 like	Cell cycle, division, growth and fate	GQ INCREASED	0.16	LMW
194	ENSDARP00000089804	zgc:136254	zgc:136254-001	Fish egg lectin like isoform X1	Endosome-Lysosome related	GQ INCREASED	0.16	LMW
195	ENSDARP00000129497	zgc:136254	zgc:136254-201	Fish egg lectin like isoform X1	Endosome-Lysosome related	GQ INCREASED	0.16	LMW
196	ENSDARP00000070002	aldh6a1	aldh6a1-001	Aldehyde dehydrogenase 6 family, member A1	Energy metabolism	GQ INCREASED	0.55	HMW
197	ENSDARP00000116781	si:dkey-152b24.6	si:dkey-152b24.6-001	1-phosphatidylinositol phosphodiesterase-like	Lipid metabolism	GQ INCREASED	0.54	HMW
198		si:ch1073-13h15.3	si:ch1073-13h15.3-001	Putative all-trans-retinol 13,14-reductase	Other	GQ INCREASED	0.54	HMW
199	ENSDARP00000024082	atic	atic-001	5-aminoimidazole-4-carboxamide ribonucleotide formyltransferase/IMP cyclohydrolase	Cell cycle, division, growth and fate	GQ INCREASED	0.54	HMW
200	ENSDARP00000008576	A2ML1 (1 of 12)	A2ML1 (1 of 12)-001	Alpha-2-macroglobulin-like 1	Immune system related	GQ INCREASED	0.53	HMW
201	ENSDARP00000069178	si:ch1073-13h15.3	si:ch1073-13h15.3-201	Putative all-trans-retinol 13,14-reductase	Other	GQ INCREASED	0.53	HMW
202	ENSDARP00000014978	hsp90ab1	hsp90ab1-001	Heat shock protein 90, alpha (cytosolic), class B member 1	Protein synthesis	GQ INCREASED	0.50	HMW
203	ENSDARP00000025538	ranbp1	ranbp1-001	RAN binding protein 1	Other	GQ INCREASED	0.50	HMW
204	ENSDARP00000060204	tufm	tufm-001	Elongation factor Tu	Protein synthesis	GQ INCREASED	0.50	HMW
205	ENSDARP00000101406	ranbp1	ranbp1-201	RAN binding protein 1	Other	GQ INCREASED	0.50	HMW
206	ENSDARP00000104512	hsp90ab1	hsp90ab1-201	Heat shock protein 90, alpha (cytosolic), class B member 1	Protein synthesis	GQ INCREASED	0.50	HMW
207	ENSDARP00000025702	tfa	tfa-201	Transferrin a	Immune system related	GQ INCREASED	0.49	HMW
208	ENSDARP00000012767	aldh2.1	aldh2.1-001	Aldehyde dehydrogenase 2, tandem duplicate 1	Lipid metabolism	GQ INCREASED	0.49	HMW
209	ENSDARP00000100241	tfa	tfa-001	Transferrin a	Immune system related	GQ INCREASED	0.49	HMW
210	ENSDARP00000062351	SLC25A6 (1 of 2)	SLC25A6 (1 of 2)-201	Solute carrier family 25 (mitochondrial carrier; adenine nucleotide translocator), member 6	Cell cycle, division, growth and fate	GQ INCREASED	0.46	HMW
211	ENSDARP00000096509	SLC25A6 (1 of 2)	SLC25A6 (1 of 2)-001	Solute carrier family 25 (mitochondrial carrier; adenine nucleotide translocator), member 6	Cell cycle, division, growth and fate	GQ INCREASED	0.46	HMW
212	ENSDARP00000008085	setb	setb-001	SET translocation (myeloid leukemia-associated) B	Cell cycle, division, growth and fate	GQ INCREASED	0.46	HMW
213	ENSDARP00000066176	tuba2	tuba2-001	Tubulin alpha 2	Cell cycle, division, growth and fate	GQ INCREASED	0.45	HMW
214	ENSDARP00000022077	slc25a6	slc25a6-001	Solute carrier family 25 (mitochondrial carrier; adenine nucleotide translocator), member 6	Cell cycle, division, growth and fate	GQ INCREASED	0.45	HMW
215	ENSDARP00000030881	slc25a4	slc25a4-001	Solute carrier family 25 (mitochondrial carrier; adenine nucleotide translocator), member 6	Cell cycle, division, growth and fate	GQ INCREASED	0.45	HMW
216	ENSDARP00000058383	gapdhs	gapdhs-001	Glyceraldehyde-3-phosphate dehydrogenase, spermatogenic	Energy metabolism	GQ INCREASED	0.45	HMW
217	ENSDARP00000120922	decr1	decr1-001	2,4-dienoyl CoA reductase 1, mitochondrial	Energy metabolism	GQ INCREASED	0.45	HMW
218	ENSDARP00000023429	aldh2.2	aldh2.2-001	Aldehyde dehydrogenase 2, tandem duplicate 1	Energy metabolism	GQ INCREASED	0.45	HMW
219	ENSDARP00000088020	aldh2.2	aldh2.2-202	Aldehyde dehydrogenase 2, tandem duplicate 1	Energy metabolism	GQ INCREASED	0.45	HMW
220	ENSDARP00000088019	aldh2.2	aldh2.2-201	Aldehyde dehydrogenase 2, tandem duplicate 1	Energy metabolism	GQ INCREASED	0.44	HMW
221	ENSDARP00000117350	gale	gale-001	UDP-galactose-4-epimerase	Energy metabolism	GQ INCREASED	0.42	HMW
222	ENSDARP00000011034	gale	gale-201	UDP-galactose-4-epimerase	Energy metabolism	GQ INCREASED	0.41	HMW
223	ENSDARP00000073345	A2ML1 (1 of 12)	A2ML1 (1 of 12)-201	Alpha-2-macroglobulin-like 1	Immune system related	GQ INCREASED	0.40	HMW
224	ENSDARP00000116143	esd	esd-002	Esterase D/formylglutathione hydrolase	Other	GQ INCREASED	0.37	HMW
225	ENSDARP00000118149	esd	esd-003	Esterase D/formylglutathione hydrolase	Other	GQ INCREASED	0.37	HMW
226	ENSDARP00000024121	esd	esd-001	Esterase D/formylglutathione hydrolase	Other	GQ INCREASED	0.36	HMW
227	ENSDARP00000014456	vdac2	vdac2-001	Voltage-dependent anion channel 2	Cell cycle, division, growth and fate	GQ INCREASED	0.34	HMW
228	ENSDARP00000111790	vdac2	vdac2-201	Voltage-dependent anion channel 2	Cell cycle, division, growth and fate	GQ INCREASED	0.34	HMW
229	ENSDARP00000114537	pgk1	pgk1-003	Phosphoglycerate kinase 1	Energy metabolism	GQ INCREASED	0.34	HMW
230	ENSDARP00000119690	akr1a1b	akr1a1b-004	Aldo-keto reductase family 1, member A1a (aldehyde reductase)	Lipid metabolism	GQ INCREASED	0.32	HMW
231	ENSDARP00000113696	akr1a1b	akr1a1b-005	Aldo-keto reductase family 1, member A1a (aldehyde reductase)	Lipid metabolism	GQ INCREASED	0.32	HMW
232	ENSDARP00000120539	akr1a1b	akr1a1b-006	Aldo-keto reductase family 1, member A1a (aldehyde reductase)	Lipid metabolism	GQ INCREASED	0.32	HMW
233	ENSDARP00000096504	slc25a5	slc25a5-001	Solute carrier family 25 (mitochondrial carrier; adenine nucleotide translocator), member 6	Cell cycle, division, growth and fate	GQ INCREASED	0.30	HMW
234	ENSDARP00000040408	ppa1b	ppa1b-001	Phospholysine phosphohistidine inorganic pyrophosphate phosphatase	Energy metabolism	GQ INCREASED	0.28	HMW
235	ENSDARP00000034804	st13	st13-201	Suppression of tumorigenicity 13 (colon carcinoma) (Hsp70 interacting protein)	Protein synthesis	GQ INCREASED	0.28	HMW
236	ENSDARP00000112549	st13	st13-001	Suppression of tumorigenicity 13 (colon carcinoma) (Hsp70 interacting protein)	Protein synthesis	GQ INCREASED	0.28	HMW
237	ENSDARP00000070807	pgk1	pgk1-001	Phosphoglycerate kinase 1	Energy metabolism	GQ INCREASED	0.27	HMW
238	ENSDARP00000124008	aldh6a1	aldh6a1-003	Aldehyde dehydrogenase 6 family, member A1	Energy metabolism	GQ INCREASED	0.25	HMW
239	ENSDARP00000123894	aldh6a1	aldh6a1-002	Aldehyde dehydrogenase 6 family, member A1	Energy metabolism	GQ INCREASED	0.25	HMW

240	ENSDARP00000070667	zgc:171779	zgc:171779-201	Zona pellucida superfamily	Cell cycle, division, growth and fate	GQ INCREASED	0.24	HMW
241	ENSDARP00000006510	pgm1	pgm1-001	Phosphoglucomutase 1	Energy metabolism	GQ INCREASED	0.10	HMW
242	ENSDARP00000113349	KRT23 (1 of 2)	KRT23 (1 of 2)-003	Keratin 17	Cell cycle, division, growth and fate	GQ UNIQUE	-	LMW
243	ENSDARP00000089806	si:ch211-251f6.7	si:ch211-251f6.7-201	Fish egg lectin like precursor	Lectins	GQ UNIQUE	-	LMW
244	ENSDARP00000113985	si:ch211-251f6.7	si:ch211-251f6.7-001	Fish egg lectin like precursor	Lectins	GQ UNIQUE	-	LMW
245	ENSDARP00000115840	dut	dut-004	Deoxyuridine triphosphatase	Cell cycle, division, growth and fate	GQ UNIQUE	-	LMW
246	ENSDARP00000059013	zgc:173443	zgc:173443-001	Fish egg lectin like precursor	Lectins	GQ UNIQUE	-	LMW
247	ENSDARP00000034555	cycsb	cycsb-001	cytochrome c, somatic b	Energy metabolism	GQ UNIQUE	-	LMW
248	ENSDARP00000037864	glrx	glrx-001	Glutaredoxin (thioltransferase)	Protein synthesis	GQ UNIQUE	-	LMW
249	ENSDARP00000076087	glrx	glrx-201	Glutaredoxin (thioltransferase)	Protein synthesis	GQ UNIQUE	-	LMW
250	ENSDARP00000120838	glrx	glrx-002	Glutaredoxin (thioltransferase)	Protein synthesis	GQ UNIQUE	-	LMW
251	ENSDARP00000121692	gstm3	gstm3-002	Glutathione S transferase M3 (brain)	REDOX/Detox related	GQ UNIQUE	-	LMW
252	ENSDARP00000051749	si:rp71-45k5.4	si:rp71-45k5.4-001	Proteasome subunit alpha 2	Protein degradation and synthesis inhibition	GQ UNIQUE	-	LMW
253	ENSDARP00000128459	si:rp71-45k5.4	si:rp71-45k5.4-202	Proteasome subunit alpha 2	Protein degradation and synthesis inhibition	GQ UNIQUE	-	LMW
254	ENSDARP00000058679	psma2	psma2-201	Proteasome subunit alpha 2	Protein degradation and synthesis inhibition	GQ UNIQUE	-	LMW
255	ENSDARP00000127697	si:rp71-45k5.4	si:rp71-45k5.4-201	Proteasome subunit alpha 2	Protein degradation and synthesis inhibition	GQ UNIQUE	-	LMW
256	ENSDARP00000075482	sb:cb252	sb:cb252-201	ES1 protein homolog, mitochondrial	Energy metabolism	GQ UNIQUE	-	LMW
257	ENSDARP00000027268	gspt1l	gspt1l-001	G1 to S phase transition 1, like	Protein synthesis	GQ UNIQUE	-	HMW
258	ENSDARP00000039367	prmt1	prmt1-002	Protein arginine methyltransferase 1	Cell cycle, division, growth and fate	GQ UNIQUE	-	HMW
259	ENSDARP00000052324	f2	f2-001	Coagulation factor II, thrombin	Immune system related	GQ UNIQUE	-	HMW
260	ENSDARP00000057644	qsox1	qsox1-001	Quiescin Q6 sulfhydryl oxidase 1	Protein synthesis	GQ UNIQUE	-	HMW
261	ENSDARP00000096969	psmc5	psmc5-001	Proteasome (prosome, macropain) 26S subunit, ATPase, 5	Protein synthesis	GQ UNIQUE	-	HMW
262	ENSDARP00000104071	f2	f2-002	Coagulation factor II, thrombin	Immune system related	GQ UNIQUE	-	HMW
263	ENSDARP00000105266	zgc:152830	zgc:152830-201	Peptidase M17 superfamily	Protein degradation and synthesis inhibition	GQ UNIQUE	-	HMW
264	ENSDARP00000108730	tfa	tfa-202	Transferrin a	Immune system related	GQ UNIQUE	-	HMW
265	ENSDARP00000110443	BX323793.2	BX323793.2-201	1-phosphatidylinositol phosphodiesterase-like	Lipid metabolism	GQ UNIQUE	-	HMW
266	ENSDARP00000111409	si:dkey-152b24.7	si:dkey-152b24.7-201	1-phosphatidylinositol phosphodiesterase-like	Lipid metabolism	GQ UNIQUE	-	HMW
267	ENSDARP00000112306	pycr1b	pycr1b-201	Pyrroline-5-carboxylate reductase 1b	Protein degradation and synthesis inhibition	GQ UNIQUE	-	HMW
268	ENSDARP00000116484	gspt1l	gspt1l-002	G1 to S phase transition 1, like	Protein synthesis	GQ UNIQUE	-	HMW
269	ENSDARP00000116563	si:dkey-152b24.7	si:dkey-152b24.7-001	1-phosphatidylinositol phosphodiesterase-like	Lipid metabolism	GQ UNIQUE	-	HMW
270	ENSDARP00000117331	nasp	nasp-002	Nuclear autoantigenic sperm protein (histone-binding)	Protein synthesis	GQ UNIQUE	-	HMW
271	ENSDARP00000118947	gale	gale-002	UDP-galactose-4-epimerase	Energy metabolism	GQ UNIQUE	-	HMW
272	ENSDARP00000127771	zgc:152830	zgc:152830-001	Peptidase M17 superfamily	Protein degradation and synthesis inhibition	GQ UNIQUE	-	HMW
273	ENSDARP00000128522	tfa	tfa-003	Transferrin a	Immune system related	GQ UNIQUE	-	HMW
274	ENSDARP00000017776	A2ML1 (10 of 12)	A2ML1 (10 of 12)-201	Alpha-2-macroglobulin-like 1	Lipid metabolism	GQ UNIQUE	-	HMW
