# Supplementary material for: Scrambled eggs: Proteomic portraits and novel biomarkers of egg quality in zebrafish (Danio rerio)
Source: PLoS One. 2017 Nov 16;12(11):e0188084. doi: 10.1371/journal.pone.0188084 (PMC5690628; doi:10.1371/journal.pone.0188084)
Supplement: S3 Table — (PDF) [file pone.0188084.s003.pdf]

S3 Table. Enrichment of PANTHER pathways with differentially regulated proteins. Results of overrepresentation binomial tests [27] for enrichment of PANTHER pathways with proteins showing a ≥2-fold difference in N-SC between egg quality groups, or unique to an egg quality group, in the Pooled Samples Experiment (top) and in the Multiple Samples Experiment (bottom). Only statistically significant results ($p \leq 0.05$ after Bonferroni correction for multiple tests) are reported. Numbers of mapped and unmapped proteins are given in **Fig 3 and Fig 5**.

**PANTHER Overrepresentation Test**<br>Analyzed List: *Danio rerio* (input - differentially regulated proteins)	PANTHER version 11.0 Released 2016<br>Reference List: *Danio rerio* (all genes in database)					
**POOLED SAMPLES EXPERIMENT**	**POOR QUALITY EGGS**					
**PANTHER Pathways**	REFLIST (27187)	Input (194)	Input (expected)	Input (over/under)	Input (fold Enrichment)	Input (P-value)
Cytoskeletal regulation by Rho GTPase (P00016)	120	12	0.86	+	14.01	1.67E-08
Parkinson disease (P00049)	112	11	0.8	+	13.76	1.22E-07
Huntington disease (P00029)	183	13	1.31	+	9.96	1.69E-07
Cadherin signaling (P00012)	183	12	1.31	+	9.19	1.79E-06
FGF signaling (P00021)	160	10	1.14	+	8.76	4.69E-05
Nicotinic acetylcholine receptor signaling (P00044)	141	8	1.01	+	7.95	1.46E-03
EGF receptor signaling (P00018)	174	9	1.24	+	7.25	8.46E-04
Alzheimer disease-presenilin (P00004)	166	8	1.18	+	6.75	4.65E-03
Integrin signaling (P00034)	219	9	1.56	+	5.76	5.10E-03
Wnt signaling (P00057)	388	13	2.77	+	4.7	8.32E-04
Inflammation mediated by chemokine /cytokine signaling (P00031)	353	10	2.52	+	3.97	3.94E-02
Unclassified (UNCLASSIFIED)	24075	146	171.79	-	0.85	0.00E+00
	**GOOD QUALITY EGGS**					
**PANTHER Pathways**	REFLIST (27187)	Input (251)	Input (expected)	Input (over/under)	Input (fold Enrichment)	Input (P-value)
Pyruvate metabolism (P02772)	15	6	0.14	+	43.33	1.26E-06
De novo purine biosynthesis (P02738)	33	4	0.3	+	13.13	4.22E-02
Unclassified (UNCLASSIFIED)	24075	188	222.27	-	0.85	0.00E+00
**MULTIPLE SAMPLES EXPERIMENT**	**POOR QUALITY EGGS**					
**PANTHER Pathways**	REFLIST (27187)	Input (74)	Input (expected	Input (over/under)	Input (fold Enrichment)	Input (P-value)
Cytoskeletal regulation by Rho GTPase (P00016)	120	15	0.33	+	45.92	1.03E-18
Huntington disease (P00029)	183	17	0.5	+	34.13	2.92E-19
Nicotinic acetylcholine receptor signaling pathway (P00044)	141	10	0.38	+	26.06	1.14E-09
Alzheimer disease-presenilin pathway (P00004)	166	11	0.45	+	24.35	1.98E-10
Cadherin signaling pathway (P00012)	183	11	0.5	+	22.08	5.57E-10
Parkinson disease (P00049)	112	6	0.3	+	19.68	1.09E-04
Integrin signaling pathway (P00034)	219	7	0.6	+	11.74	3.78E-04
Inflammation med. by chemokine & cytokine signaling (P00031)	353	10	0.96	+	10.41	7.02E-06
Wnt signaling pathway (P00057)	388	9	1.06	+	8.52	1.80E-04
Unclassified (UNCLASSIFIED)	24075	43	65.53	-	0.66	0.00E+00
	**GOOD QUALITY EGGS**					
**PANTHER Pathways**	REFLIST (27187)	Input (60)	Input (expected)	Input (over/under)	Input (fold Enrichment)	Input (P-value)
5-Hydroxytryptamine degradation (P04372)	19	3	0.04	+	71.54	1.73E-03
Unclassified (UNCLASSIFIED)	24075	43	53.13	-	0.81	0.00E+00
