# Supplementary material for: Scrambled eggs: Proteomic portraits and novel biomarkers of egg quality in zebrafish (Danio rerio)
Source: PLoS One. 2017 Nov 16;12(11):e0188084. doi: 10.1371/journal.pone.0188084 (PMC5690628; doi:10.1371/journal.pone.0188084)
Supplement: S2 Fig — (PDF) [file pone.0188084.s005.pdf]

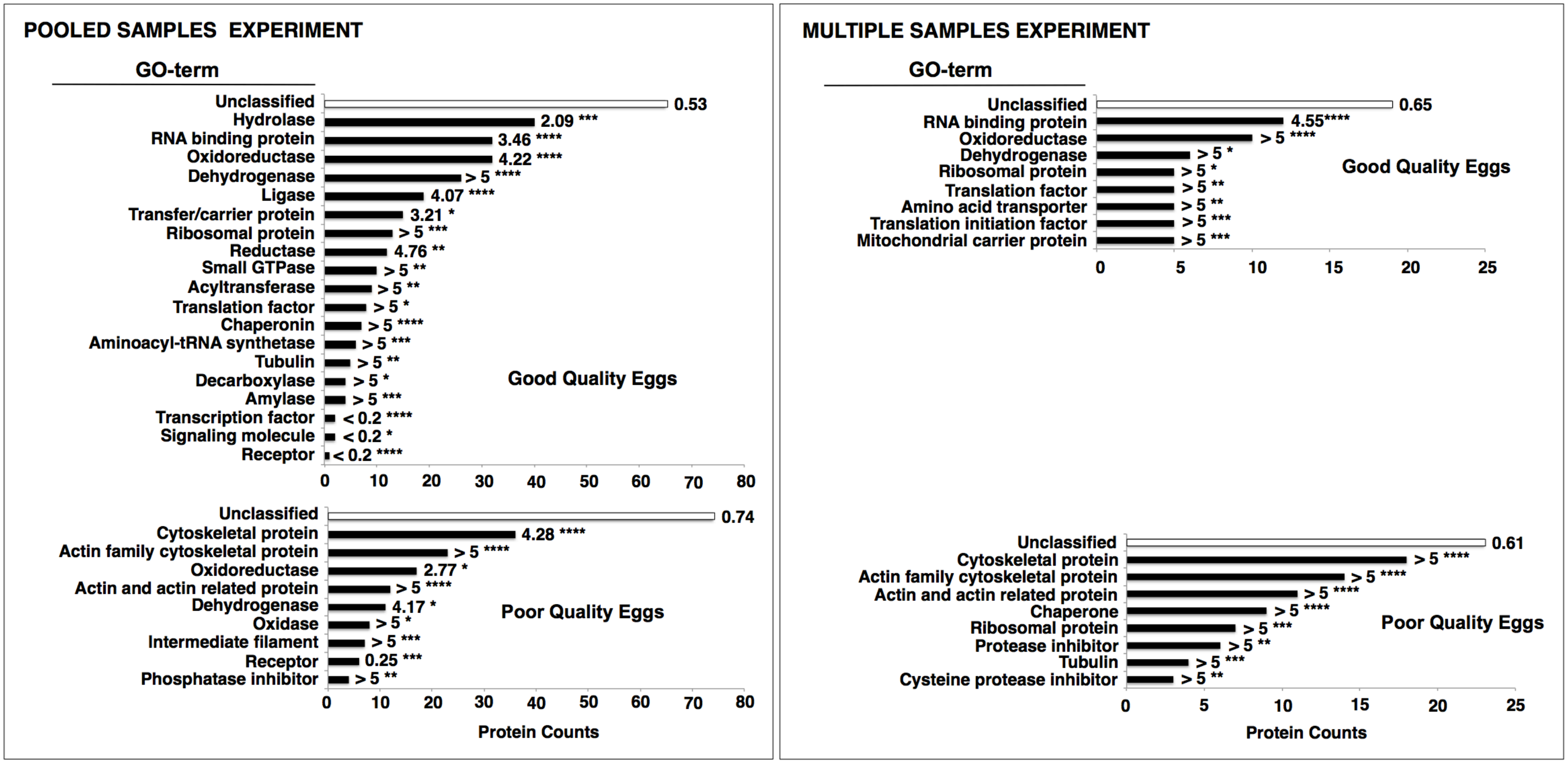

S2 Fig. Enrichment of Protein Class GO terms with differentially regulated proteins. Results of over-representation binomial tests [27] for enrichment of Protein Class GO terms (PANTHER GO-Slim) with proteins up-regulated in good and poor quality zebrafish eggs. **Left Panel**. Pooled Samples Experiment. **Right Panel**. Multiple Samples Experiment. Horizontal bars indicate the number of proteins attributed to each GO term for which statistically significant results ($p \leq 0.05$ after Bonferroni correction for multiple tests) were observed. Numbers next to the bars indicate the fold-enrichment with proteins attributed to each term and the number of asterisks indicates the significance level of the enrichment, as follows $p \leq 0.05$ (*), $p \leq 0.01$ (**), $p \leq 0.001$ (***), and $p \leq 0.0001$ (****).
