# Supplementary material for: Scrambled eggs: Proteomic portraits and novel biomarkers of egg quality in zebrafish (Danio rerio)
Source: PLoS One. 2017 Nov 16;12(11):e0188084. doi: 10.1371/journal.pone.0188084 (PMC5690628; doi:10.1371/journal.pone.0188084)
Supplement: S4 Fig — (PDF) [file pone.0188084.s007.pdf]

S4 Fig. Diagrams of PANTHER pathways enriched with differentially regulated proteins. Proteins showing a ≥2-fold difference in N-SC between egg quality groups, or unique to an egg quality group, in the Pooled Samples Experiment and/or the Multiple Samples Experiment are mapped to the PANTHER pathways in which they were found to be overrepresented (see **S3 Table**). Pathways are presented as follows:

Panel a. Cytoskeletal regulation by Rho GTPase pathway (PANTHER pathway P00016)
Panel b. Parkinson disease pathway (PANTHER pathway P00049)
Panel c. Huntington's disease pathway (PANTHER pathway P00029)
Panel d. Cadherin signaling pathway (PANTHER pathway P00012)
Panel e. FGF signaling pathway (PANTHER pathway P00021)
Panel f. Nicotinic acetylcholine receptor signaling pathway (PANTHER pathway P00044)
Panel g. EGF receptor signaling pathway (PANTHER pathway P00018)
Panel h. Alzheimer's disease-presenilin pathway (PANTHER pathway P00004)
Panel i. Integrin signaling pathway (PANTHER pathway P00034)
Panel j. Wnt signaling pathway (PANTHER pathway P00057).
Panel k. Inflammation mediated by chemokine & cytokine signaling pathway (PANTHER pathway P00031)
Panel l. Pyruvate metabolism pathway (PANTHER pathway P02772)
Panel m. De novo purine biosynthesis (PANTHER pathway P02738)
Panel n. 5-Hydroxytryptamine degradation (PANTHER pathway P04372)

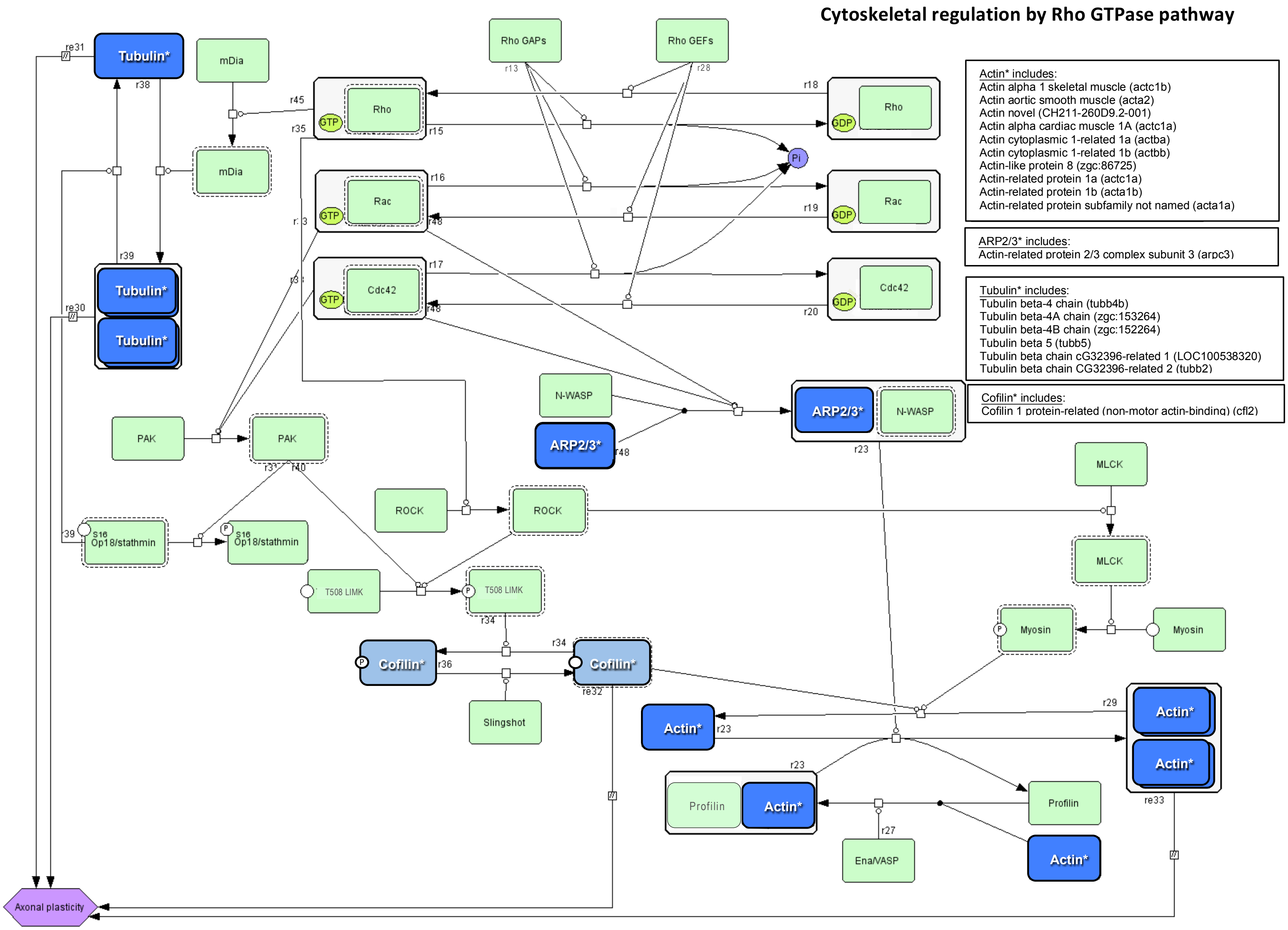

Panel a. Proteins up-regulated ≥ 2-fold in poor quality zebrafish eggs, or unique to poor quality eggs, mapped to the Cytoskeletal regulation by Rho GTPase pathway (PANTHER pathway P00016) in which they were found to be overrepresented (see **S3 Table**). The zebrafish proteins are indicated by blue boxes with white text and are individually named in open boxes to the upper right with corresponding transcript names in parentheses. Dark blue boxes indicate mapped proteins from the Pooled Samples Experiment and light blue boxes indicate additional mapped proteins from the Multiple Samples Experiment. The mapped zebrafish proteins include actins (Actin*), actin-related protein 2/3 complex subunit 3 (ARP2/3*), tubulins (Tubulin*), and cofilin 1 protein-related protein.

Parkinson disease pathway

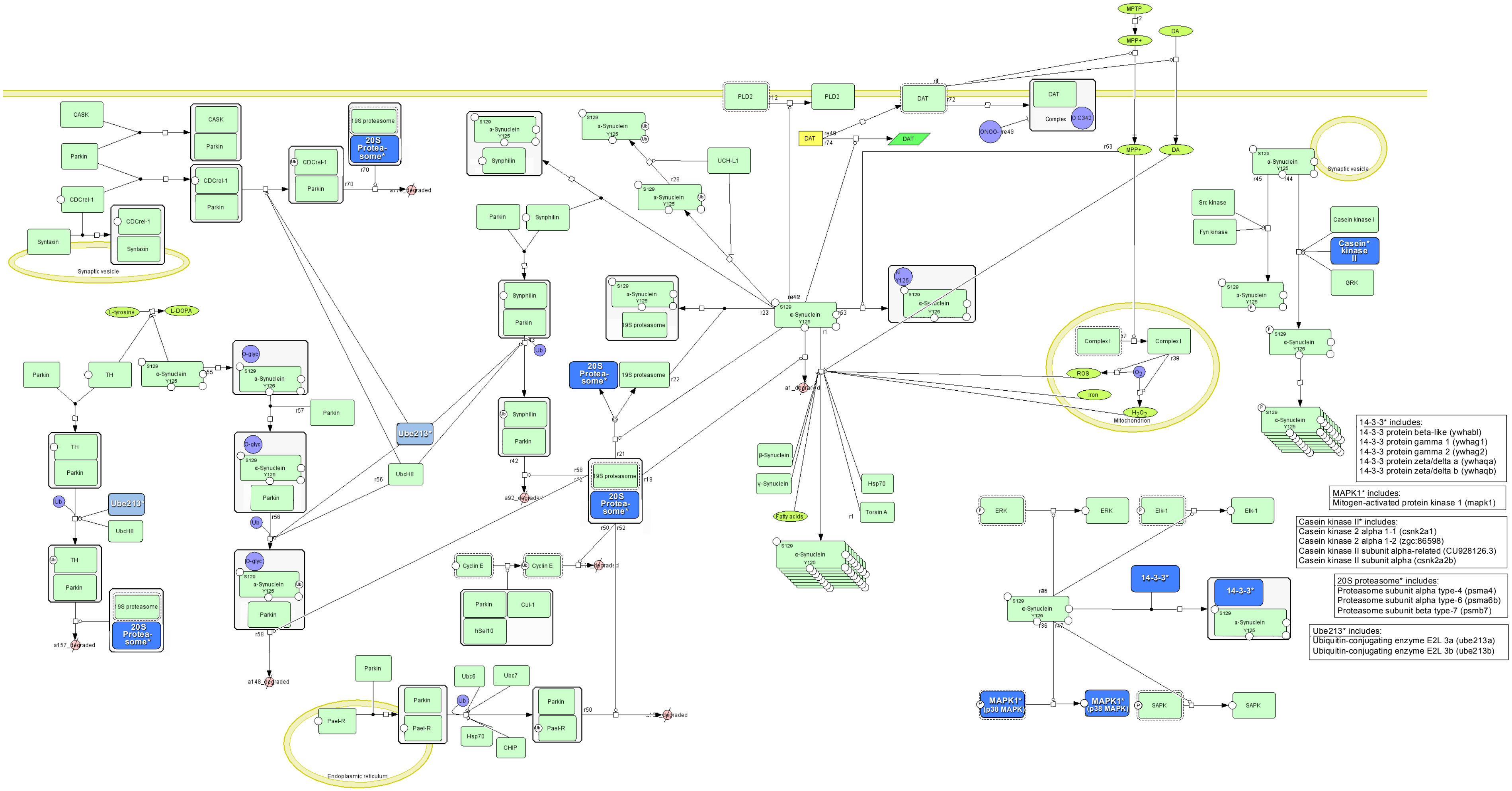

Panel b. Proteins up-regulated $\geq$ 2-fold in poor quality zebrafish eggs, or unique to poor quality eggs, mapped to the Parkinson disease pathway (PANTHER pathway P00049) in which they were found to be overrepresented (see **S3 Table**). The zebrafish proteins are indicated by blue boxes with white text and are individually named in open boxes to the lower right with corresponding transcript names in parentheses. Dark blue boxes indicate mapped proteins from the Pooled Samples Experiment and light blue boxes indicate additional mapped proteins from the Multiple Samples Experiment. The mapped zebrafish proteins include 20S-proteasome components (20S proteasome*), variants of ubiquitin-conjugating enzyme E2L 3B (Ube213*), mitogen-activated kinase 1 (MAPK1*), variants of casein kinase II (Casein kinase II*), and 14-3-3 chaperone proteins (14-3-3*).

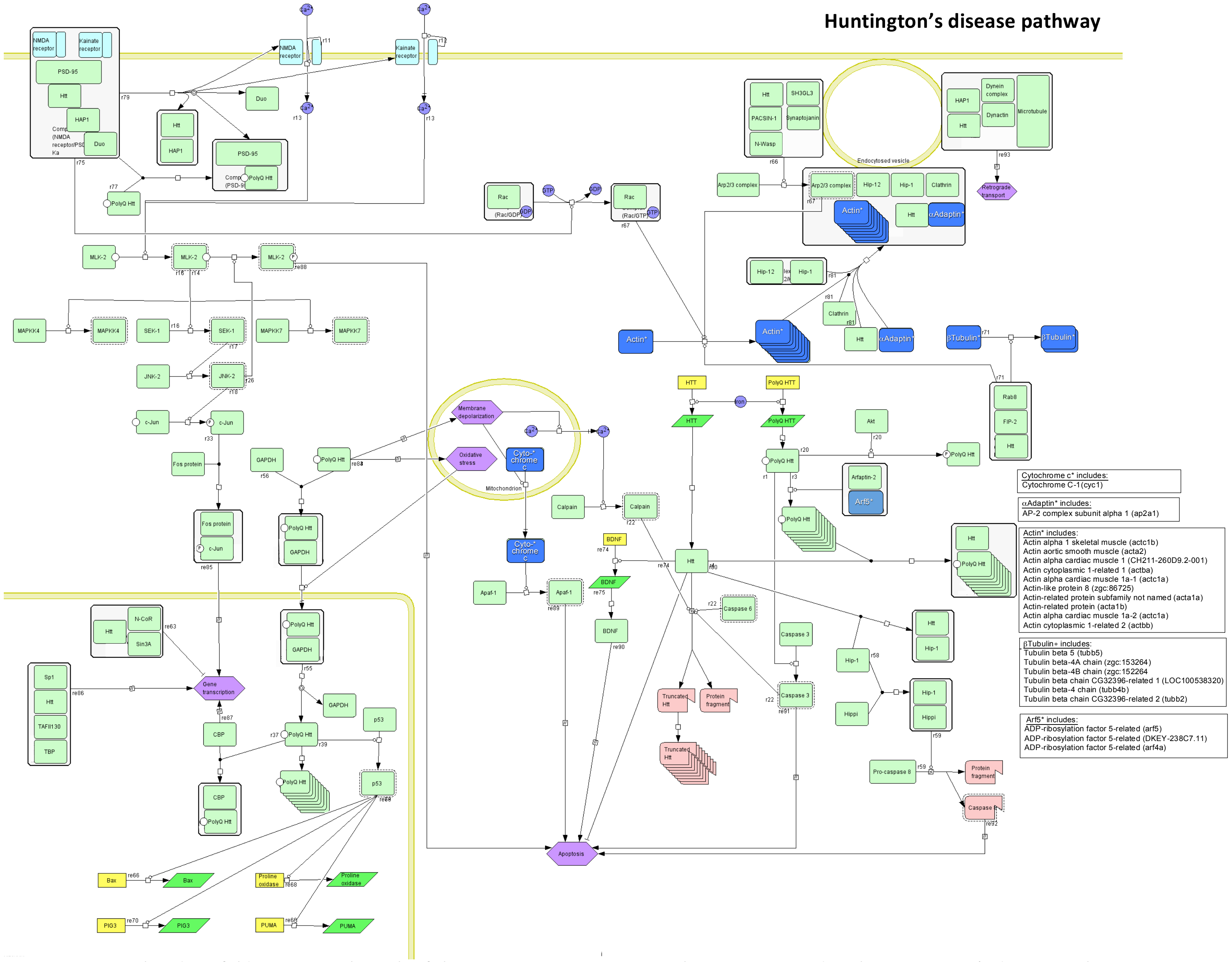

Panel c. Proteins up-regulated > 2-fold in poor quality zebrafish eggs, or unique to poor quality eggs, mapped to the Huntington's disease pathway (Panther pathway P00029) in which they were found to be overrepresented (see **S3 Table**). The zebrafish proteins are indicated by blue boxes with white text and are individually named in open boxes to the lower right with corresponding transcript names in parentheses. Dark blue boxes indicate mapped proteins from the Pooled Samples Experiment and light blue boxes indicate additional mapped proteins from the Multiple Samples Experiment. Aside from mitochondrial cytochrome c and variants of ADP-ribosylation factor 5, all of these zebrafish proteins are actins, tubulins or α-adaptin involved in clathrin-mediated endocytosis, a major activity in growing oocytes.

Cadherin signaling pathway

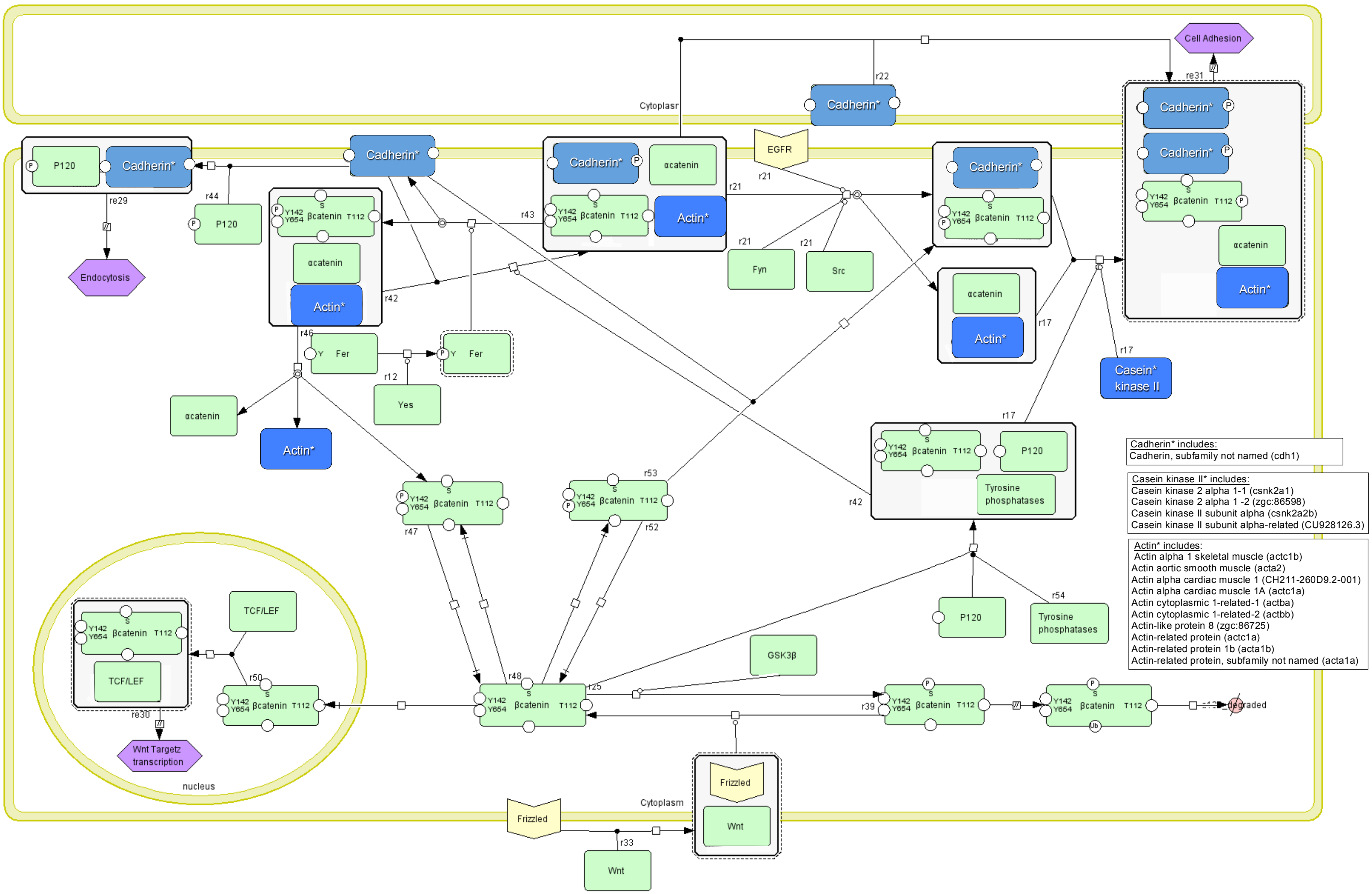

Panel d. Proteins upregulated > 2-fold in poor quality zebrafish eggs, or unique to poor quality eggs, mapped to the Cadherin signaling pathway (Panther pathway P00012) in which they were found to be overrepresented (see **S3 Table**). The zebrafish proteins are indicated by blue boxes with white text and are individually named in open boxes on the lower right with corresponding transcript names in parentheses. Dark blue boxes indicate mapped proteins from the Pooled Samples Experiment and light blue boxes indicate additional mapped proteins from the Multiple Samples Experiment. The mapped zebrafish proteins are all actins (Actin*), variants of casein kinase II (Casein kinase II*) or cadherin 1 (Cadherin*).

FGF signaling pathway

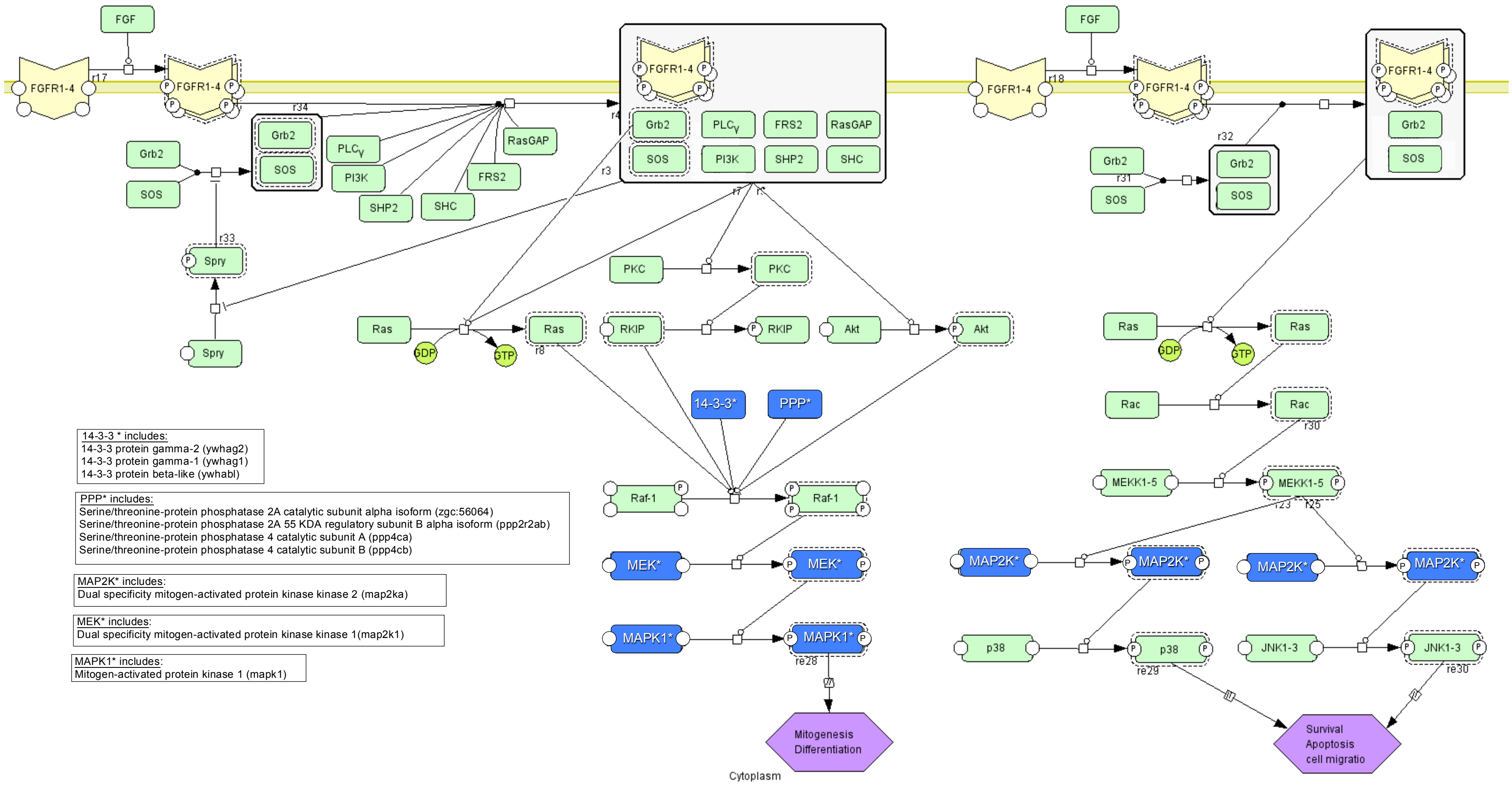

Panel e. Proteins up-regulated > 2-fold in poor quality zebrafish eggs, or unique to poor quality eggs, mapped to the FGF signaling pathway (Panther pathway P00021) in which they were found to be overrepresented (see **S3 Table**). The zebrafish proteins are indicated by blue boxes with white text and are individually named in open boxes on the lower left with corresponding transcript names in parentheses. The dark blue boxes indicate mapped proteins from the Pooled Samples Experiment. The mapped zebrafish proteins are all 14-3-3 chaperone proteins (14-3-3*), variants of serine/threonine protein phosphatase (PPP*), variants of dual specificity mitogen-activated protein kinase kinase (MEK* and MAP2K*), or mitogen-activated protein kinase 1 (MAPK1*) and all were also mapped to the EGF signaling pathway (see **S4 Fig Panel g**).

Nicotinic acetylcholine receptor signaling pathway

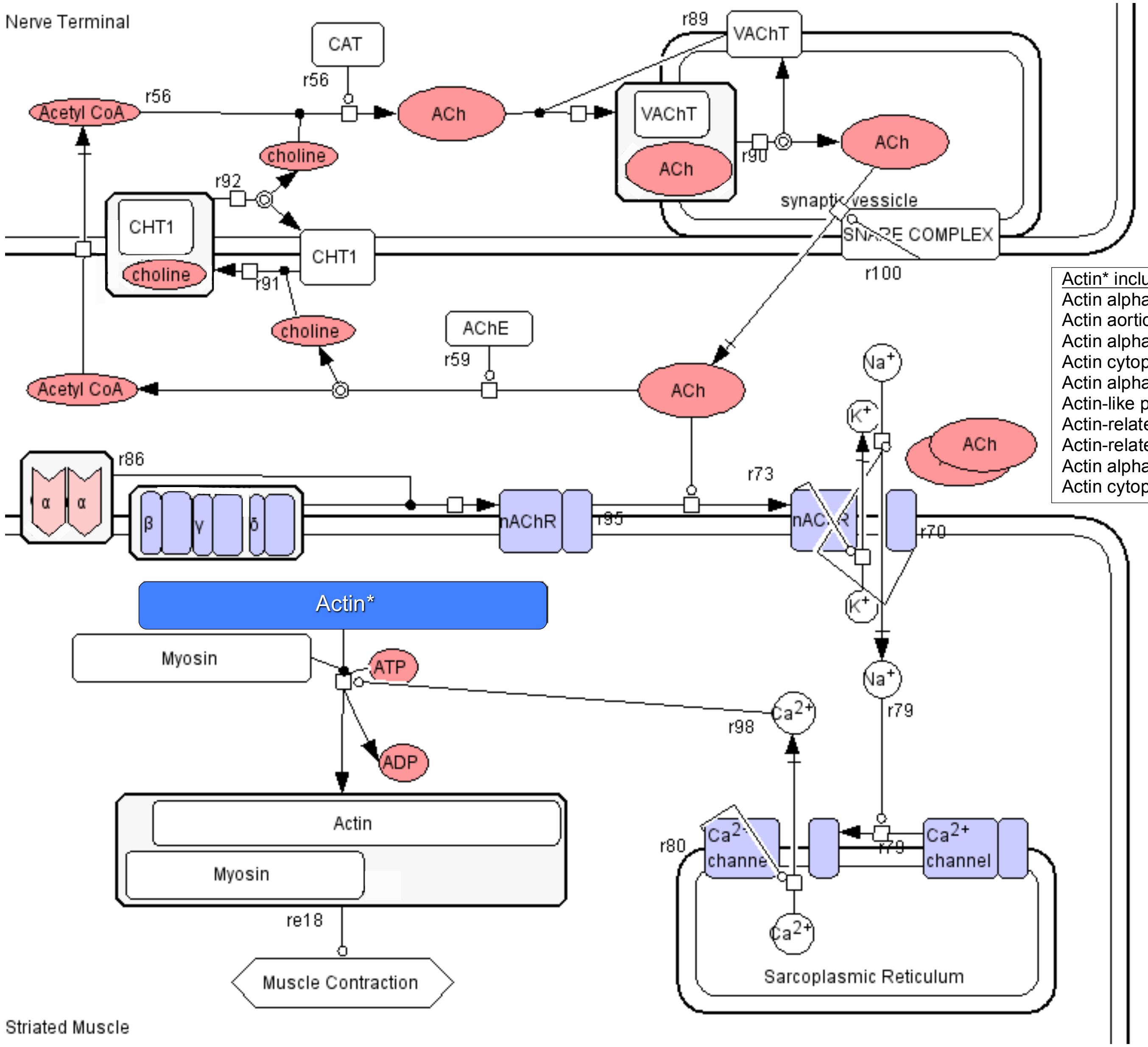

Actin* includes:
Actin alpha 1 skeletal muscle (actc1b)
Actin aortic smooth muscle (acta2)
Actin alpha cardiac muscle 1 (CH211-260D9.2-001)
Actin cytoplasmic 1-related 1 (actba)
Actin alpha cardiac muscle 1a-1 (actc1a)
Actin-like protein 8 (zgc:86725)
Actin-related protein subfamily not named (acta1a)
Actin-related protein (acta1b)
Actin alpha cardiac muscle 1a-2 (actc1a)
Actin cytoplasmic 1-related 2 (actbb)

Panel f. Proteins up-regulated $\geq$ 2-fold in poor quality zebrafish eggs, or unique to poor quality eggs, mapped to the Nicotinic acetylcholine receptor signaling pathway (Panther pathway P00044) in which they were found to be overrepresented (see **S3 Table**). The mapped zebrafish proteins from the Pooled Samples Experiment and the Multiple Samples Experiment are indicated by the blue box with white text and are individually named in open boxes on the right with corresponding transcript names in parentheses. Experiment. The mapped zebrafish proteins are all actins (Actin*).

EGF receptor signaling pathway

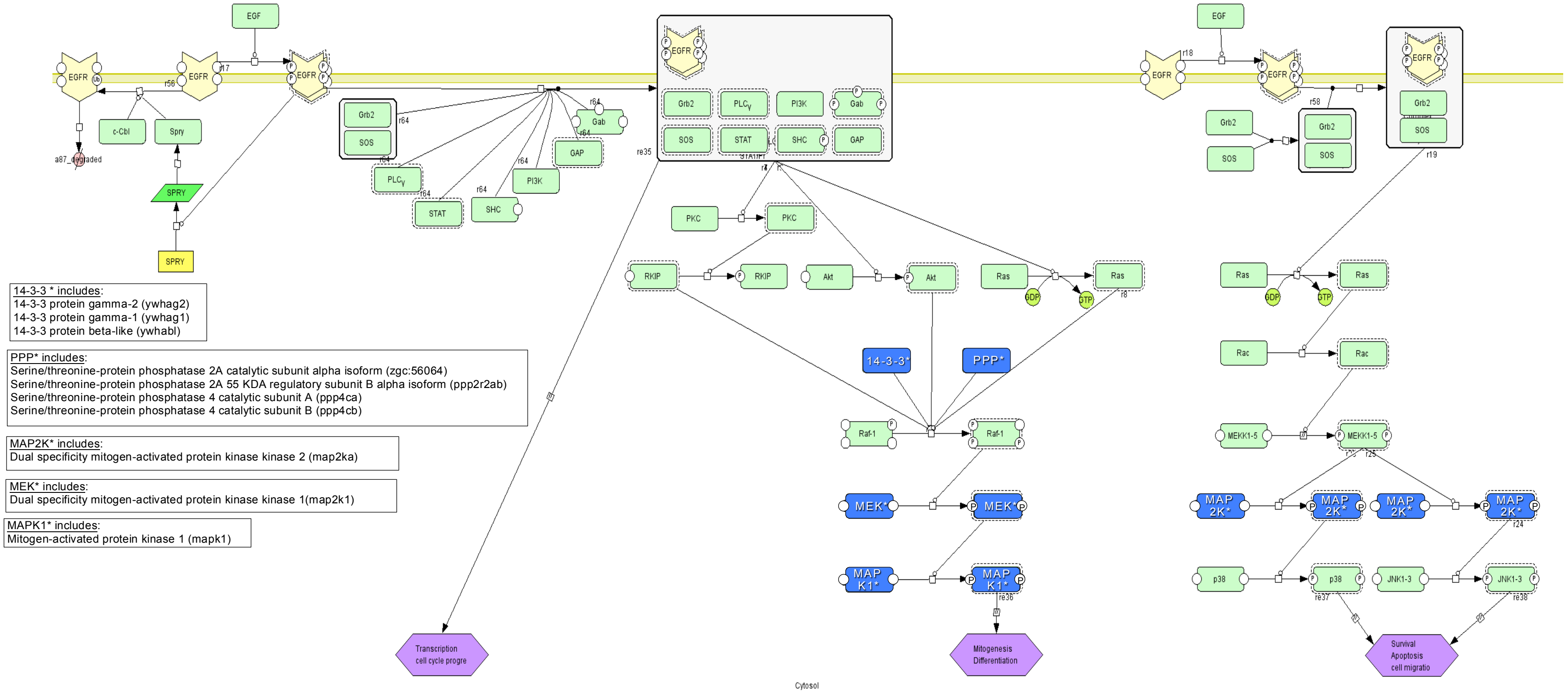

Panel g. Proteins up-regulated ≥ 2-fold in poor quality zebrafish eggs, or unique to poor quality eggs, mapped to the EGF receptor signaling pathway (Panther pathway P00018) in which they were found to be overrepresented (see **S3 Table**). The zebrafish proteins are indicated by the blue boxes with white text and are individually named in open boxes on the left with corresponding transcript names in parentheses. The dark blue boxes indicate mapped proteins from the Pooled Samples Experiment. The mapped zebrafish proteins are all 14-3-3 chaperone proteins (14-3-3*), variants of serine/threonine protein phosphatase (PPP*), variants of dual specificity mitogen-activated protein kinase kinase (MEK* and MAP2K*), or mitogen-activated protein kinase 1 (MAPK1*) and all were also mapped to the FGF signaling pathway (see **S4 Fig Panel e**).

Alzheimer's disease-presenilin pathway

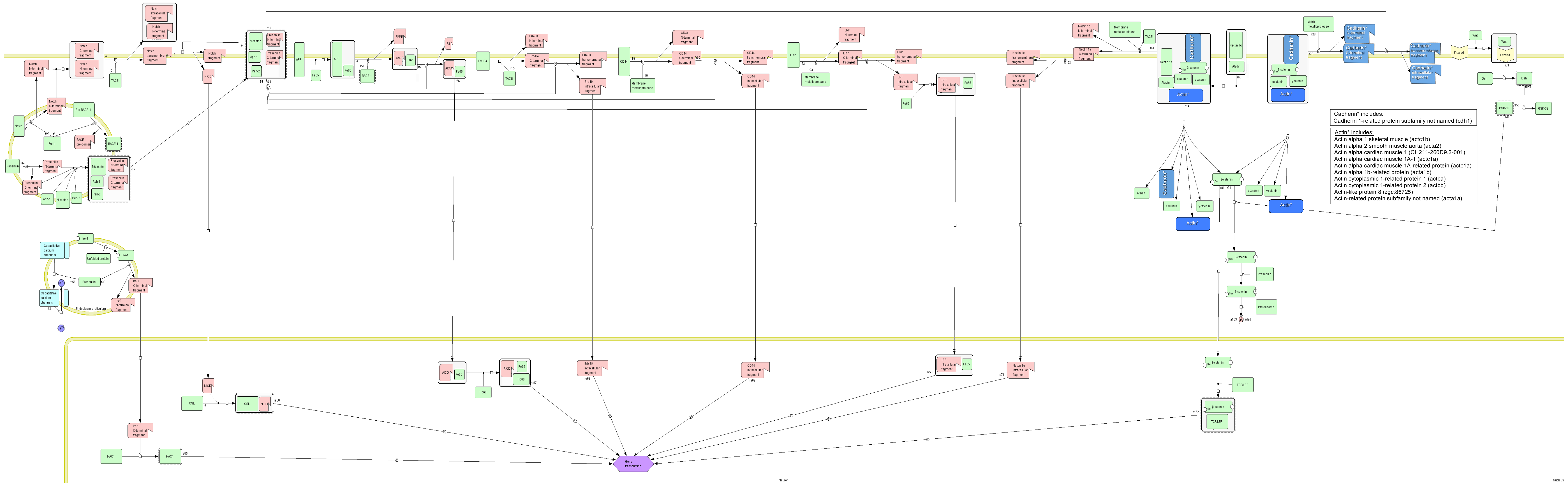

Panel h. Proteins up-regulated ≥ 2-fold in poor quality zebrafish eggs, or unique to poor quality eggs, mapped to the Alzheimer's disease-presenilin pathway (Panther pathway P00004) in which they were found to be overrepresented (see **S3 Table**). The zebrafish proteins are indicated by blue boxes with white text and are individually named in open boxes on the upper right with corresponding transcript names in parentheses. Dark blue boxes indicate mapped proteins from the Pooled Samples Experiment and light blue shapes indicate mapped proteins from the Multiple Samples Experiment. The mapped zebrafish proteins are all actins (Actin*) or cadherin (Cadherin*).

Integrin signaling pathway

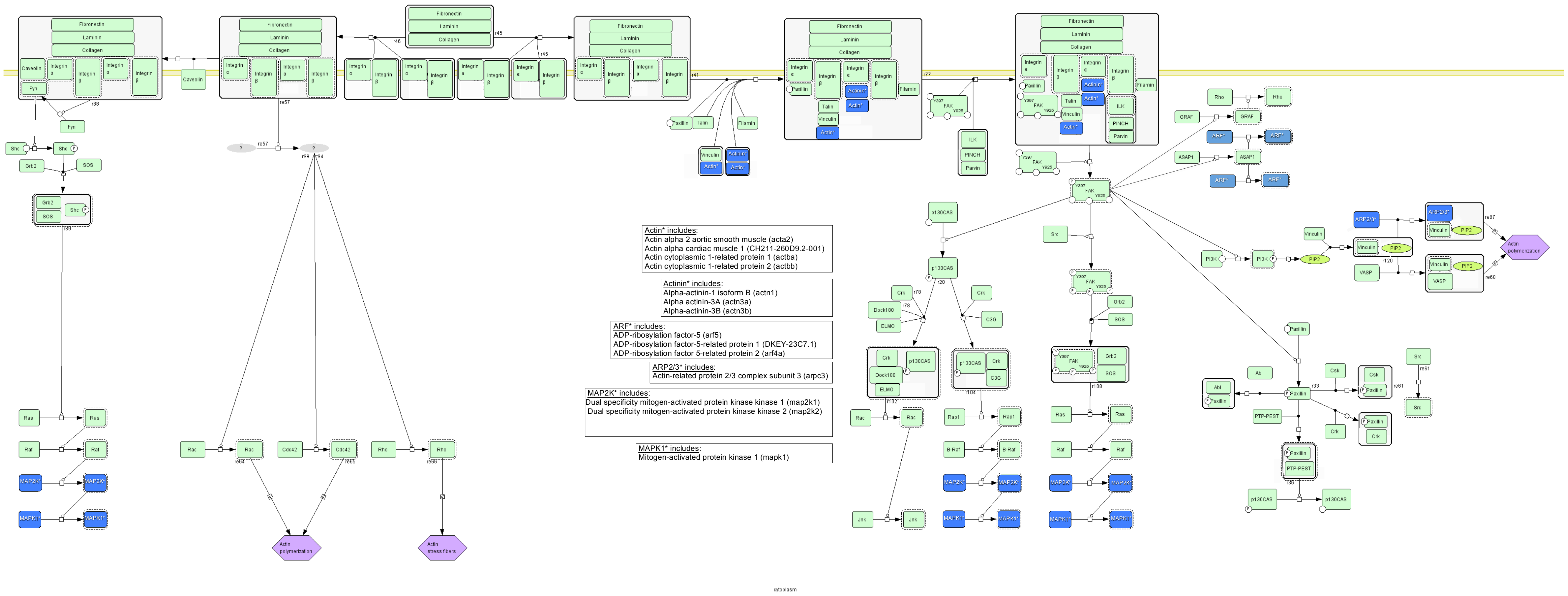

Panel i. Proteins up-regulated > 2-fold in poor quality zebrafish eggs, or unique to poor quality eggs, mapped to the Integrin signaling pathway (Panther pathway P00034) in which they were found to be overrepresented (see **S3 Table**). The zebrafish proteins are indicated by blue boxes with white text and are individually named in open boxes in the center with corresponding transcript names in parentheses. Dark blue boxes indicate mapped proteins from the Pooled Samples Experiment and light blue boxes indicate additional mapped proteins from the Multiple Samples Experiment. The mapped zebrafish proteins are actins (Actin*), actin-related proteins (ARP2/3*), actinins (Actinin*), ADP-ribosylation factor-5 variants (ARF*), dual-specificity mitogen-activated protein kinases (MAP2K*), or mitogen-activated protein kinase 1 (MAPK1*).

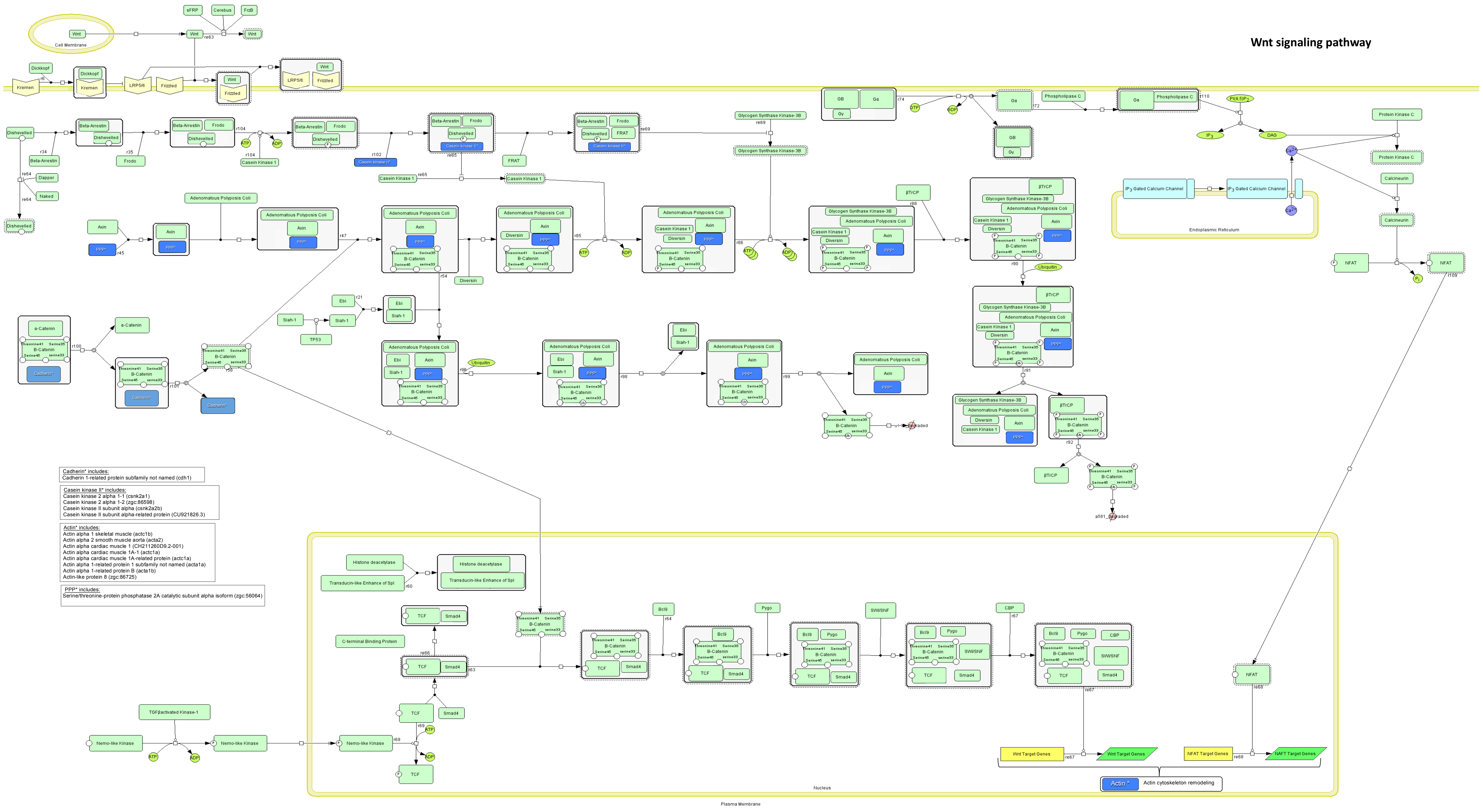

Panel j. Proteins up-regulated > 2-fold in poor quality zebrafish eggs, or unique to poor quality eggs, mapped to the Wnt signaling pathway (Panther pathway P00057) in which they were found to be overrepresented (see **S3 Table**). The zebrafish proteins are indicated by the blue boxes with white text and are individually named in open boxes on the left with corresponding transcript names in parentheses. The dark blue boxes indicate mapped proteins from the Pooled Samples Experiment and the light blue boxes indicate mapped proteins from the Multiple Samples Experiment. The mapped zebrafish proteins are cadherin-1-related protein (Cadherin*), variants of casein kinase II (Casein Kinase II*), actins (Actin*) or Serine/threonine-protein phosphatase 2A catalytic subunit alpha isoform (PPP*).

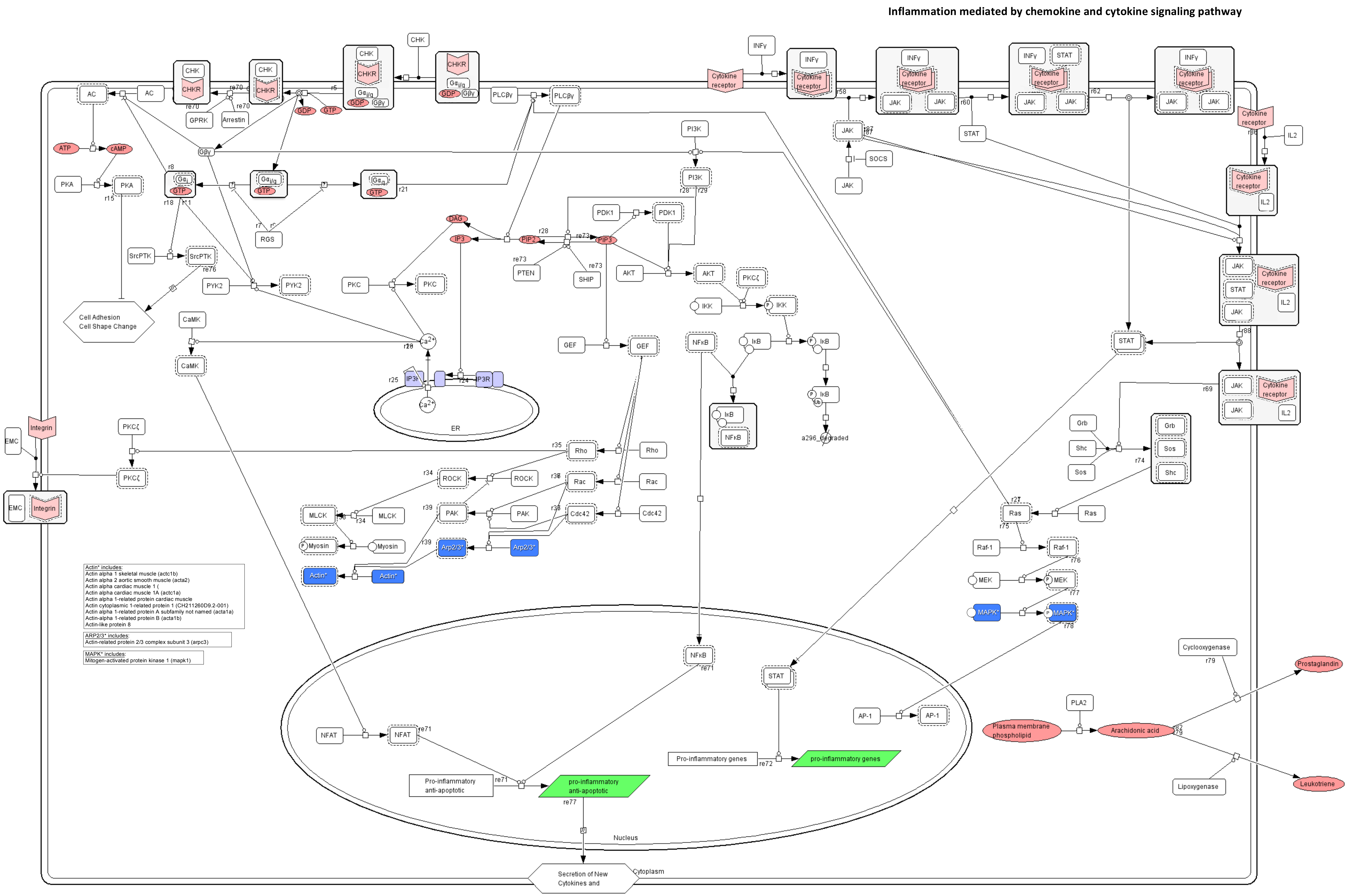

Panel k. Proteins up-regulated ≥ 2-fold in poor quality zebrafish eggs, or unique to poor quality eggs, mapped to Inflammation mediated by chemokine and cytokine signaling pathway (Panther pathway P00031) in which they were found to be overrepresented (see **S3 Table**). The zebrafish proteins are indicated by blue boxes with white text and are individually named in open boxes at the lower left with corresponding transcript names in parentheses. Dark blue boxes indicate mapped proteins from the Pooled Samples Experiment and the Multiple Samples Experiment. The mapped zebrafish proteins are actins (Actin*), actin-related protein 2/3 complex subunit 3 (Arp2/3*) and mitogen-activated protein kinase (MAPK*)

Pyruvate metabolism pathway

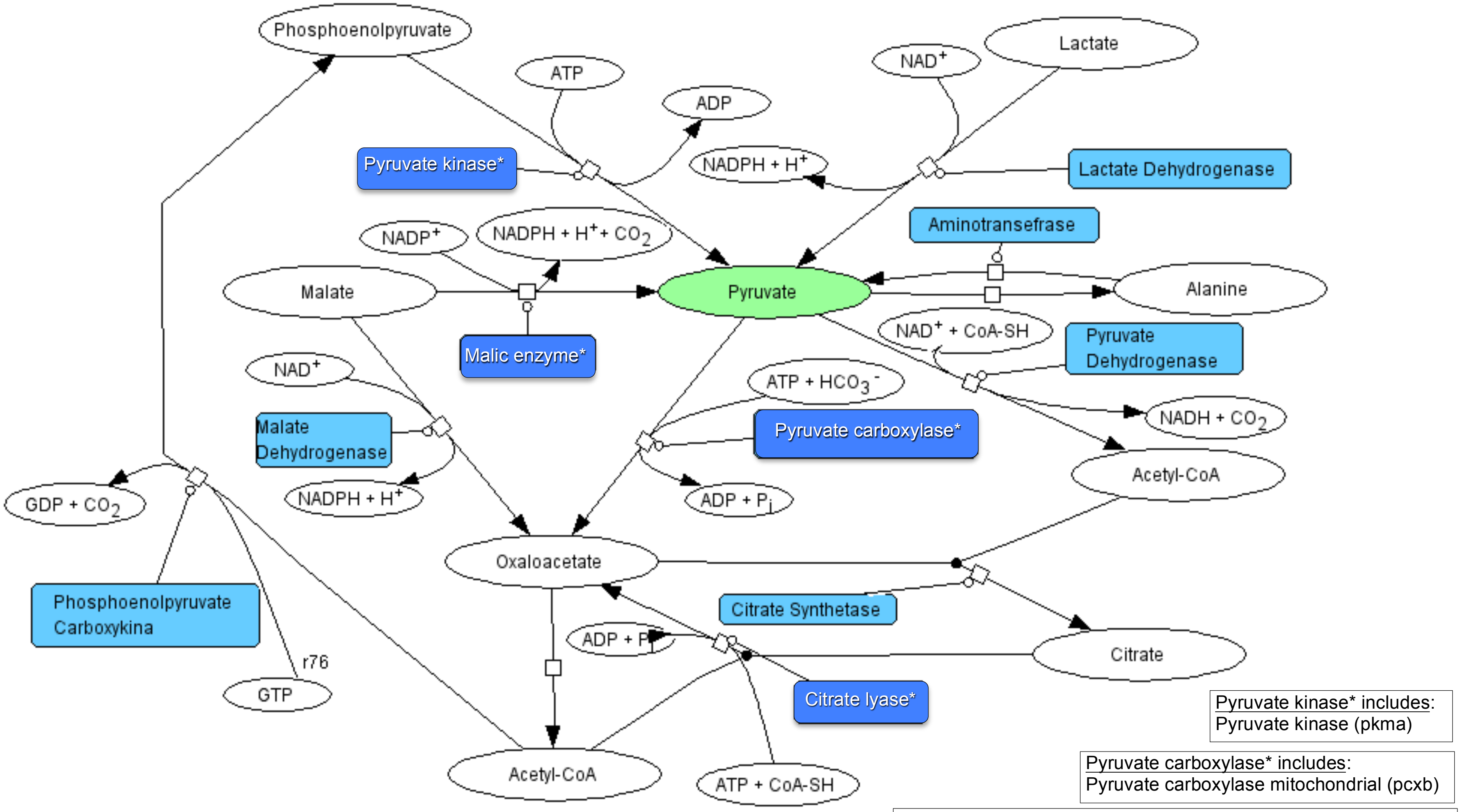

Pyruvate kinase* includes:
Pyruvate kinase (pkma)

Pyruvate carboxylase* includes:
Pyruvate carboxylase mitochondrial (pcxb)

Malic enzyme* includes:
NADP-dependent malic enzyme fragment 1 (LOC101 882012)
NADP-dependent malic enzyme fragment 2 (me1)
NADP-dependent malic enzyme fragment 3 (LOC101 882012)

Citrate lyase* includes:
Citrate lyase subunit beta-like protein mitochondrial (clybl)

Panel I. Proteins up-regulated > 2-fold in good quality zebrafish eggs, or unique to good quality eggs, in the Pooled Samples Experiment mapped to the Pyruvate metabolism pathway (Panther pathway P02772) in which they were found to be overrepresented (see **S3 Table**). The zebrafish proteins are indicated by the dark blue boxes with white text and are individually named in open boxes on the lower right with corresponding transcript names in parentheses. The mapped zebrafish proteins are pyruvate kinase (Pyruvate kinase*), Pyruvate carboxylase (Pyruvate carboxylase*), malic enzyme fragments (Malic enzyme*) and citrate lyase subunit beta-like protein (Citrate lyase).

De novo purine biosynthesis

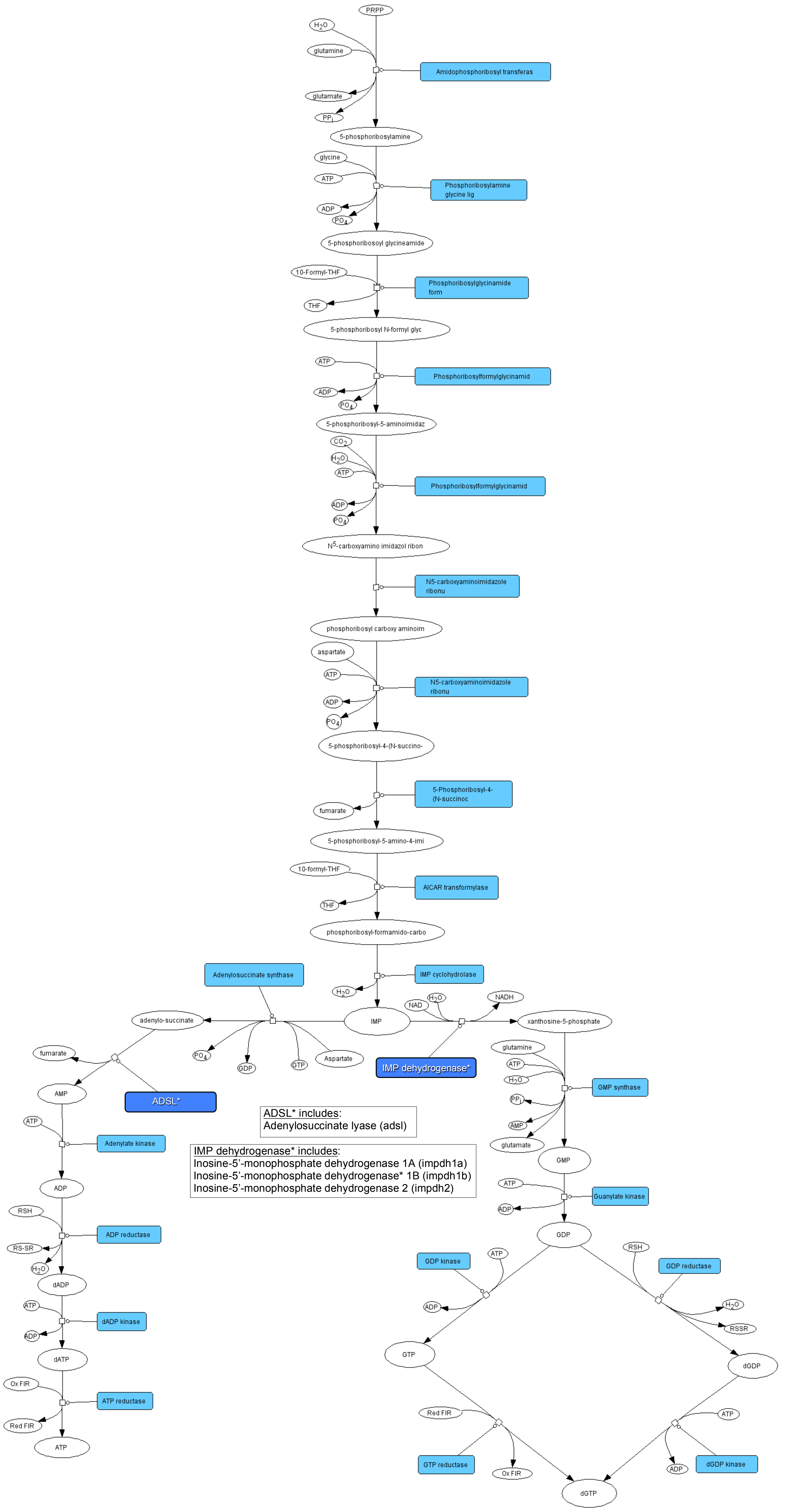

Panel m. Proteins upregulated $\geq$ 2-fold in good quality zebrafish eggs, or unique to good quality eggs, in the Pooled Samples Experiment mapped to De novo purine biosynthesis (Panther pathway P02738) in which they were found to be overrepresented (see **S3 Table**). The zebrafish proteins are indicated by the dark blue boxes with white text and are individually named in open boxes in the lower center with corresponding transcript names in parentheses. The mapped zebrafish proteins are adenylosuccinate lyase (ADSL*) and various forms of Inosine-5'-monophosphate dehydrogenase (IMP dehydrogenase*)

5-Hydroxytryptamine degradation

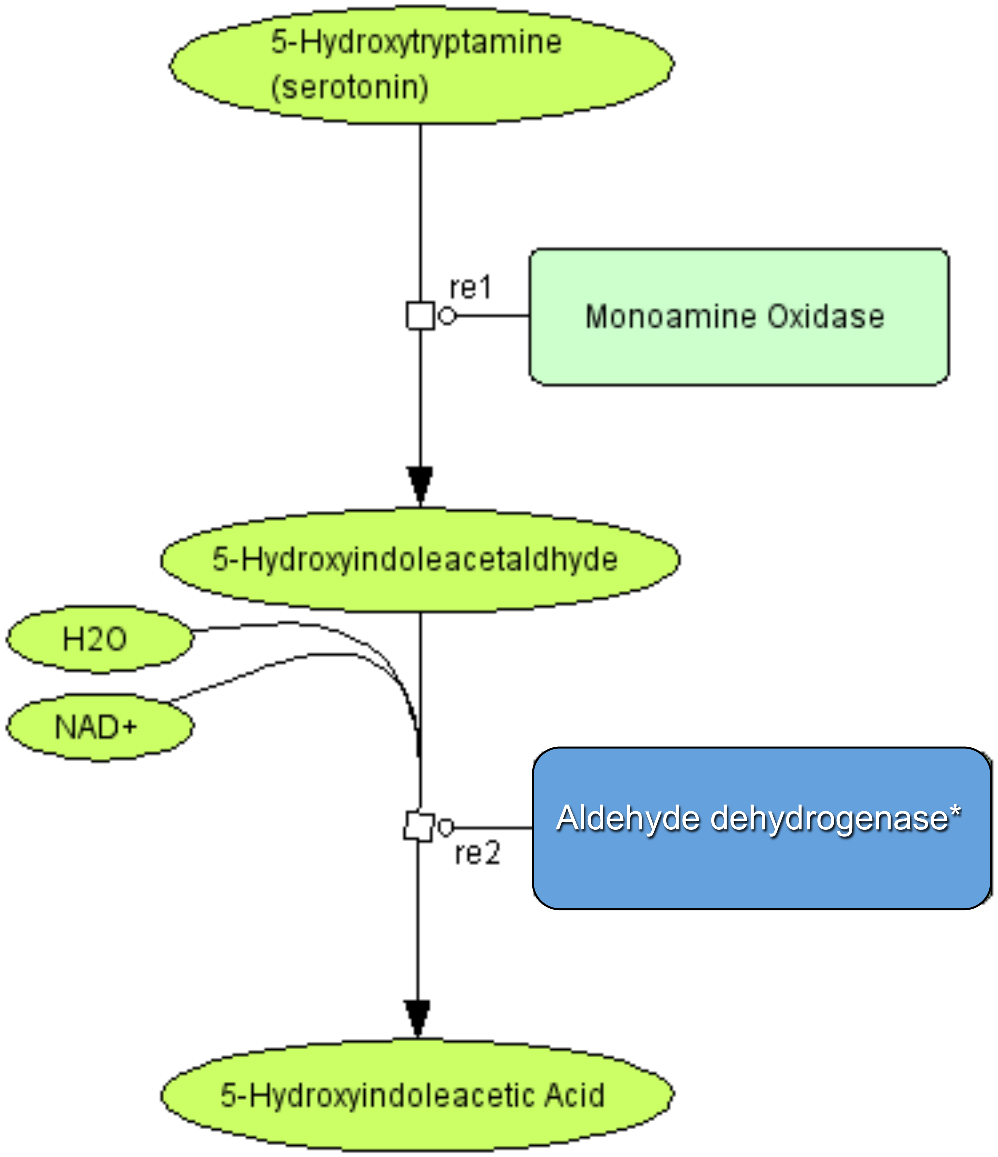

<u>Aldehyde dehydrogenase* includes</u>:
Aldehyde dehydrogenase 2.1 (aldh2.1)
Aldehyde dehydrogenase 2.2 (aldh2.2)
Aldehyde dehydrogenase 2-like (aldh2l)

Panel n. Proteins up-regulated $\geq$ 2-fold in good quality zebrafish eggs, or unique to good quality eggs, in the Pooled Samples Experiment or the Multiple Samples Experiment mapped to 5-Hydroxytryptamine degradation (Panther pathway P04372) in which they were found to be overrepresented (see **S3 Table**). The zebrafish proteins are indicated by the blue box with white text and are individually named in open boxes on the lower right with corresponding transcript names in parentheses. The mapped zebrafish proteins are all variants of aldehyde dehydrogenase (Aldehyde dehydrogenase*).
